# Mechanistic signatures of HPV insertions in cervical carcinomas

**DOI:** 10.1038/npjgenmed.2016.4

**Published:** 2016-03-16

**Authors:** Allyson Holmes, Sonia Lameiras, Emmanuelle Jeannot, Yannick Marie, Laurent Castera, Xavier Sastre-Garau, Alain Nicolas

**Affiliations:** 1 Institut Curie, PSL Research University, Centre National de la Recherche Scientifique UMR3244, Sorbonne Universités, Paris , France; 2 Department of Biopathology, Institut Curie, PSL Research University, Paris , France; 3 Institut du Cerveau et de la Moelle épinière (ICM), Genotyping and Sequencing Platform, Pitié-Salpêtrière Hôpital, Paris, France; 4 Department of Genetics, Centre François Baclesse, Caen, France

## Abstract

To identify new personal biomarkers for the improved diagnosis, prognosis and biological follow-up of human papillomavirus (HPV)-associated carcinomas, we developed a generic and comprehensive Capture-HPV method followed by Next Generation Sequencing (NGS). Starting from biopsies or circulating DNA samples, this Capture-NGS approach rapidly identifies the HPV genotype, HPV status (integrated, episomal or absence), the viral-host DNA junctions and the associated genome rearrangements. This analysis of 72 cervical carcinomas identified five HPV signatures. The first two signatures contain two hybrid chromosomal–HPV junctions whose orientations are co-linear (2J-COL) or non-linear (2J-NL), revealing two modes of viral integration associated with chromosomal deletion or amplification events, respectively. The third and fourth signatures exhibit 3–12 hybrid junctions, either clustered in one locus (MJ-CL) or scattered at distinct loci (MJ-SC) while the fifth signature consists of episomal HPV genomes (EPI). Cross analyses between the HPV signatures and the clinical and virological data reveal unexpected biased representation with respect to the HPV genotype, patient age and disease outcome, suggesting functional relevance(s) of this new classification. Overall, our findings establish a facile and comprehensive rational approach for the molecular detection of any HPV-associated carcinoma and definitive personalised sequence information to develop sensitive and specific biomarkers for each patient.

## Introduction

Each year, >500,000 individuals are diagnosed worldwide with human papillomavirus (HPV) associated cancers.^1^ HPV is a hallmark of most cervical cancers but is also associated with anal and head and neck carcinomas. Upon persistent, long-term infections of the cervical mucosa, oncogenic HPVs can cause high-grade cervical intraepithelial neoplasias (CINs), that without ablative treatment, may develop into invasive carcinomas.^2,3^ Better understanding of HPV biology has permitted major advances in the development of prophylactic vaccines and in the detection of HPV-associated diseases. However, cervical carcinomas remain frequent worldwide and their prognosis is poor, particularly in advanced stages and in cases of relapse.^4,5^ Notably, the efficiency of surgical removal of tumour relapses is related to the tumour size.^6^ It is thus of major importance to acquire molecular biomarkers that will facilitate the early diagnosis of cervical cancer, the detection of precocious tumour relapses and offer optimal biological follow-ups of the disease, upon and after treatment. Therefore, efforts to facilitate the diagnosis of HPV-associated cancers, elucidate the mechanisms driving HPV oncogenicity and develop cost-efficient and precise monitoring tools to improve clinical oncology, are needed.

**Figure 1:**
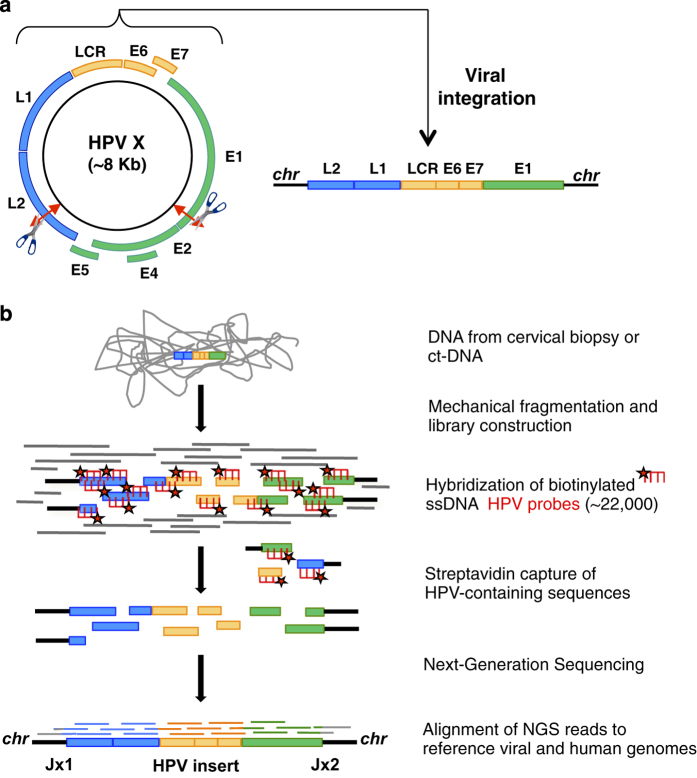
Capture-NGS method to identify HPV genotypes and integration sites. (**a**) The HPV dsDNA circular genome contains early (E) and late (L) replicating genes, necessary for viral replication and capsid formation. Integration and disruption/loss of the E2 suppressor gene sequences may lead to overexpression of the E6 and E7 viral oncogenic sequences, as well as disruption of host genomic DNA sequences. (**b**) Capture-NGS of HPV-integrated viral–cellular junctions. Genomic DNA is mechanically and homogeneously nebulised, followed by dsDNA library preparation and annealing of adaptor barcodes. HPV-biotinylated single-stranded probes (~22,000) then bind and capture HPV-containing library sequences that are enriched on a streptavidin column. These enriched DNA products are sequenced by NGS whose read alignments permit identification of the genotype, as well as the HPV-cellular hybrid junctions and localisation of the HPV insert in the tumour genome.

At the molecular level, HPV contains a 7.8-kbp double-stranded DNA circular genome (Figure 1a). One major difficulty in HPV detection and characterisation of tumours lies in the highly polymorphic nature (over 200 genotypes and variants) of this virus and in the sporadic nature of viral integration in the host cell. A subset of HPV genotypes, able to infect the mucosa of the ano-genital and oropharyngeal tract, have oncogenic properties and are classified as low, medium and high risk,^7^ among which the HPV16 and HPV18 genotypes are the most prevalent.^8–11^ Viral oncogenicity is largely related to their encoded E6 and E7 proteins, which have the ability to interact and inhibit the host tumour suppressors, p53 and pRB, as well as many other cellular protein targets.^12^ In CIN cases, the HPV DNA generally replicates extrachromosomally in the cell nucleus, whereas in most invasive carcinomas, a portion of the viral DNA is stably integrated into a unique site or perhaps within a few sites in the tumour cell genome.^13–20^ Upon integration, there is often a disruption of the E1/E2 genes, which causes constitutive expression of the E6/E7 oncoproteins. Viral integration may also lead to structural and/or modified expression of cellular genes nearby, as well as genomic amplifications, encompassing the HPV insertion site that may have a role in tumour progression.^21–24^ Along this line, the viral–cellular host junctions represent highly specific molecular markers of the individual patient tumours.^25^ However, PCR-based approaches used for junction analysis^26^ are difficult to implement in clinical oncology, notably due to a high rate of inconclusive and false-negative results.^23^ Most recently, a few genome-wide studies based on Next-Generation Sequencing (NGS) techniques such as whole genome, exome and targeted capture as well as RNA sequencing^17,20,27^ have been reported. However, these approaches remain limited and/or are difficult to implement in clinical practice, owing to technical complexities, biases towards certain HPV types, thresholds of bioinformatic filtering and/or cost efficiencies.

Here we describe a sensitive and exhaustive double-Capture HPV method, followed by NGS, designed to determine any HPV genotype and to detect cellular integration sites in HPV-associated carcinomas. This approach has been validated from a panel of 72 cervical patient cases, using tumour biopsy and circulating DNA from blood samples. Surprisingly, our patient-to-patient analysis of the junction reads identified five cellular HPV status whose signatures reveal new insights into the mechanisms of HPV integration and concomitant formation of large deletions or duplications at the insertion sites. Furthermore, these distinct HPV integration signatures prove to be relevant, both biologically and clinically.

## Results

### Capture-NGS of HPV DNA

The main steps of our Capture-HPV-NGS method are illustrated (Figure 1 and Materials and Methods). Briefly, genomic DNA from frozen biopsies and blood samples were extracted and the HPV-containing regions enriched using a double-capture method. Both rounds of capture consist of hybridisation with an exhaustive set of 22,000 single-stranded biotinylated HPV probes (Supplementary Table S1) in order to enrich for the small targeted viral sequences, which represent less than 8 kb of DNA out of >6 Gb of human DNA (an ~10^−6^ ratio). The probes were designed to detect the entire length of the HPV genomes from >200 HPV genotypes or variants, allowing an exhaustive and unbiased method to capture any chromosomally integrated, or episomally non-integrated HPV DNA sequence. The HPV-enriched DNA library was then sequenced to obtain single-end long reads (~500 nt average length) using Roche 454 Life Sciences (Roche Company, Branford, CT, USA) Junior or paired-end reads (2×150 nucleotides (nts)) using Illumina MiSeq (Illumina Inc., San Diego, CA, USA; Materials and Methods). These read lengths allowed the characterisation of the HPV type and of the insertion junctions. In 2 out of the 50 HPV-integrated samples (#83 and 202), one of the HPV–chromosomal junction was unambiguously mapped but the second junction read could not be definitively oriented as it overlapped a repetitive region of the human genome. To be noticed, as a proof of concept workflow, we successfully performed barcoded multiplexing of up to 7 and 12 patient DNA samples at once for the Roche 454- and Illumina MiSeq-sequenced samples, respectively. The NGS captured reads were subsequently aligned against all of the prototypical HPV genotypes, as well as the *hg19* human reference and analysed using standard mapping algorithms, compiled into a dedicated pipeline (Supplementary Figure S1, Materials and Methods). Extensive analysis of viral mutational events within the genome, including viral and cellular DNA characterisations, could thus be obtained.

### Capture-HPV provides full characterisation of the HPV status per patient

For each case, a single experiment permitted us to identify the HPV type, determine the number and architecture of the viral–chromosomal junctions, assemble the entire viral contig to characterise the pattern of the integrated viral DNA and assess the viral load through the read coverage.

#### HPV genotyping

Using ‘Capture-HPV’, we examined the DNA extracted from a biopsy panel of 72 cervical cancer patients, comprised of a heterogeneous population of women with respect to age, clinical stage and tumour size (Table 1 and Supplementary Table S2). We identified specific HPV genotypes in 70/72 cases: HPV16 in 34 cases (49%), HPV18 in 18 cases (26%), HPV31 in four cases (6%), HPV45 and HPV73 in three cases each (4.3%), HPV33 and HPV68 in two cases each (2.8%) and one case each of HPV6, HPV42, HPV51 and HPV52 (1.4%) (Table 1). These figures confirm the ability of our approach to characterize the two most prevalent worldwide HPV16 and HPV18 viral genotypes, as well as the next less common or rare types which vary by geographic region, such as HPV31 and HPV33 in Europe, HPV31 and HPV45 in Americas or HPV52 in Eastern Asia, HPV52 in Eastern Asia or HPV45 and HPV73 in Oceania.^28,29^ Of note, two cases of SCC associated with the low risk HPV6 and HPV42 genotypes were found. Importantly, even with the increase of sensitivity of the HPV capture, only one viral genotype was detected in each carcinoma case, demonstrating the clonal HPV status in each tumour. In two cases (No. 45, 46; Table 1), no HPV DNA sequences were detected upon mapping against the entire set of HPV variants using a permissive alignment threshold of 20 nts. Consistently, no specific PCR products were detected using a pair of HPV generic primers.^10^ These tumours are definitive examples of HPV-negative cervical carcinomas.

#### Heterogeneity of HPV inserts

**Figure 2:**
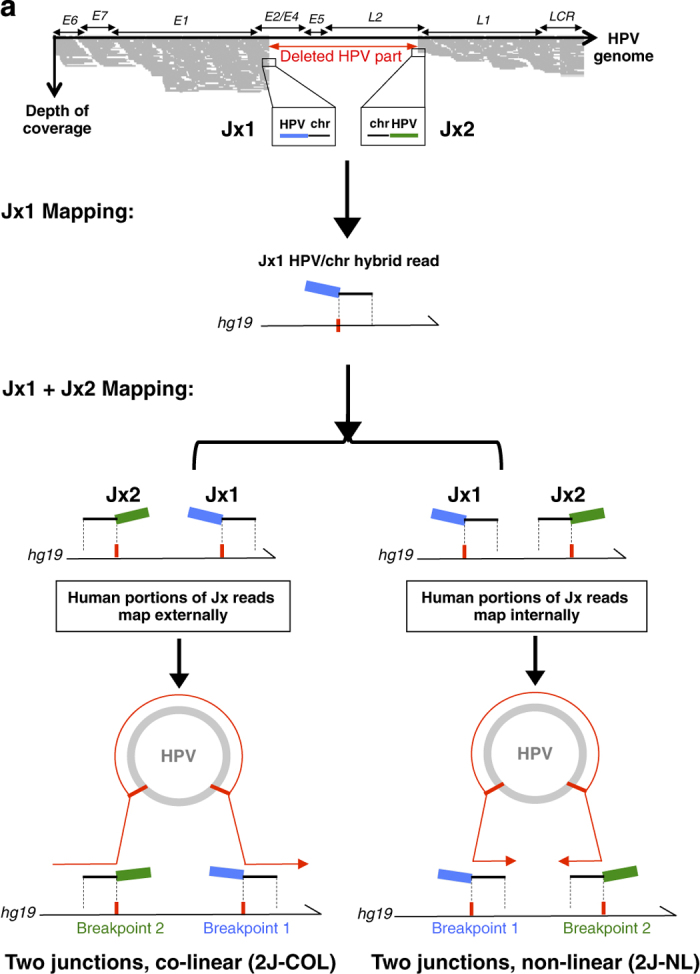

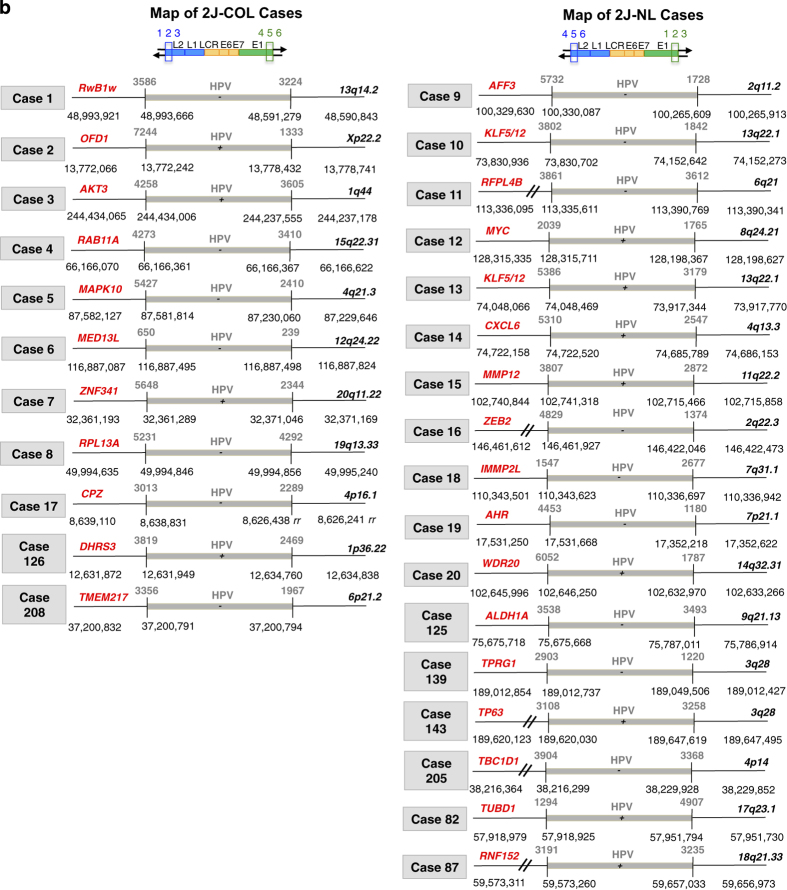

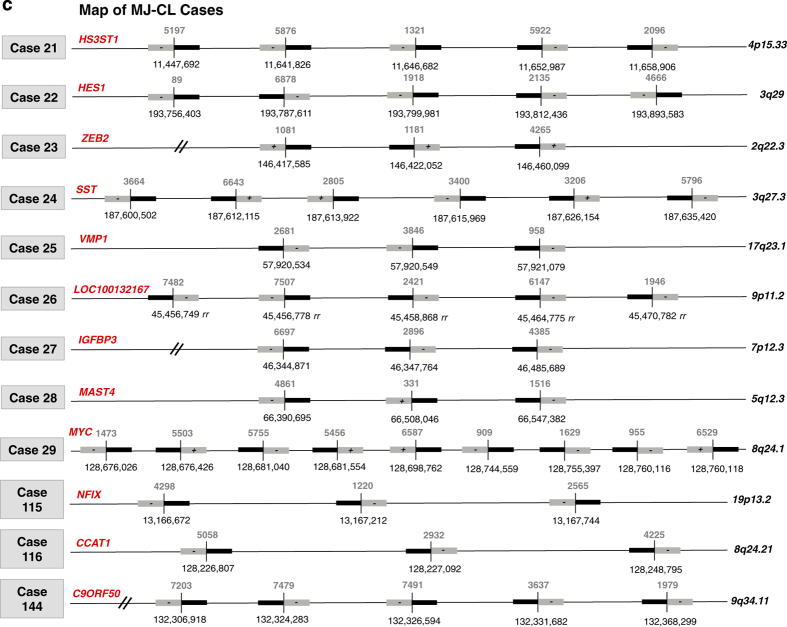

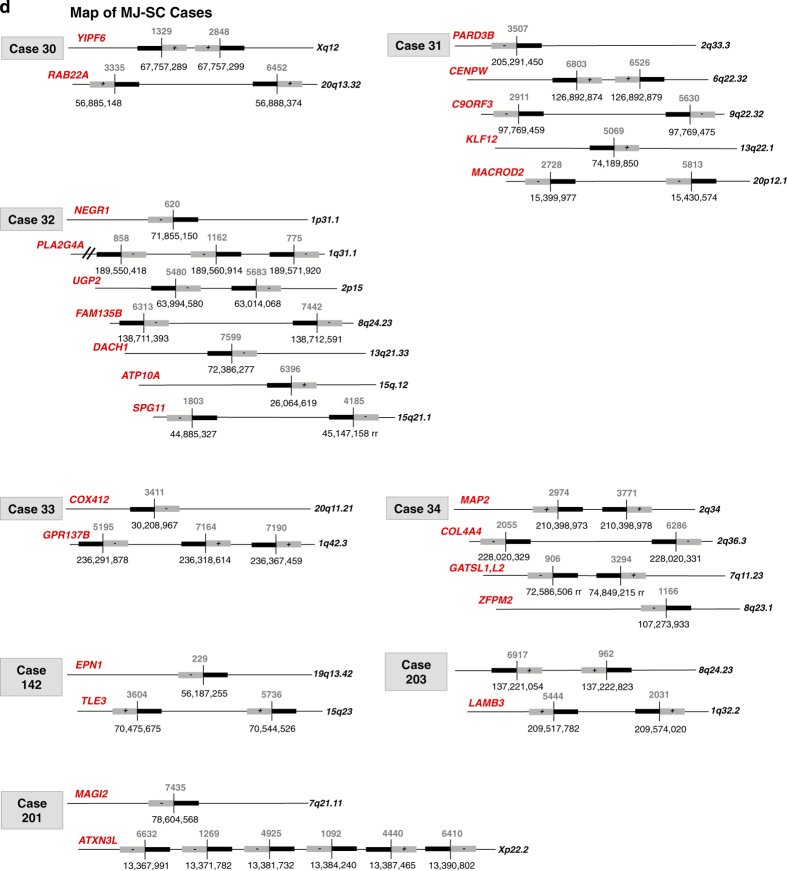
2J-COL and 2J-NL cases are defined by co-linear and non-linear mapping of the viral-human junctions. (**a**) The schematic of junction mapping is shown for cases with two junctions (2J-COL, for 2 junction co-linear mapping and 2J-NL, for 2 junction non-linear categories). The relative orientation of the two viral-human junction reads are identified by mapping the human portion of the sequences against the *hg19* reference genome. (**b**) Junction mapping of 2J-COL cases (left panel) and 2J-NL cases (right panel) are shown, with a scheme indicating the co-linear and non-linear coordinate mapping for these categories. The coordinates of the human (junction to chromosome) and viral (junction to junction) NGS reads are indicated, as well as the chromosomal locus of insertion. At least one gene within 500 kb or greater (//) is indicated. In all cases, the human part of the NGS junction reads was mapped against the forward strand of the *hg19* reference. The HPV sequences in either plus or minus orientations, relative to the viral episome, are shown as a grey box with (+) or (−). (*rr*) indicates repetitive regions. (**c**) Junction mapping of MJ-CL cases are shown. The human/viral breakpoints are shown as well as the chromosome locus of insertion. Black boxes indicate the human portion of the junction reads. (**d**) Junction mapping of the MJ-SC cases are shown, as in (**c**).

Looking at the 50 integrated cases (2J and MJ categories, Figure 2a; Table 1), we noted that the viral reads did not map uniformly along the entire HPV sequence but instead, several gaps identified as junction reads were observed, reflecting the presence of a deletion in the viral genome. Altogether, we found that a portion of the HPV genome, ranging from 25.2 to 96.8%, is inserted into the human genome (Supplementary Table S2). The breakpoints occur most often within the early E1–E5 and late L1–L2 genes. All viral inserts contain the HPV origin of replication (ORI), located in the LCR region, suggesting that the process of integration may have positively selected for the retention of these elements. Most viral inserts contain disrupted or deleted E2-coding regions while retaining the E6 and E7 genes, a feature consistent with enhanced expression of the E6 and E7 oncogenes via the inactivation of the E2 repressor upon integration. However, unexpectedly, in one HPV51-associated case (No.6), the two breakpoints were located within the E6 and E7 oncogenes and no evidence of associated episomal HPV genomes was observed. Also unexpectedly, in two other cases (No. 12, 18), the E1 region was disrupted but the E2 gene remained intact, indicating that E2 disruption is not always associated with HPV integration. In three additional cases (No. 22, 28, 32), disruption of the E6/E7 oncogene was also found in a sub-set of the reads detected. These data outline the unappreciated and large heterogeneity of HPV sequence insertion patterns amongst different tumours, which validates the overall benefit of using our comprehensive and unbiased set of Capture-HPV probes.

#### Architectures of HPV insertions

To better understand the nature of the HPV integration events, we examined the viral-chromosome junction sequences and the integrated HPV content. The analysis of our NGS reads was performed on a patient-to-patient basis that provided a puzzling heterogeneity of HPV signatures and crucial mechanistic insights. This is reflected by the variable number of hybrid junctions captured per sample and their chromosomal distributions, being clustered at the same chromosomal locus or scattered at different loci. In terms of the number of breakpoints, we identified two hybrid junctions (2J) per tumour in 30/70 cases, indicating a simple integration pattern of a truncated viral genome into a single chromosomal locus (Table 1 and Supplementary Table S2). Most interestingly, close examination of these 30 cases led us to focus on the relative orientation of the viral-chromosomal junction reads. As illustrated in Figure 2a, two classes of carcinomas, harbouring two hybrid junctions could be identified: In 11 cases (No. 1–8, 17, 126, 208), the upstream and downstream junction reads were in the expected orientations for a simple co-linear HPV integration event, i.e.,; in the orientation chromosome-HPV at the upstream breakpoint and HPV-chromosome at the downstream breakpoint (Table 1, Figure 2b left panel). In contrast, 17 cases (No. 9–16, 18–20, 82,87, 125, 139, 143 and 205) displayed a non-linear alignment pattern, meaning that the upstream and the downstream junctions reads mapped to the human genome in the orientation HPV-chromosome and chromosome-HPV, respectively (Table 1, Figure 2b right panel). Hereafter, these distinct viral insertion architectures, containing two junctions are referred to as 2J-colinear (2J-COL) and 2J non-linear (2J-NL), respectively. In the remaining cases (No. 83, 202) two junctions were also identified but one of them being in a repeated region of the genome, their relative orientation could not be determined. In a third category (12 cases; No. 21–29, 115, 116, 144; Table 1, Figure 2c), the architecture of insertion exhibited 3–9 distinct hybrid viral-cellular junctions, each validated by at least five independent and non-overlapping reads, that were clustered in the same chromosomal region. This multiple junction and clustered pattern was abbreviated as MJ-CL. A fourth pattern (8 cases; No. 30–34, 142, 201, 203; Table 1, Figure 2d) was characterised by the presence of several hybrid junctions (4–12) in which the chromosomal sequences aligned ectopically to unlinked and distant regions on the same chromosome or were scattered among 2 or more chromosomes. These cases are referred to as the multiple junction scattered (MJ-SC) category. On the basis of the analysis of the individual HPV polymorphisms, a single viral genotype was detected in all junction reads of the same tumour, again underlining the clonal and personalised HPV signature in each tumour. The final category (20 cases) includes high-coverage NGS reads containing full-length HPV genotypes and no hybrid reads represented by more than one NGS read. Therefore, this category, named EPI, corresponds to cases with only high copy episomal HPV genomes, without clonal integration (Table 1). To be noted, in one of these samples (#132), we detected 16 non-duplicate overlapping HPV-chromosomal reads (0.3%) suggesting that one copy of HPV was already integrated in a subset of the tumour cells. Our capacity to detect such heterogeneity is likely owing to the high sensitivity of the capture method that allows to reach very high sequencing coverage (up to several thousand folds). To confirm our NGS results, we performed multiple PCR Sanger analyses of the junctions belonging to categories 2J-COL, 2J-NL, MJ-CL and MJ-SC, confirming all junctions in the 2J categories and most junctions in the MJ categories. We then analysed homologies between the viral and cellular sequences at the breakpoint fusions. We detected no significant continuous homology lengths but rather microhomologies at and surrounding these breakpoint sites, an important feature, also described by others,^20^ that should be considered when deciphering possible mechanism(s) of HPV viral integration (Supplementary Figure S2).

#### The distinct architectures of HPV insertions are also detected in circulating DNA

The above case studies utilised DNA from biopsy samples, allowing the identification of pertinent personalised genomic biomarkers. However, a tumour biopsy is an invasive procedure as compared with taking a blood sample. For the same reason, the analysis of circulating tumour DNA (ct-DNA) samples would also be preferable for the optimal follow-up of patients during and after treatment. Therefore, we investigated whether our Capture-HPV method could also detect the HPV integration signature biomarkers from blood samples. We analysed the ctDNA (Materials and Methods) of serum specimens taken at the time of diagnosis in five patients in which the 2J-COL (cases 5,7), 2J-NL (cases 18,20) and MJ-CL (case 29) signatures had been found, respectively. Capture-HPV from serum/plasma specimens was conducted as for biopsies (Materials and Methods) and yielded numerous HPV and viral–human hybrid NGS reads. Most importantly, the same viral-junction patterns were identified in the blood and tumour samples from each patient (Supplementary Figure S3). Furthermore, as for the biopsies, the coordinates of the paired junction reads delineate the associated chromosomal rearrangement at the HPV insertion site. Thus, the Capture-HPV approach is a powerful and robust method to detect HPV and characterise its genotype, physical status and integration loci, as well as the coordinates of the associated structural alterations. It should be emphasised, that the detection of ct-DNA by Capture-NGS does not require prior analysis of the matched tumour specimen to identify the desired tumour-specific molecular biomarkers.

#### Viral load and integration patterns

The viral load was measured with two different methods. Classically by quantitative PCR of the E7 gene copy number (Supplementary Table S2) but also from the quantitative analysis of the NGS coverage per nucleotide of the HPV genome and three house-keeping control genes (*GAPDH*, *KLK3* and *RAB7A*) captured in the same tube of probes (Materials and Methods). As illustrated in Supplementary Figure S4A, both methods correlate very well (*R* ^2^=0.93 and *R* ^2^=0.87 for the 454 and Illumina sequenced samples, respectively). Also, the quantitative analyses of the reads led us to observe a positive correlation between the integration signature pattern and the absolute number of purely viral reads detected (Supplementary Figure S4B, *R* ^2^=0.61). The lowest number of reads (mean 16,559 reads per sample) was observed in cases with two junctions (2J-COL and 2J-NL), and increases (mean 90,754) in tumours with multiple junctions (MJ-CL and MJ-SC). The highest number of reads (mean of 181,742) was observed in the EPI cases, a feature indicating a high copy episomal HPV content, consistent with the high viral load also detected by quantitative PCR (Supplementary Table S2, Supplementary Figure S4B).

### Viral integration architectures are also characterised by distinct genomic rearrangements

**Figure 3:**
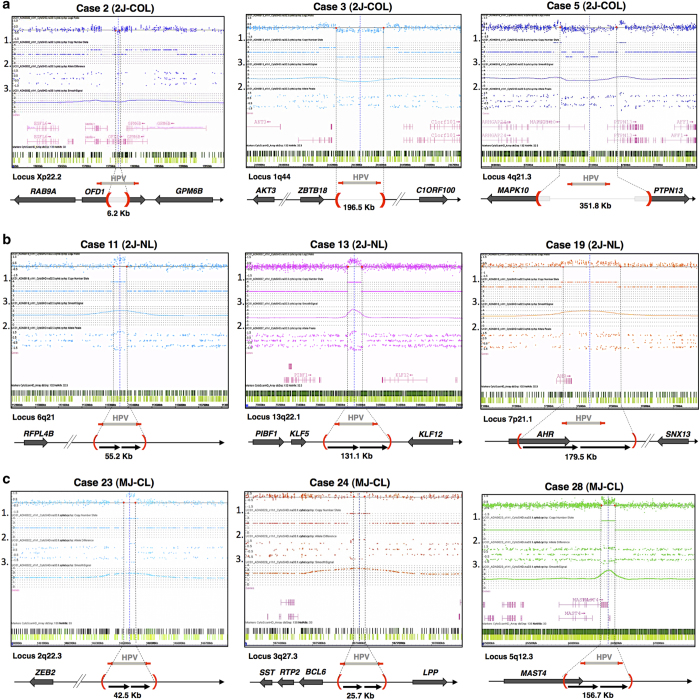

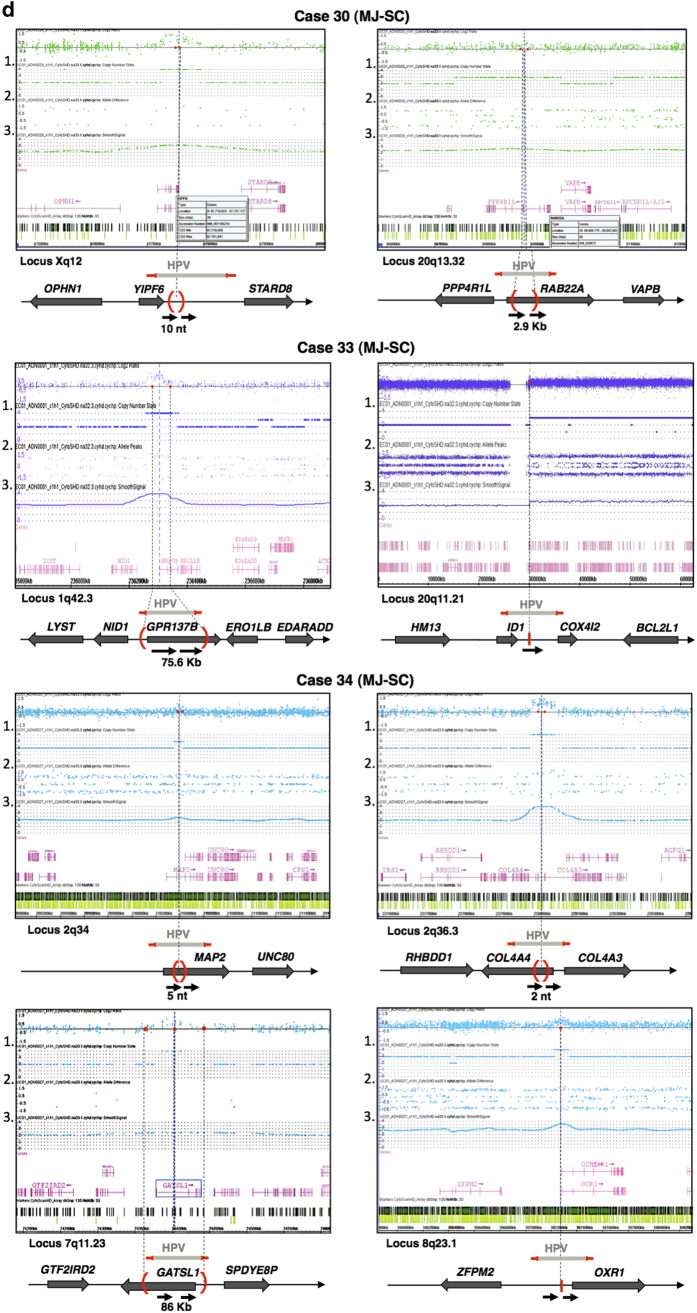
Analysis of host-genome copy-number changes at HPV insertion sites. (**a**) CytoScan HD microarray (Affymetrix) and ChAS analysis (v 3.0) were performed from cervical tumour DNA to detect copy-number changes and structural alterations at sites of HPV insertion. Three 2J-COL cases are shown. The ChAS profile for each case indicates the HPV insertion breakpoints (dashed vertical lines), copy-number state (1), allele peak difference (2) smooth signal (3.) and density of probes (light/dark green bars). The chromosome locus and nearby genes are also shown. The Cytoscan data of the other 2J-COL samples are shown in Supplementary Figure S5. (**b**) Analysis performed as described in **a**, from three 2J-NL cases. The Cytoscan data of the other 2J-NLsamples are shown in Supplementary Figure S6. (**c**) Analysis performed as described in **a**, from three MJ-CL cases. (**d**) Analysis performed as described in **a**, from three MJ-SC cases.

The distinction between 2J-COL or 2J-NL hybrid junctions raised the question of whether these two architectures might have resulted from different mechanisms of HPV integration. We first examined the physical distance between the breakpoint junctions on the reference human genome. It ranged from one base pair (case No. 8) up to 402.4 kb (No. 1; Table 1, Supplementary Table S2). Remarkably, they are often closer to each other in the 2J-COL (⩽5 kb in 6/11 cases) and more distant in the 2J-NL (⩽5 kb in 0/17 cases). These observations raise the important question of what happened to the recipient chromosomal region and whether this could provide clues on the mechanism of integration. To characterise the eventual associated rearrangements, we used a genome wide CGH-array approach that allows high-resolution (5 kb) determination of a copy-number change (Materials and Methods) implicating the region bracketed by the HPV insertion breakpoints identified by capture, and also detects chromosome gains/losses throughout the entire genome. We first examined the 2J-COL and 2J-NL categories. Concerning the 2J-COL events, in 5/11 cases, the distance between the HPV breakpoints were ⩽5 kb. In the other cases (No. 1–3, 5, 7) the chromosomal copy-number decreased about 1.5-fold between the two HPV-cellular junctions (Figure 3a), indicating that the intervening chromosomal sequences between the breakpoints have been deleted, and that the insertion of HPV occurred on one of the chromosome pairs. In the first case (No. 2) we observed a 6.2-kb deletion directly within the *OFD1* gene (Xp22.2). In the second case (No. 3), a 196.5-kb deletion is located near the *ZBTB18* and *C1ORF100* genes (1q44). Finally in the third case (No. 5) we found a 351.8-kb deletion leading to the partial deletion of both the *MAPK10* and *PTPN13* genes (4q21.3). Among the remaining cases in the 2J-COL category, we detected two additional deletion cases (No. 1, 7) as well as flat CGH profile cases (No. 4, 6, 8, 17, 126) and one flat profile, bordering on a chromosomal amplification (208), consistent with the observation that the interval between the breakpoint is small (1–6 nts) and the deletion event is not detectable by CGH (Supplementary Figure S5).

In sharp contrast, most 2J-NL events (15/17) exhibited a 1.5 to 4-fold increase of chromosomal copy number between the two HPV-cellular junctions (Table 1, Figure 3b, Supplementary Figure S6). In the two remaining cases in which the distance between the breakpoints is small (25 and 36 kb), the CGH profile was flat. These results reveal that the 2J-NL HPV insertion signature is associated with an amplification of the human genome interval, located between the HPV insertion breakpoints detected by capture NGS. Similarly, we performed CGH array analysis on all other MJ-CL (Figure 3c), MJ-SC (Figure 3d) and EPI cases (Supplementary Figure S7). Within the MJ-CL category, we found either amplifications (1.5 fold up to 4 fold, No. 21–24, 28–29, 115–116) or a flat profile (No. 25–27; Figure 3c; Supplementary Figure S8). In the MJ-SC cases, we found a mixture of either amplified or flat profiles at the different junctions in three cases (No. 31, 32, 203), whereas the three other cases (No. 30, 33, 34, 142, 201, 203) showed amplifications at each of the viral insertion loci (Figure 3d). In addition, HPV DNA was often found to co-localise at the transition boundaries between lengthy chromosomal gains and losses (No. 33, chromosome band 20q11.21, see Figure 3d). The loss of heterozygosity observed in the vicinity of HPV insertion sites points to a mechanism of integration involving a break-induced replication type event. In sum, strikingly, no deletions were detected at HPV insertion sites in any category other than 2J-COL suggesting that, all of the other events result from the same ‘ends-in’ mechanism of chromosome sequence assimilation (see below). We also assessed by FrAGL analysis (Methods), the frequency of amplicon gain or loss and copy-number variations genome wide, among all cases. No additional striking genomic differences were observed among the signature groups (Supplementary Figure S7).

Overall, the cases within the 2J-COL category share, as a common structural feature, the deletion of the chromosomal region within the interval of the two breakpoints. In contrast, the 2J-NL and MJ categories only show local amplifications. Amplification events at the HPV insertion locus have been previously described,^17,23,27^ but to our knowledge, this is the first comprehensive analysis integrating the relative architecture of the two junctions in individual tumours and the detection of a deletion pattern arising from HPV integration. In contrast, to the usually large amplification events that are likely to affect several adjacent genes, the small size of the associated deletions are more likely to yield a single-gene dysfunction, more amenable for the design of precision therapeutics.

### Annotations of the HPV insertions

As previously observed, the viral-host junction reads in HPV-associated carcinomas map at multiple locations in the human genome^15,17,20,23,30^ Herein, we identified 175 robust viral–chromosomal sequence fusions (Supplementary Table S2 and Supplementary Figure S10). Among the 50 insertion cases, viral sequences were found within a coding sequence in 29 of these (58%) or within <500 kb from a known gene in 18 cases (36%; Table 1). In the remaining cases, HPV was inserted in integenic and repeated regions of the genome (Table 1). Recurrent integration events were found at the *KLF5/KLF12* locus (five cases), and near *MYC* (three cases). Among the genes not previously reported in the literature as directly targeted by HPV integration, we found *RB1*, *AKT3*, *SST* and *ID1*, implied in the regulation of cell proliferation, *LPP* in cell adhesion/motility, transcription factors such as *AFF3*, *BCL6*, *CCAT1* and *CCAT2*, and oncogenes such as *RAB11A* and *RAB22A* (Supplementary Figure S11). Among the potentially deregulated genes located near HPV DNA, we noted *MAST4* and *MAP2* implicated in the regulation of microtubules, *MMP12* and *COL4A4* coding for matrix components and *PF4V1* in angiogenesis. Altogether, our data identify new cellular targets for viral integration in HPV-associated carcinomas and confirms that integration sites, though widely distributed, are not random, being most likely mechanistically favoured and/or functionally selected when carcinogenesis ensues.

### Clinical annotations related to HPV integration signature

**Figure 4:**
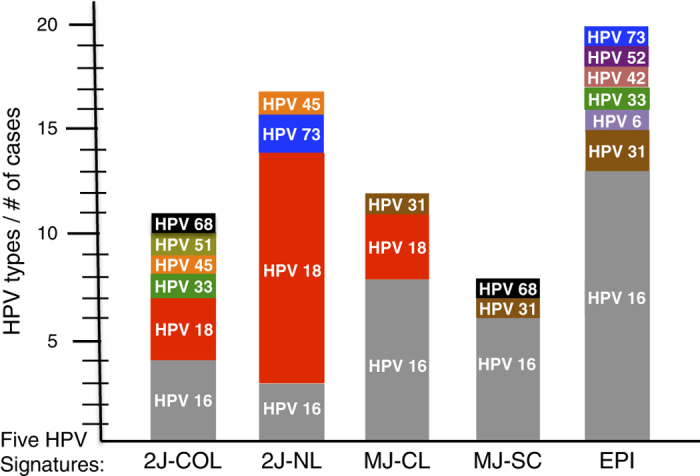
Genomic and clinical cross-analyses. Among all HPV positive cases, the different genotypes identified are shown as the number of HPV genotypes per case. Each case is also grouped according to their genomic integration signature group: 2J-COL; 2J-NL; MJ-CL; MJ-SC and EPI.

In order to assess the relevance of our classification of cervical carcinomas, we further compared HPV signatures with biological and clinical parameters. No significant correlation was observed between the integration pattern, tumour size (17–80 mm), histology and clinical staging (I-IV). In contrast, a strikingly unbalanced distribution of HPV genotypes was observed according to the integration signatures. Unexpectedly, a majority (11/18) of the HPV18 associated cases were found in the 2J-NL group whereas only three of the most prevalent HPV16 genotypes (a total of 49% in the present series) exhibited this signature (Figure 4). In this category, we also detected one HPV45 case (that has 81.7% sequence identity with HPV18, Supplementary Figure S12) and two HPV73 cases. Also striking, we found heterogenous genotypes in the EPI category but no HPV18 nor HPV45.

Another aspect that we analysed concerns the age of the patients. The clinical data from our case panel gave a median patient age of 48 years. The median age was 48 in patients with 2J-COL tumours, 45 in 2J-NL patients, 45 in MJ-CL, 52 in MJ-SC and 56 in women with EPI carcinomas (Supplementary Figure S14). Altogether, an 8–11 year difference in median age was observed between the 2J and EPI classes (Pairwise comparisons using Mann–Whitney Wilcoxin rank sum test, 2J-COL versus EPI, *P*=0.264; 2J-NL versus EPI, *P*=0.099; MJ-CL versus EPI, *P*=0.185; and MJ-SC versus EPI, 0.959) and between all the integrated versus EPI, the *P* value is 0.12. Finally, follow-up data recorded at least 6 months after diagnosis were available for 58 cervical cancer patients. Half were either deceased (14/29), diagnosed with pelvic relapse (10/29) or metastasis (3/29). By comparing the HPV signatures with the disease outcomes, we noted that a poor outcome was more frequently recorded in tumours from the 2J category (17/24) than in the EPI (9/17) and MJ (5/17) categories (Chi-square test: *P* value integrated versus EPI=0.12; Supplementary Table S2). Further studies including a larger number of cases analysed in the present way are required to assay the robustness of this intriguing distribution for its potential prognostic value. The ability to determine the HPV mode of integration upon diagnosis of the pathology, will be of significant value for the follow up of the patient.

## Discussion

By studying tumour DNA and ct-DNA from cervical carcinoma patients, we validated a cutting-edge NGS method able to detect and characterise all tumour-associated HPV sequences. Our approach provides new insights into the mechanisms of HPV integration as well as its diverse structural consequences on the tumour cell genome at the insertion locus. Most importantly, this work has led to the classification of HPV-associated carcinomas into five categories that are potentially relevant both biologically and clinically. As such, HPV-associated tumours represent a highly favourable model for the development of genomic medicine.

Compared with PCR-based methods for HPV typing, the comprehensive Capture-HPV approach is unbiased and able to provide single-nucleotide level information, as deep genome-wide based-sequencing approaches, provide.^17,20,27^ Further, our approach is rather cost efficient (few hundred dollars per sample), rapid (few days without the need to reiterate PCR attempts), technically relatively simple for a molecular biologist, and is therefore able to be implemented today in clinical settings, with the benefits of providing in one experiment: (i) the capture of any HPV genotype regardless of its prevalence in the population, (ii) its full sequence identity by the *de novo* assembly of the NGS reads, (iii) the viral load assessment as measured by the read coverage normalised against house keeping gene(s) included in the capture probes, (iv) the physical status, integrated or episomal of the viral sequences, (vi) the pattern of integration into the diverse chromosomal insertion sites, (vii) the sequence of the hybrid viral–chromosomal junctions that provide exquisitely specific biomarkers, as well as (viii) the nature and coordinates of the associated chromosomal deletions or amplifications. This method also provides insights into the tumour biology and potential therapeutic opportunities to treat HPV-associated cancers, by characterizing the status of the HPV E6/E7 oncogenes and their E1/E2 repressor as well as all other possible viral rearrangements and the identification of the gene(s) that are directly or indirectly altered *via* HPV insertion.

**Figure 5:**
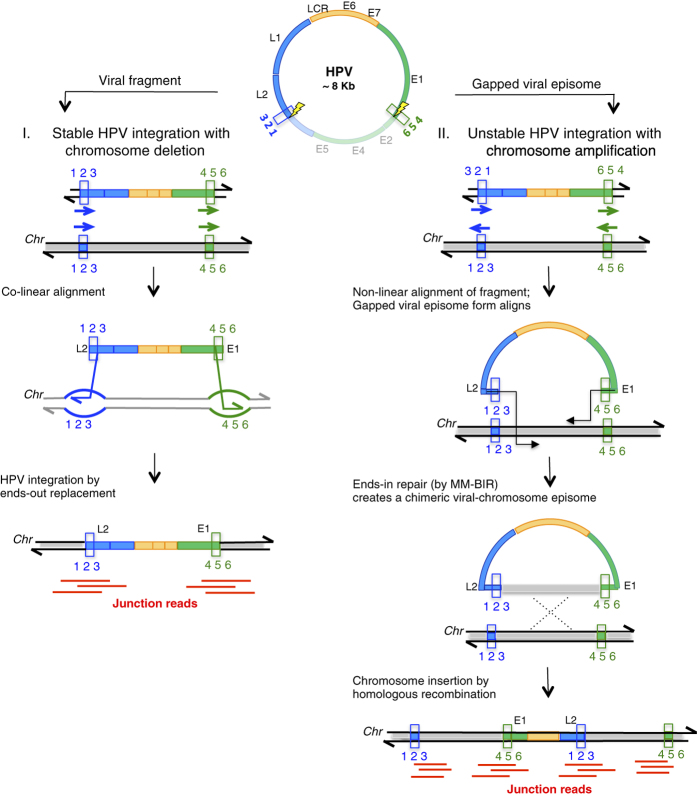
Ends-out and Ends-in HPV integration mechanisms. In the left panel (ends-out repair), the two linearised microhomologous 3’ ends of the HPV fragment align co-linearly with the target chromosome and perform strand invasion by ends-out replacement of the chromosome sequence with HPV, resulting in a stable deletion of the intervening host sequences. In the right panel (ends-in repair), the two microhomologous 3′ ends align to the target chromosome sequences, only when HPV is in a gapped episomal form. The 3’ ends of the gapped episome can invade and initiate synthesis from the target sequence, creating a chimeric viral chromosome. Repair synthesis occurs by microhomology mediated break-induced replication pathway (MM-BIR). The resulting viral-chromosome chimeric episome can then integrate by homologous recombination into the same copied target sites, resulting in chromosome duplications and amplifications, a transient and recurrent event, predicted to be unstable.

In a pioneer study, the group of E. Schwarz developed a valuable approach combining ligation-mediated-PCR with NGS to specifically detect HPV16-associated sequences.^17^ Integration of HPV16 DNA was observed in 78.8% of their cases, which correlates well with the 71.4% cases of integration found in our series, collected in two French hospitals. In comparison with previous approaches, one of the assets of Capt-HPV is the incorporation of a large set of probes representing >200 HPV genotypes and variants that allowed us to detect and identify all HPV sequences, including those of various worldwide geographic prevalences such as HPV16, HPV18 and HPV45 in America, HPV45 and HPV33 in Africa or HPV52 in Eastern Asia,^28,29^ as well as their status of integration. Furthermore, our case-by-case analysis of the HPV sequences led us to identify 5 HPV signatures distinguished by the relative orientation of the hybrid viral–cellular junctions (2J-COL versus 2J-NL), the proximity of HPV breakpoints in the 2J-COL category and their larger spacing in 2J-NL, the multiplicity of junction reads in certain tumours (MJ-CL, MJ-SC) and finally the presence of the viral genome in the episomal state only (EPI). Remarkably, the distinction between the 2J-COL and 2J-NL integration patterns was independently validated by two characteristic genomic alterations found at their respective loci, namely deletions (2J-COL) or amplifications (2J-NL). These correlated features can be simultaneously explained by two modes of integration. In the first mode, the two ends of the linearised intact gapped viral DNA align to the host target, such that by ‘ends-out’ replacement, the virus is inserted creating a deletion of the intervening chromosome segment, explaining the formation of the 2J-COL architecture (Figure 5, left panel). This is a model of DNA double-strand break repair proposed to explain plasmid/fragment integration in yeast.^31^ Not surprisingly, this mechanism of integration that does not require extensive strand synthesis, favours closely spaced interactions of the HPV ends-out DNA ends, with nearby chromosomal regions. In the second mode of integration, the two ends of the linearised intact or gapped viral DNA, will invade the host target in an ‘ends-in’ mode, involving template directed DNA synthesis (Figure 5, right panel), explaining the formation of the 2J-NL architecture and the opportunity for each HPV end to independently interact with distant regions of the chromosomal regions. This leads to the copy (via gap repair synthesis) of the target sequences at variable lengths, resulting in capture of the target sequence incorporated into the viral DNA episome, creating a chimeric viral–chromosome intermediate. Next, as part of an amplification ‘looping’ model^27^ the initiating intermediate will be able to form DNA concatemers and/or integrate into the chromosomal insertion site by homologous recombination, thus explaining concomitant duplication(s)/ amplification(s) at the target sequence. In our seventeen 2J-NL cases, the length of the amplified regions varies from ten to several hundreds of kilobase pairs. This can be explained by the variable efficiency of gap repair synthesis, as observed in other assays in yeast and mammalian cell cultures.^32^ The limited homology we observed at the final viral–chromosomal junctions (Supplementary Figure S2), is attributed to microhomology-mediated primer extension events, as proposed in the MM-BIR (microhomology-mediated break-induced replication) model.^33^ With similar molecular steps, the MJ cases may occur if the resolution of the viral integration events are not always efficient, allowing repetitive cycles of viral chromosomal insertions that lead to the formation of integrated concatemers, as represented in the MJ-CL category. An alternative, yet non-exclusive hypotheses, is that both ends of the invading virus behave independently. Subsequently, these ends may capture distant chromosomal sequences that ultimately create the complex set of junction events that are detected in the MJ-CL and MJ-SC categories. An important outcome of this scenario will be to copy and assemble together one or several physically distant chromosomal fragments. Thus, the identification of several host junctions in a single tumour would not necessarily mean that HPV integrated within several places and in several copies per genome, but rather integrated in a single locus that brought together a complex set of captured host sequences, upon multiple rounds of microhomology-mediated capture of chromosomal fragments. A recent study^20^ found 3,666 HPV integration breakpoints in 103 cervical carcinomas or cell lines, but whether or not these breakpoints reflected single or multiple HPV insertions, remains to be established.

To estimate the impact of our HPV tumour classification system, we analysed the available patient clinical and biological data. The first unexpected finding was that, within the 2J-NL category, the majority (11/17) of the HPV18-associated cases were found in this group, whereas only 3/21 of the highly prevalent HPV16 genotype (here identified in 49% of all tumours) exhibited this signature (Figure 4). Also, no HPV18 was observed in the EPI category (Figure 4). In addition, the mean number of single-nucleotide variations within HPV16 is highest in the MJ-SC (17 nt) and EPI (14 nt) cases (Supplementary Figure S13A), with a different distribution of polymorphisms found among the groups along the entire sequence, as compared with the reference HPV16 sequence (gi|333031|lcl|HPV16REF.1) from the PaVE genome site (Supplementary Figure S13B). These polymorphic differences among the HPV16 cases in these groups may influence their oncogenic potential. The reason why the integration of HPV18 occurs according to a non-linear pattern associated with local amplification is not yet clear. Perhaps HPV16 and 18 genotypes have different mechanisms of integration (amplification versus deletion) due to their different regions and lengths of microhomologies between the viral and host genome. The second intriguing result concerned the striking age gap (up to 11 years) between the various HPV integrated and EPI categories (Supplementary Figure S14). The statistical comparison between these categories is not significant (Mann–Whitney *U*-test *P* value=0.12), but since the presence of an episomal form of HPV is likely to be a prerequisite for integration, it is likely that the integrated versus episomal status is important, as a marker of oncogenicity levels that govern the length of time of neoplasias at their intraepithelial stages, before invasion develops. Oncogenicity could also influence the outcome of invasive carcinomas. We noticed in our case panel, that the rate of relapses was higher in tumours with a simple integration pattern, harbouring only two junctions than in cases with multiple viral insertions or with no insertions, developed in older women, an observation in line with the poor outcomes reported in tumours developed in younger women.^34^ To test the robustness of the above mechanistic/clinical analyses, and comprehensively explore the significance of the multi-parametric genomic and functional diversity of the HPV virus, extended series of cervical HPV-associated cancers examined as reported here are warranted.

Altogether, our data sheds light on several key aspects of HPV-associated tumours from biopsy and ct-DNA samples, allowing new perspectives in clinical oncology. Most importantly, the optimal molecular characterisation of the associated HPV junction sequences by Capture-NGS will provide in 1–2 weeks the specific DNA biomarkers for the patient biological follow-up, during and after treatment, an approach that has recently demonstrated its interest in the follow-up of patients with breast cancers.^35^ Collectively, the deep molecular analysis of viral-associated alterations nearby integration sites and genome-wide identification of candidate gene mutations should help the stratification of patients for innovative therapies in cervical cancer, targeting cellular genes^36,37^ or viral sequences.^38,39^ It should also be stressed that our approach allows the primary detection of any HPV-associated carcinoma using a simple blood test, an alternative method when detection of gynaecological cancers is difficult to implement. Another perspective will be to validate prospectively the potential value of this approach for the triage of cervical intraepithelial neoplasias at risk of progression. Finally, beyond cervical tumours, the present Capture-HPV approach is likely applicable to diagnose other HPV-associated cancers, such as head and neck carcinomas in which the proportion of HPV is increasing^40^ and is also applicable for detecting other viral-associated tumours or the movement of mobile elements in genomes.

## Materials and methods

### Cervical patient samples

DNA from 72 frozen biopsy cervical tissue samples were taken from patients diagnosed with SCC (squamous cell carcinomas), AC (adenocarcinomas) or AS (adenosquamous carcinomas). The patients were diagnosed in France, at the Institut Curie hospital in Paris (cases No. 1–3, 5–10, 12–15, 17, 18–19, 21–30, 32–46, 82–83, 87, 104, 114–116, 125, 128, 131–132, 138–144) or Saint Cloud (cases No. 4, 11, 16, 20, 31, 126) and the Centre François Baclesse in Caen (Cases No. 201–206, 208). The DNA samples were provided by the Biological Resource Center at the Institute Curie (AFNOR certification No. 2009/33837b). In accordance with French regulations, the patients were informed of the research performed on tissue specimens and did not express opposition. The FIGO stage and size of tumours were determined for each patient, with the average female age of 48 and with all tumour stages represented (Ib, II, IIa, IIb, IIIb, IVa).

### HPV probe design

Single-stranded biotinylated probes were designed with Roche NimbleGen (SeqCap EZ Developer targeting HPV, Roche NimbleGen Inc., Madison, WI, USA), in which over 22,000 individual and unique sequences, with an average probe length of 75 nucleotides, were synthesised to recognise 235 unique HPV types and variants, containing over 84 different low, medium, high-risk and even non-risk HPV’s. The list of sequences used for the NimbleGen SeqCap EZ design of these biotinylated probes is shown in Supplementary Table S1. They were designed to recognise the entire 7.8–7.9 kb HPV episomal genomic sequences, allowing for all possible breakpoints to be detected in the virus. A masking strategy was used to reduce biases towards a non-uniform design containing many copies of highly similar sequences. Several additional unique chromosomal coding regions were added to serve as internal controls to estimate the viral load by measuring the ratio of HPV reads per kb against these similarly captured genes. The probes were additionally screened to contain no significant homologies to the human (*hg19*) genome.

### Double capture of shotgun biopsy and circulating DNA libraries

Two hundred to 500 ng of DNA were used for each patient sample for subsequent DNA library preparation. The DNA libraries are prepared by nebulising, which shears the DNA mechanically and randomly with nitrogen, polishing the double-stranded DNA ends and T/A cloning of adapters, containing unique barcodes for each sample. This is followed by the Double Capture system, using the SeqCap EZ Rapid Library Small Target Capture method, developed by Roche. This is adapted to enrich for and capture small DNA targets, in which the DNA library samples are hybridised, two successive times, with the target HPV oligonucleotide probes. The expected technical advantage of this double capture method versus a single capture^20^ is to increase the specificity of the capture and enrich the fraction of relevant reads for subsequent bioinformatic analysis. DNA sequences captured by streptavidin were analysed using Roche 454 (GS Junior sequencing system) for patient cases 1–46, and Illumina MiSeq instruments, for the remaining 26 cases. Library samples were multiplexed, using one biotinylated HPV oligonucleotide probe set per 6 or 7 library samples. Single-end 454 shotgun reads were obtained, with an average length of about 500 nucleotides, allowing for easily identifiable viral-cellular junction reads, as well as purely viral reads, used for genotyping. The Illumina paired-end MiqSeq reads are 2×150 nt. Contig assemblies were also performed, using the Newbler software allowing for the full HPV integration status (the sequence of the viral insert with both 5′ and 3′ viral–cellular junctions). The sequencing parameters of each sample is provided in Supplementary Information.

The ct-DNA was prepared from 200 μl of serum collected from a patient blood sample at the time of diagnosis, using the QIAamp viral DNA (QIAGEN France SAS, Courtaboeuf, France) enrichment and blood kits. No sonication was performed before library preparation. The size profiles of the library DNA fragments were controlled on a Bioanalyzer before and after adaptor ligation. Fragments between 150 to 500 bps were purified and used for the subsequent Capture steps, performed as for the biopsy tumour samples described above.

### Bioinformatic pipeline for analysis of viral-cellular DNA junctions and HPV genotypes

Raw sequence data reads were obtained as either sff (binary) or fna (txt) formatted files. The sff files were converted into fastq files, using a python script: sff_extract 0.3.0 (http://bioinf.comav.upv.es/sff_extract/). All tools used thereafter were obtained from the Galaxy interface, at the Insitut Curie, whose development is based on a similar interface, available at (https://usegalaxy.org/) and is shown in Supplementary Figure S1.

We began by analysing the genotype of the sample. First, all of the sff reads were converted to fastq reads, that we aligned against a reference fasta file, containing 37 HPV low, medium and high-risk reference HPV genomes, obtained from the NIH PaVE website (http://pave.niaid.nih.gov/). Next, we used the Bowtie 2 tool from Galaxy set at default parameters, with an ‘end-to-end’ mode of global alignment. The alignment (SAM file) is visualised using Tablet (http://bioinf.scri.ac.uk/tablet/). In this manner, we identify the HPV type of the sample, as Tablet displays the number of reads that are aligned against each of the 37 different HPV reference viral genomes.

Next, the viral–cellular DNA junctions were analysed. Here again we aligned the reads using the Bowtie 2 tool, against the HPV subtype reference genome, previously identified. However, we run a local alignment in order to obtain the purely HPV reads and also the hybrid reads containing at least 28 nucleotides aligned on HPV (the default value of Bowtie 2). We remove PCR-duplicates using the ‘Mark Duplicate reads’ Picard tool on the BAM file, to obtain unique, yet overlapping reads. We then convert the BAM into SAM files and filter all unaligned reads (using SAM tools).

The alignment with Tablet easily reveals simple (two clear junctions, 2J) from multiple viral insertion cases. For such 2J cases, the alignment of reads against the reference viral strain, allows the identification of non-pure HPV reads, or viral–host junctions, that do not entirely align against the viral reference genome. This alignment also shows the portion of the viral genome that is deleted upon integration. The CIGAR code is used to confirm the alignment ends of each NGS read at precise positions that contain an additional sequence, not aligned against HPV ('S' flag). In this manner, we obtain the non HPV portion of these reads that align against the *hg19* reference using the *blat* tool (http://genome.ucsc.edu), to identify the precise insertion site of the virus in the human genome.

Alternatively, if a deleted or gapped region in the Tablet alignment is not detected, then we implement a filter to remove all purely viral reads and to highlight only viral–host junction reads. For this, we performed two local read alignments: the first against the HPV reference and the second against the *hg19* reference sequence. After removing PCR duplicates and unaligned reads, the Galaxy tool, ‘Compare Two Data Sets’, is used and allows comparison between two data sets. Thus, by comparing both SAM files (HPV and Human) we find the common read names that are partially aligned on both HPV and human sequences, that we call hybrid reads. As before, we visualise the SAM files in Tablet, with clearly visible viral-host breakpoints.

### Assignment of viral-cellular sequences and breakpoint coordinates

The coordinates of the HPV-genomic breakpoints were assigned using the *hg19* reference genome and HPV Pave website, for reference HPV genomes. Fifty- to 55-nt sequences overlapping the viral–cellular breakpoints were aligned, resulting in 5 up to 24 nts of gapped sequence identity either at or surrounding the breakpoint, within the entirely aligned sequence (Supplementary Figure S2). Exact overlapping sequences at the breakpoint, were assigned to the human genome.

### *De novo* read assembly (Newbler)

We performed a *de novo* assembly, when possible, using the Newbler software from Roche. The raw data (sff of fastq file) was used as input and the following default parameters were used as following: minimum read length 20; minimum overlap length 40; minimum overlap identity; 90%.

The output fna file, containing all of the contig sequences were aligned against the HPV genome. Then, the non-HPV portion of the contig was identified by aligning on *hg19* (with the BLAT tool). This allowed us to confirm junctions that we identified previously, from single reads and confirm our integration breakpoints obtained from our single reads. Indeed, for most of the 2J cases, Newbler was able to assemble one or two long contigs, encompassing both the 5′ and 3′ flanking viral–human breakpoints, as well as the entire integrated viral sequence.

### PCR validation of viral–host junctions

The DIPS-PCR (Detection of integrated papillomavirus sequences) method, followed by Sanger sequencing was performed on all samples, to validate our NGS identified junctions using the Capture-HPV-NGS method, as described previously.^25,26^

### Measurement of viral loads

HPV viral loads were determined in two ways. First, as previously described,^8^ by quantitative PCR, in which the HPV E7 gene is quantified relative to the reference PSA gene (2 copies of DNA per cell) using quantitative PCR (Sybr Green PCR Core Reagents). The viral charge is obtained using the formula CV=2^−^^ΔCt^×2. Differently, the HPV viral load was measured from the capture NGS results, by counting and comparing the nucleotide coverage of the HPV sequences and those of the three control house-keeping genes, included in the same tube as the HPV capture probes. These three unique genes (*GAPDH*, *KLK3* and *RAB7A*) with a size (6–11 kb each) comparable to the HPV were captured with a total of ~3,000 copy-number control probes. The estimated viral load was determined by the ratio of HPV versus control gene read coverage, normalised per nucleotide. Correlation between these methods is shown in Supplementary Information.

### Genome-wide analysis of HPV integration using CytoScan Affymetrix

All samples (except the #45 and 46 cases, with no detectable HPV DNA) were analysed at the Genetic platform at the Institut Curie, using the Genome-Wide Human CytoScan HD Array from Affymetrix, for comprehensive whole-genome coverage of DNA from cervical biopsy samples (includes 2.67 million markers for copy-number analysis, with 750,000 SNP probes and 1.9 million non-polymorphic probes). Analysis was performed using the Chromosome Analysis Suite software (ChAS) version 3.0 (Affymetrix), as well as the Genome Alteration Print (GAP) software, developed at the Institut Curie.^41^ Frequency of Amplicon, Gain and Loss (FrAGL) was performed on the bioinformatics platform (U900, INSERM) of the Institut Curie.

## Figures and Tables

**Table 1:** Clinical and genomic status of 72 cervical carcinoma cases

*Clinical Status*				*Genomic Capture/HPV NGS Status*					*Gene annotation*	*CGH array*
*Case* No.	*Age*	*Clinical stage*	*Tumour size*	*HPV type*	*HPV status*	*Chromosomal locus (# Jxs)*	*Breakpoint 1*	*Breakpoint 2*	*Genes disrupted or nearby (within 500 *kb*)*	*FDA profiles*
1	33	Ib	28	16	2J-COL	13q14.2 (2)	48,591,279	48,993,666	***NUDT15, MED4, ITM2B, RB1, LPAR6,** SUCLA2*	DEL
2	55	Ib	40	16	2J-COL	Xp22.2 (2)	13,772,242	13,778,432	***OFD1,**RAB9A,EGFL6,GPM6B*	DEL
3	38	Ib	50	I8	2J-COL	1q44 (2)	244,237,555	244,434,007	*ZBTB18, AKT3, C1ORF100*	DEL
4	48	IIa	78	18	2J-COL	15q22.31 (2)	66,166,361	66,166,368	***RAB11A,** MEGF11*	FLAT
5	34	IIb	60	16	2J-COL	4q21.3 (2)	87,230,060	87,581,814	***MAPK10, PTPN13,** ARHGAP24, AFF1*	DEL
6	44	IIb	65	51	2J-COL	12q24.22 (2)	116,887,495	116,887,498	*MED13L, RNFT2*	FLAT
7	54	IIIb	60	16	2J-COL	20q11.22 (2)	32,361,289	32,371,046	***ZNF341,** PXMP4, NECAB3, CBFA2T2*	DEL
8	73	IVa	50	33	2J-COL	19q13.33 (2)	49,994,844	49,994,845	***RPL13A,** FLT3LG*	FLAT
126	48	Ib1	22	45	2J-COL	1p36.22 (2)	12,631,949	12,634,760	***DHRS3***	FLAT
208	61	IIb	30	68	2J-COL	6p21.2 (2)	37,200,791	37,200,794	***TBC1D22B***	FLAT/AMP
17	40	NA	NA	18	2J-COL	4p16.1 (2)	8,626,438 ***rr***	8,638,831	*CPZ, GPR78, TRMT44, ACOX3, HMX1*	FLAT
9	34	Ib	17	45	2J-NL	2q11.2 (2)	100,265,612	100,330,086	***AFF3,** REV1*	4× AMP
10	34	Ib	25	18	2J-NL	13q22.1 (2)	73,830,702	74,152,642	*KLF5, KLF12, PIBF1*	1.5× AMP
11	47	Ib	27	18	2J-NL	6q21 (2)	113,335,611	113,390,769	—	4× AMP
12	45	II	NA	18	2J-NL	8q24.21 (2)	128,198,367	128,315,709	*PNCR1, CCAT1, CCAT2, POU5F1B, MYC*	1.5× AMP
13	47	IIb	30	18	2J-NL	13q22.1 (2)	73,917,347	74,048,469	*KLF5, KLF12, PIBF1*	4× AMP
14	43	IIb	40	18	2J-NL	4q13.3 (2)	74,685,790	74,722,519	***CXCL6, PF4V1,** IL8, CXCL1, RASSF6, PF4*	1.5×AMP
15	54	IIb	40	18	2J-NL	11q22.2 (2)	102,715,466	102,741,318	***MMP12,** MMP3*	FLAT
16	45	IIb	55	73	2J-NL	2q22.3 (2)	146,422,046	146,461,927	—	1.5× AMP
18	65	IIb	60	73	2J-NL	7q31.1 (2)	110,336,697	110,343,623	***IMMP2L***	1.5× AMP
19	42	IIb	70	18	2J-NL	7p21.1 (2)	17,352,218	17,531,668	***AHR***	2× AMP
20	48	IVb	50	18	2J-NL	14q32.31 (2)	102,632,970	102,646,250	***WDR20***	1.5× AMP
82	65	Ib1	28	18	2J-NL	17q23.1 (2)	57,918,925	57,951,794	***TUBD1***	2× AMP
87	30	II	55	16	2J-NL	18q21.33 (2)	59,573,260	59,657,033	*RNF152, PIGN*	2× AMP
205	32	IIb	56	16	2J-NL	4p14 (2)	38,216,299	38,229,928	*TBC1D1*	2× AMP
143	62	Ib2	52	18	2J-NL	3q28 (2)	189,620,030	189,647,619	*TP63, LEPREL1*	2× AMP
139	36	IIb	31	16	2J-NL	3q28 (2)	189,012,737	189,049,506	***TPRG1***	FLAT
125	52	Ib1	21	18	2J-NL	9q21.13 (2)	75,675,668	75,787,011	***ALDHA1A,** ANXA1*	2× AMP
21	33	Ib	NA	18	MJ-CL	4p15.33 (5)	11,447,692	11,658,906	*HS3ST1*	3×AMP
22	49	Ib	40	16	MJ-CL	3q29 (5)	193,756,403	193,893,583	*HES1*	2× AMP
23	42	Ib	70	16	MJ-CL	2q22.3 (3)	146,417,585	146,460,099	—	1.5× AMP
24	77	IIb	NA	31	MJ-CL	3q27.3 (6)	187,600,502	187,635,420	*SST, RTP2, BCL6, LPP*	2× AMP
25	68	IIb	36	16	MJ-CL	17q23.1 (3)	57,920,534	57,921,079	*VMP1*	FLAT
26	54	IIb	49	16	MJ-CL	9p11 .2 (5)	45,456,749 ***rr***	45,470,782 ***rr***	***LOC100132167***	FLAT
27	40	IIb	50	18	MJ-CL	7p12.3 (3)	46,344,871	46,485,689	—	FLAT
28	33	III	43	16	MJ-CL	5q12.3 (3)	66,390,695	66,547,382	***MAST4***	3.5× AMP
29	43	IIIb	40	16	MJ-CL	8q24.21 (9)	128,676,026	128,760,118	*MYC*	4× AMP
115	45	IIb	55	16	MJ-CL	19p13.2 (3)	13,166, 672	13,167,744	***NFIX***	4× AMP
116	28	Ib1	30	18	MJ-CL	8q24.21 (3)	128,226,807	128,248,795	***CCAT1, POU5F1B***	4× AMP
144	57	IVa	NA	16	MJ-CL	9q34.11 (5)	132,324,282	132,368,299	*NTMT1, C9orf50*	2× AMP
30	31	Ib	19	31	MJ-SC	20q13.32 (2)	56,885,148	56,888,374	***RAB22A***	1.5× AMP
						Xq12 (2)	67,757,289	67,757,299	*YIPF6*	1.5× AMP
31	46	Ib	30	16	MJ-SC	2q33.9 (1)	205,291,450		*PARD3B*	FLAT
						6q22.32 (2)	126,892,874	126,892,879	*CENPW*	FLAT
						9q22.32 (2)	97,769,459	97,769,475	***C9ORF3***	FLAT
						13q22.1 (1)	74,189,850		*KLF12, PIBF1*	FLAT
						20p12.1 (2)	15,399,977	15,430,574	***MACROD2***	1.5× AMP
32	47	Ib	39	16	MJ-SC	1p31.1 (1)	71,855,150		*NEGRI, ZRANB2, PTGER3*	3–4× AMP
						1q31.1 (3)	189,550,418	189,571,920	*_*	2.7× AMP
						2p15 (2)	63,994,580	64,014,068	*UGP2, MDH1, VPS54, WDPCP*	1.5× AMP
						8q24.23 (2)	138,711,393	138,712,591	*FAM135B*	FLAT
						13q21.33 (1)	72,386,277		***DACH1***	FLAT
						15q12 (1)	26,064,619		***ATP10A***	FLAT
						15q21.1 (2)	44,885,327	45,326,422	***SPG11, SORD***	1.3× AMP
33	49	IIb	40	16	MJ-SC	1q42.3 (3)	236,291,878	236,367,459	***GPR137B,**NID1,ERO1LB, LYST,EDARADD*	2.3× AMP
						20q11.21 (1)	30,208,967		*HM13,ID1,COX412,BCL2L1*	FLAT/AMP
34	81	IIIb	80	16	MJ-SC	2q34 (2)	210,398,973	210,398,978	***MAP2***	1.5× AMP
						2q36.3 (2)	228,020,329	228,020,331	***COL4A4***	4× AMP
						7q11.23 (2)	72,586,506	74,849,215 ***rr***	***GATSL1, GATSL2***	2× AMP
						8q23.1 (1)	107,273,933		*ZFPM2, OXR1*	1.7× AMP
142	64	IIIb	62	68	MJ-SC	19q13.42 (1)	56,187,255		***EPN1***	2× AMP
						15q23 (2)	70,475,675	70,544,526	*TLE3, UACA*	2× AMP
201	52	Ib	NA	16	MJ-SC	Xp22.2 (6)	13,367,991	13,390,802	***ATXN3L, EGFL6***	2× AMP
						7q21.11 (1)	78,604,568		***MAGI2***	2× AMP
203	71	IVa	NA	16	MJ-SC	8q24.23 (2)	137,221,054	137,222,823	*_*	FLAT
						1q32.2 (2)	209,517,782	209,574,020	*LAMB3, HSP11B1*	2× AMP
35	34	Ib	70	16	EPI	No junctions	NA	NA	NA	ND
36	65	IIb	20	33	EPI	No junctions	NA	NA	NA	ND
37	53	IIb	40	16	EPI	No junctions	NA	NA	NA	ND
38	68	IIb	40	16	EPI	No junctions	NA	NA	NA	ND
39	54	IIb	50	16	EPI	No junctions	NA	NA	NA	ND
40	68	IIb	50	16	EPI	No junctions	NA	NA	NA	ND
41	55	IIb	60	16	EPI	No junctions	NA	NA	NA	ND
42	63	IIb	80	52	EPI	No junctions	NA	NA	NA	ND
43	57	IV	NA	16	EPI	No junctions	NA	NA	NA	ND
44	44	IV	60	73	EPI	No junctions	NA	NA	NA	ND
114	60	IIa	51	16	EPI	No junctions	NA	NA	NA	ND
128	43	IIb	30	16	EPI	No junctions	NA	NA	NA	ND
131	31	Ib1	24	16	EPI	No junctions	NA	NA	NA	ND
140	44	IIa	45	16	EPI	No junctions	NA	NA	NA	ND
141	87	IIa	20	42	EPI	No junctions	NA	NA	NA	ND
104	60	IIIb	48	31	EPI	No junctions	NA	NA	NA	ND
204	74	IIa	40	31	EPI	No junctions	NA	NA	NA	ND
206	60	IIb	21	6	EPI	No junctions	NA	NA	NA	ND
132	36	Ib2	25	16	EPI	No junctions	NA	NA	NA	ND
138	29	Ib1	25	16	EPI	No junctions	NA	NA	NA	ND
45	31	Ib	60	—	NEG	—	—	—	—	—
46	54	IIb	35	—	NEG	—	—	—	—	—

Abbreviations: EPI, episomal HPV genomes; HPV, human papillomavirus; NA, not available; ND, not determined; NEG, negative for HPV; *rr*, repetitive region; 2J-COL, two hybrid chromosomal–HPV junctions whose orientations are co-linear; 2J-NL, two hybrid chromosomal–HPV junctions whose orientations are non-linear.

CGH array: flat/normal, deletion or amplification (FDA) structural profiles based on comparative genomic hybridisation (CGH) microarray analyses. Bold entries represent directly disrupted genes.

## Notes

Supplementary Table S1 List 235 HPV Sequences

Supplementary Table S2 Full Clinical NGS data

Supplementary Figure S1 Pipeline

Supplementary Figure S2 Breakpoints

Supplementary Figure S3 ct-DNA Analysis

Supplementary Figure S4 Correlation between viral and HPV status

Supplementary Figure S5 2J-COL CGH

Supplementary Figure S6 2J-NL CGH

Supplementary Figure S7 FrAGL

Supplementary Figure S8 MJ-CL CGH

Supplementary Figure S9 MJ-SC CGH

Supplementary Figure S10 Distribution HPV insertion sites

Supplementary Figure S11 Target genes

Supplementary Figure S12 HPV divergence

Supplementary Figure S13 HPV16 variants

Supplementary Figure S14 Age distributions
