# Supplementary material for: Mechanistic signatures of HPV insertions in cervical carcinomas
Source: NPJ Genom Med. 2016 Mar 16;1:16004–. doi: 10.1038/npjgenmed.2016.4 (PMC5685317; doi:10.1038/npjgenmed.2016.4)
Supplement: Supplementary Table S2 Full Clinical NGS data [file npjgenmed20164-s2.doc]

**Table S2**

**Table S2** **(continued)**
