# Supplementary figures and images for: Mechanistic signatures of HPV insertions in cervical carcinomas
Source: NPJ Genom Med. 2016 Mar 16;1:16004–. doi: 10.1038/npjgenmed.2016.4 (PMC5685317; doi:10.1038/npjgenmed.2016.4)

Fig S1

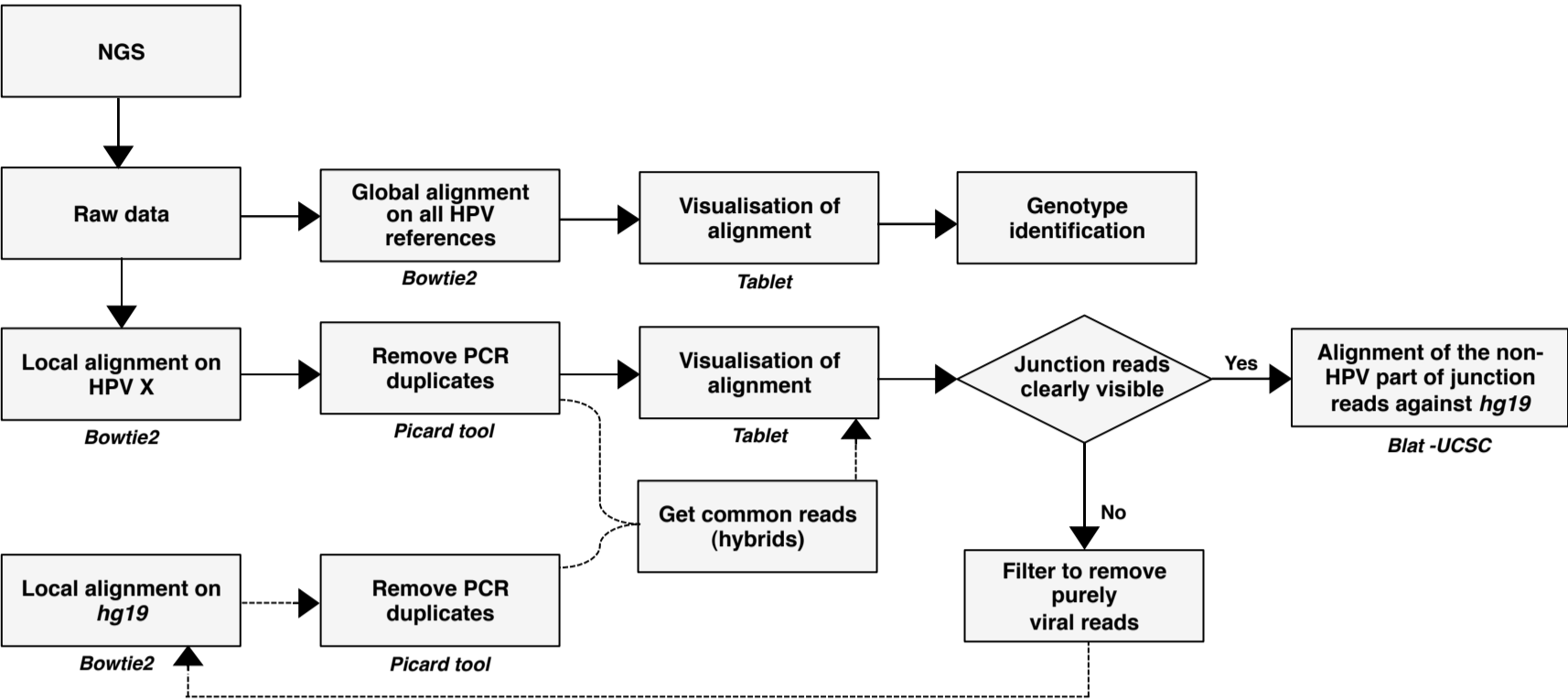

Supplement: Supplementary Figure S1 Pipeline [file npjgenmed20164-s3.pdf]

Fig S3

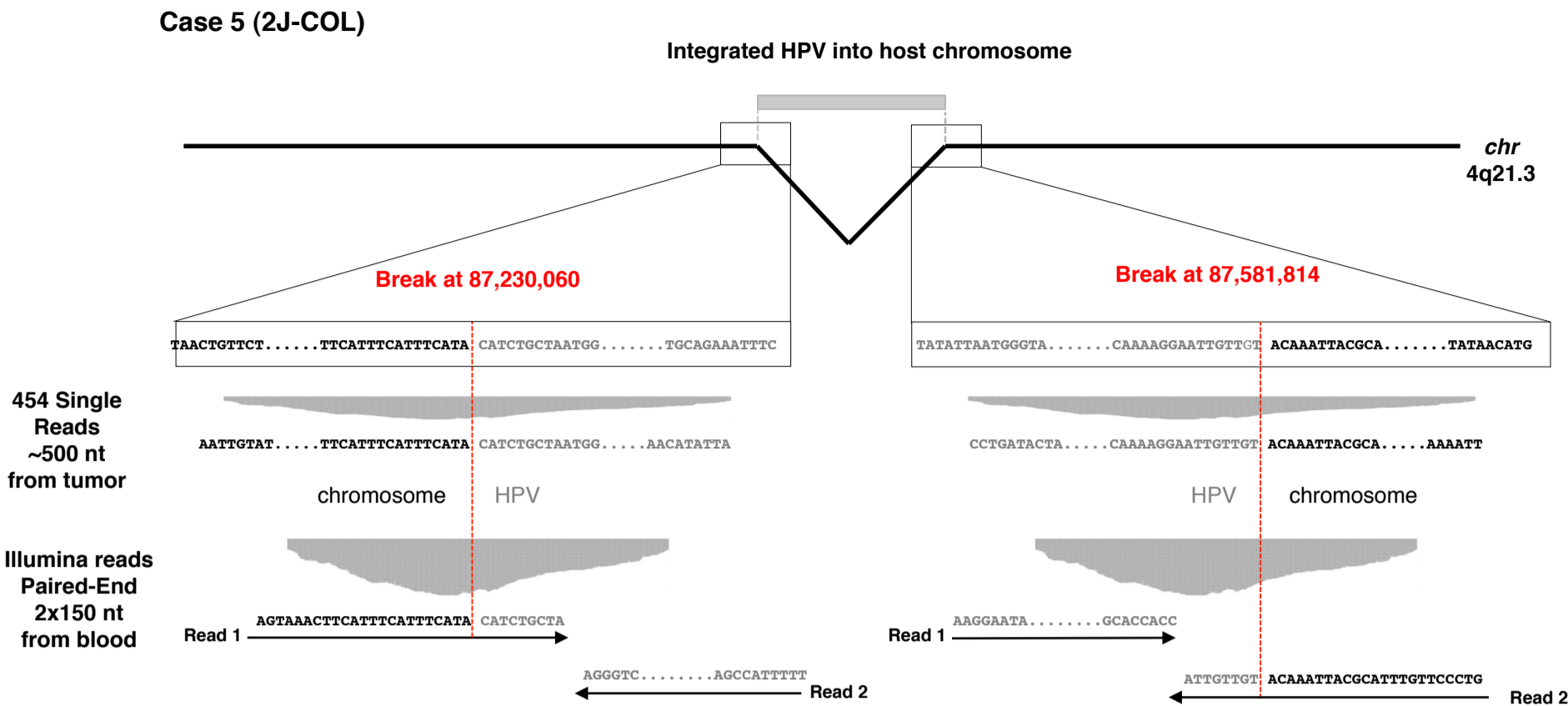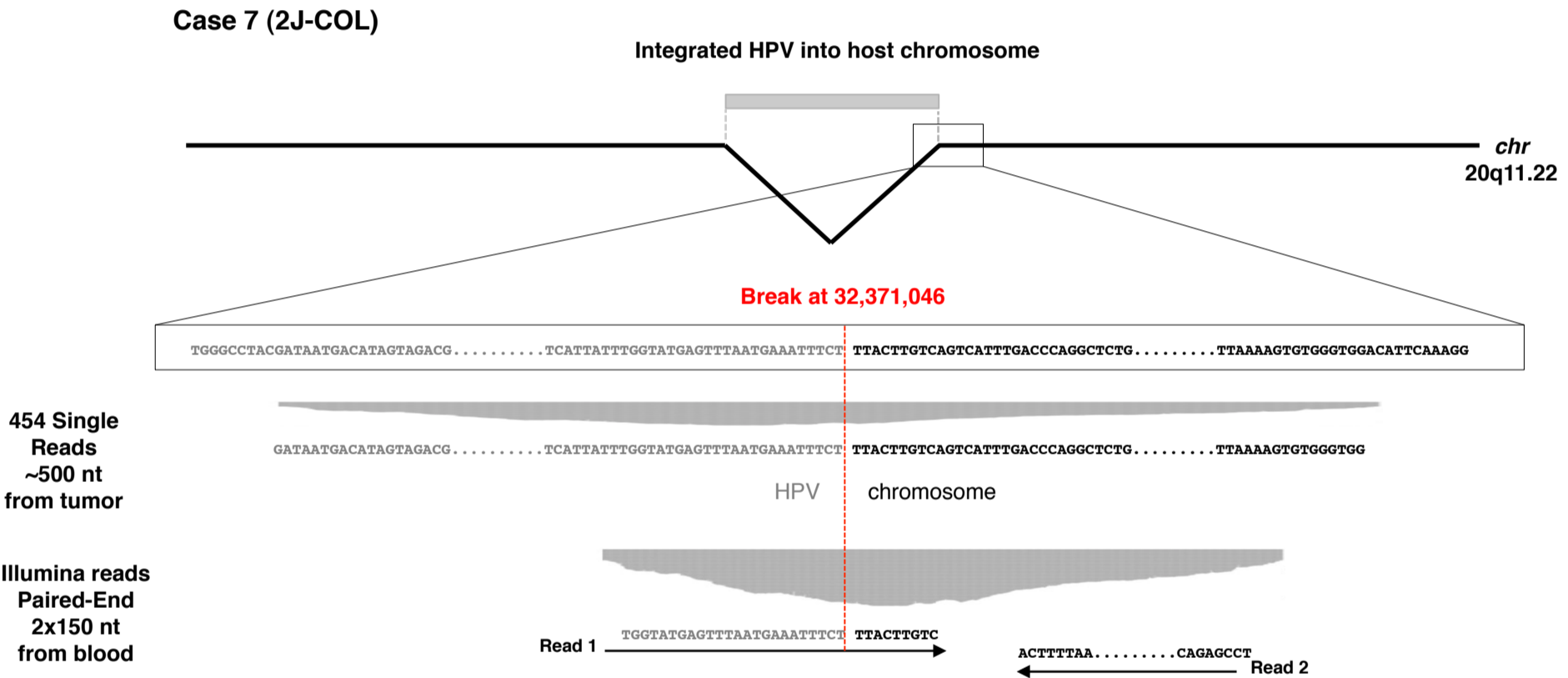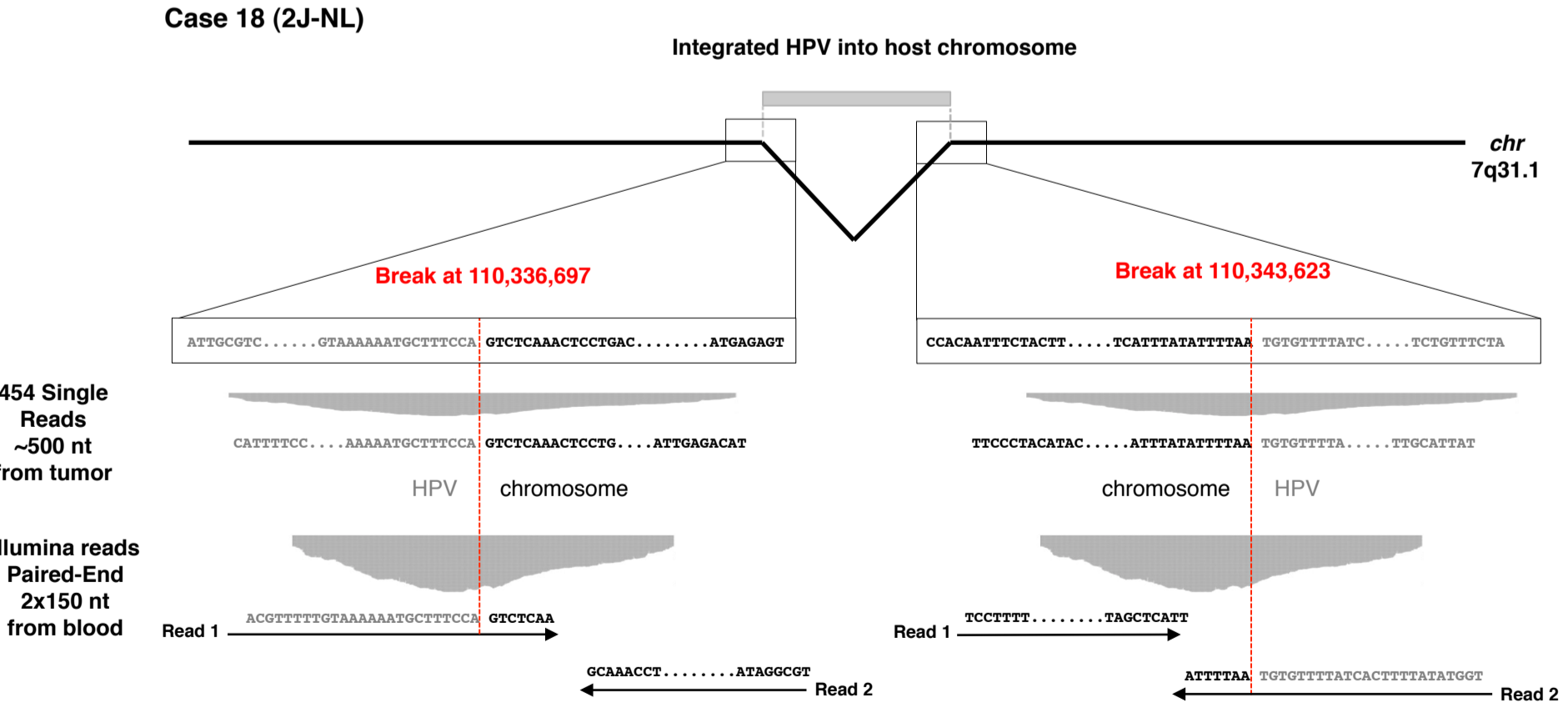

Fig S3

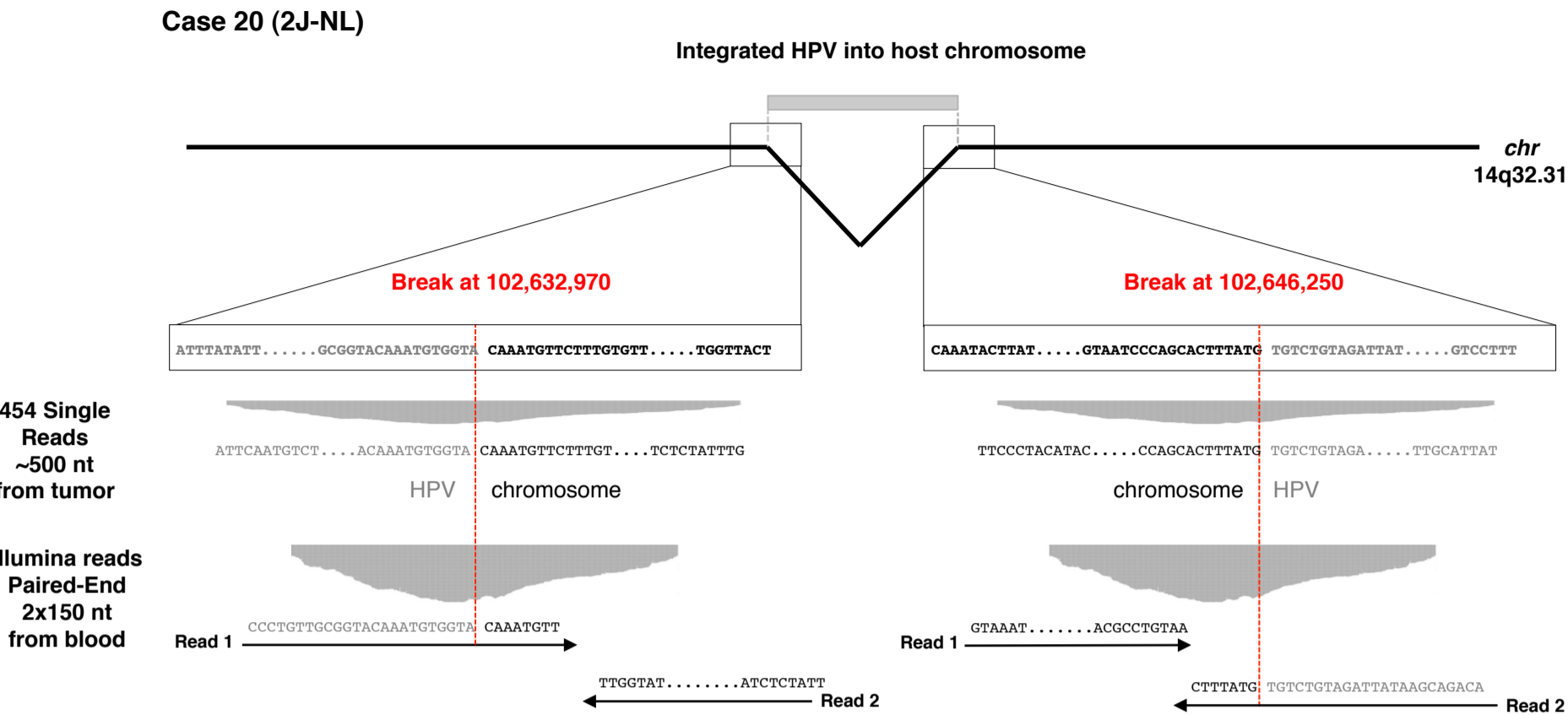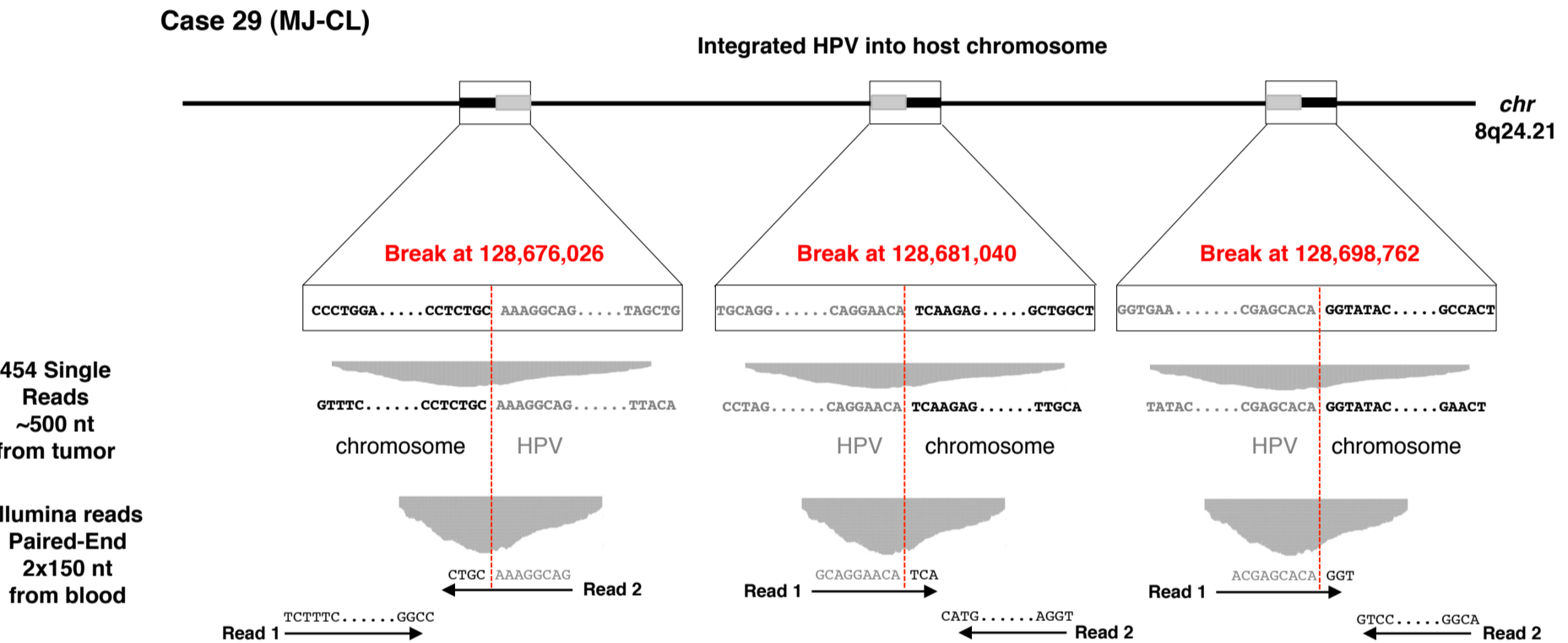

Supplement: Supplementary Figure S3 ct-DNA Analysis [file npjgenmed20164-s5.pdf]

Fig S4

A

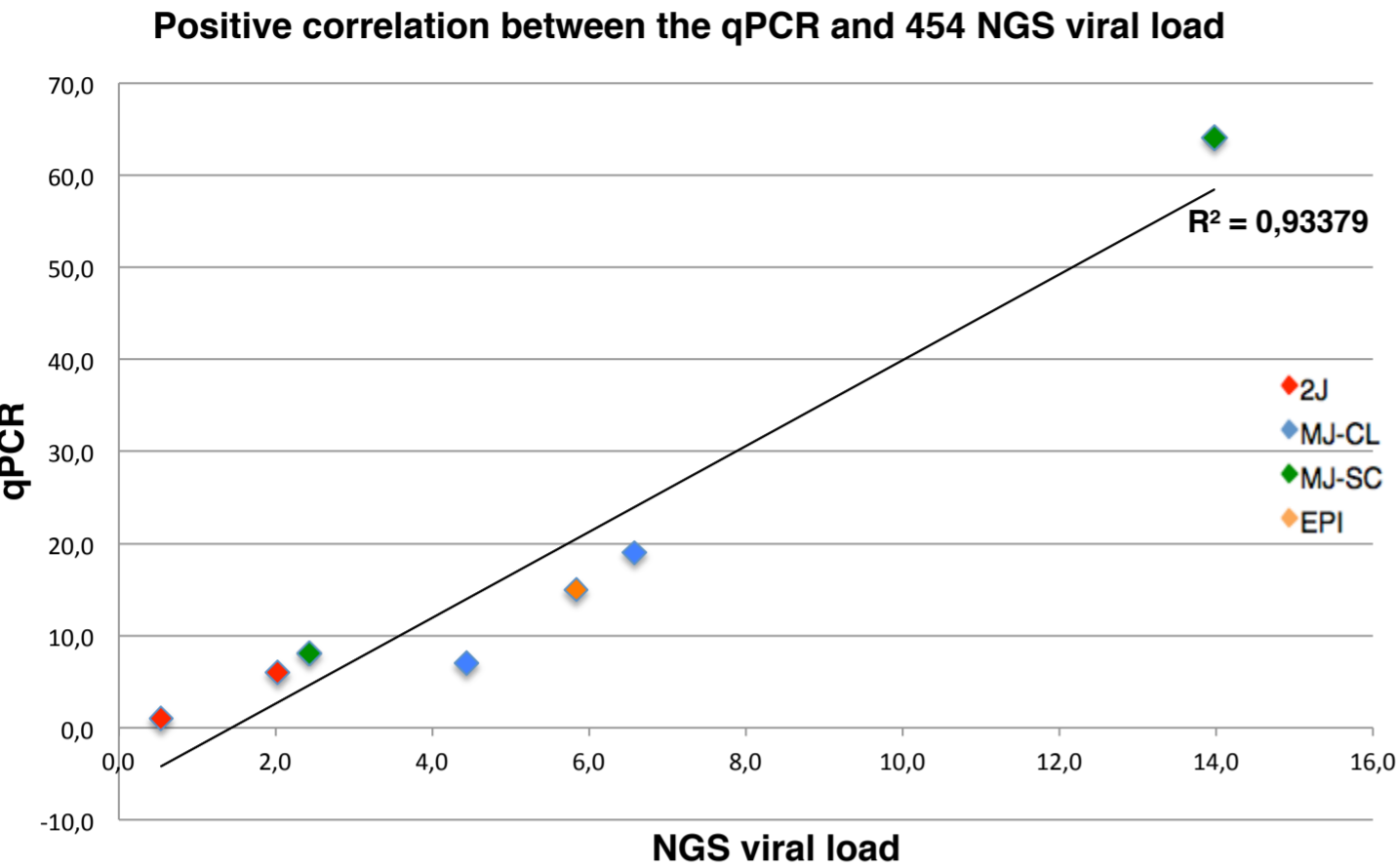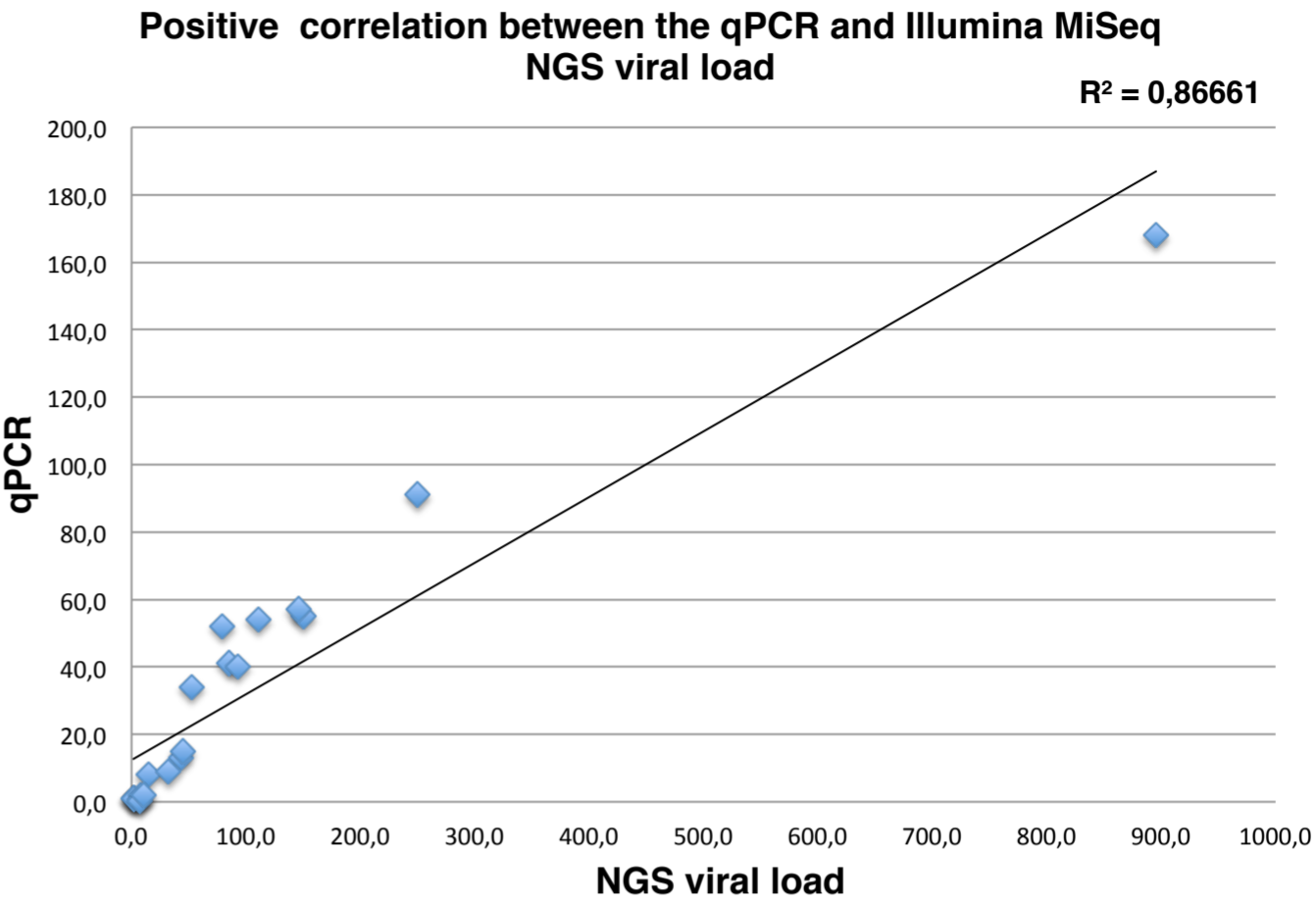

B

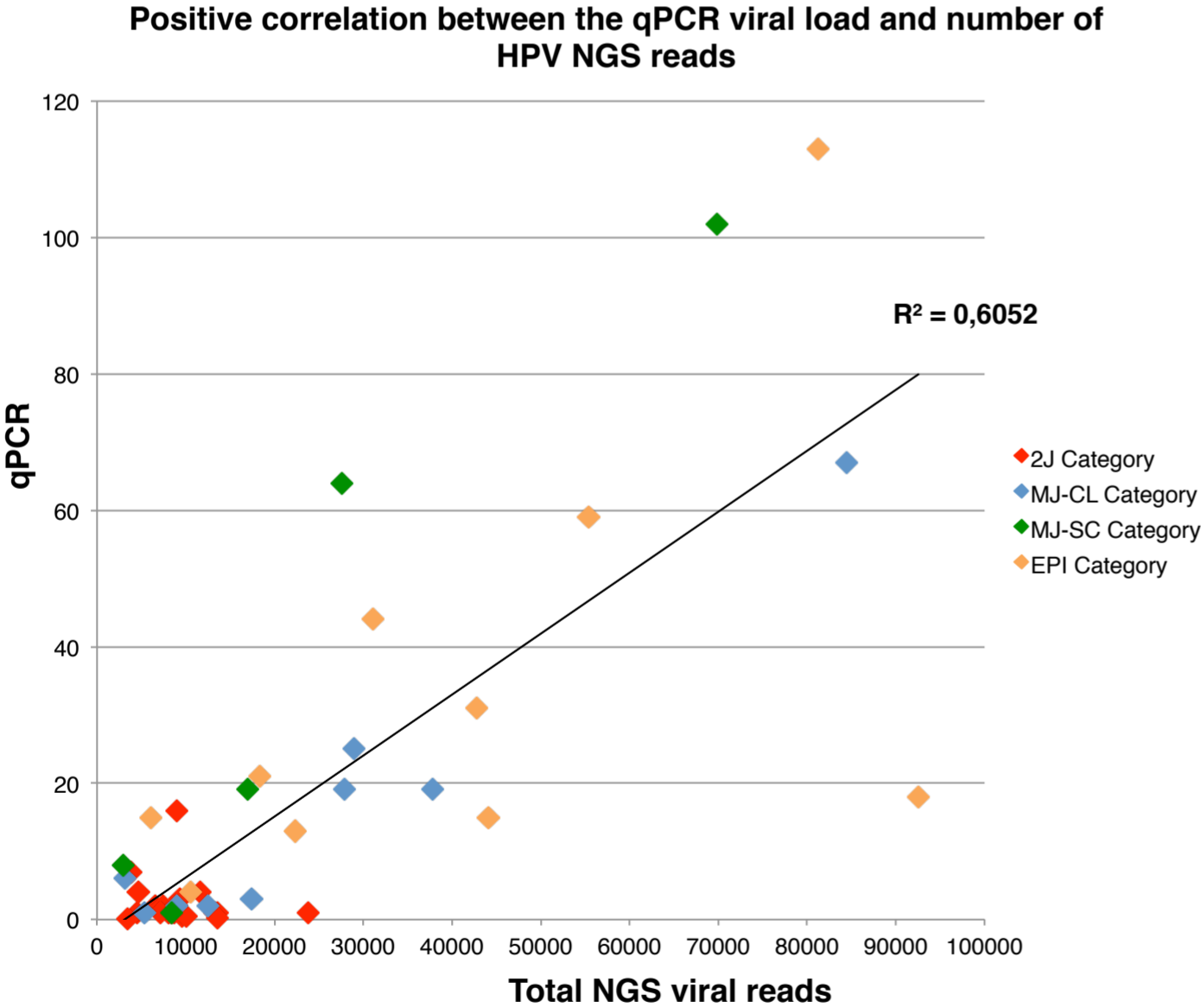

Supplement: Supplementary Figure S4 Correlation between viral and HPV status [file npjgenmed20164-s6.pdf]

Fig S5

Case 6 (2J-COL)

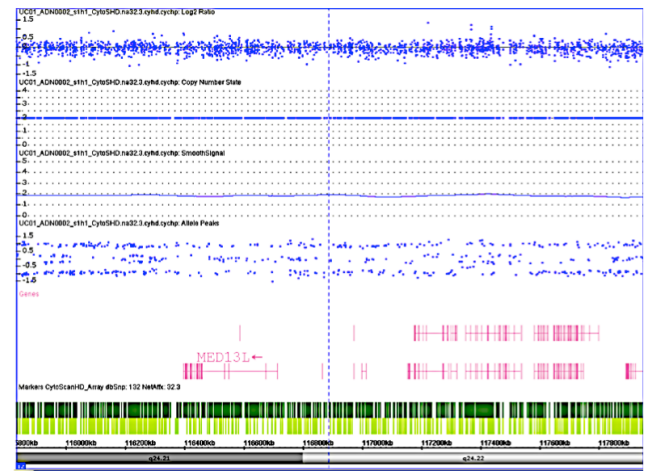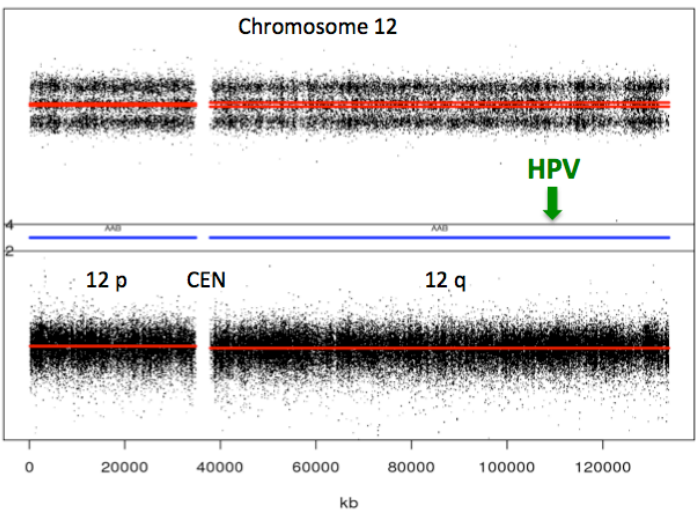

Case 7 (2J-COL)

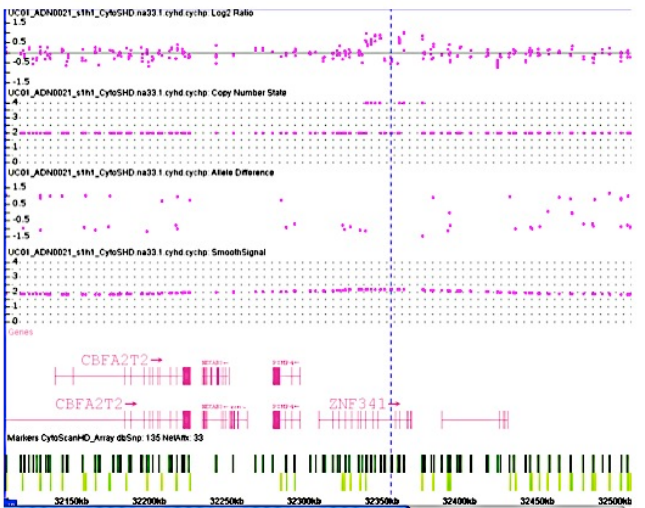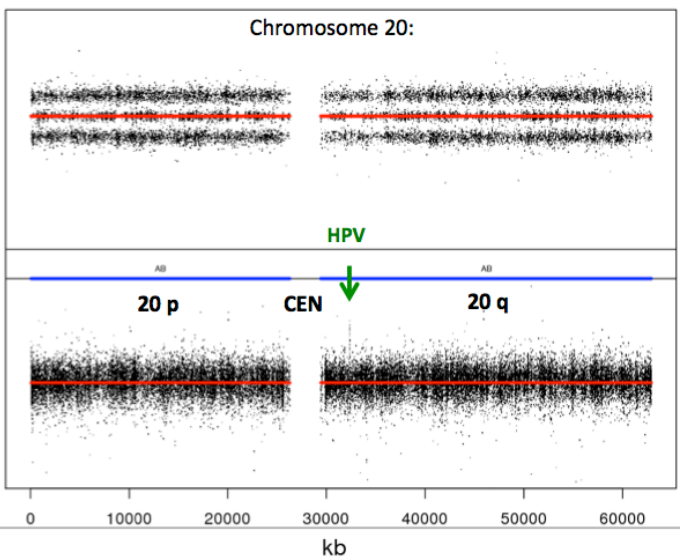

Case 8 (2J-COL)

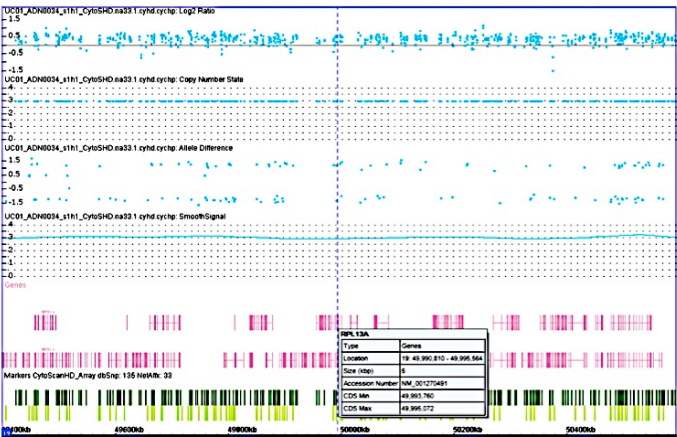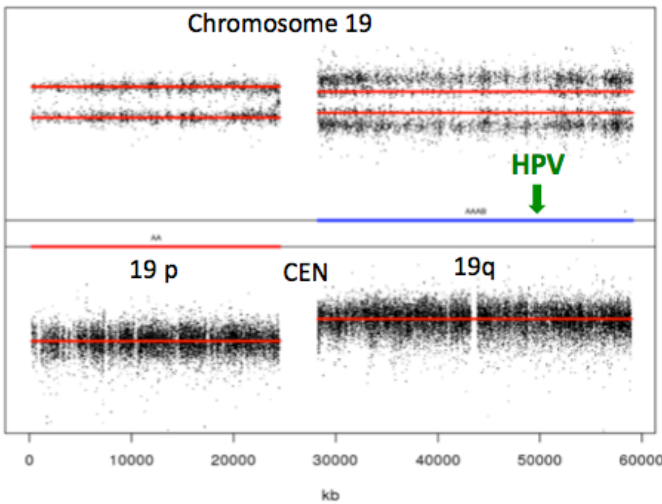

Fig S5

Case 126 (2J-COL)

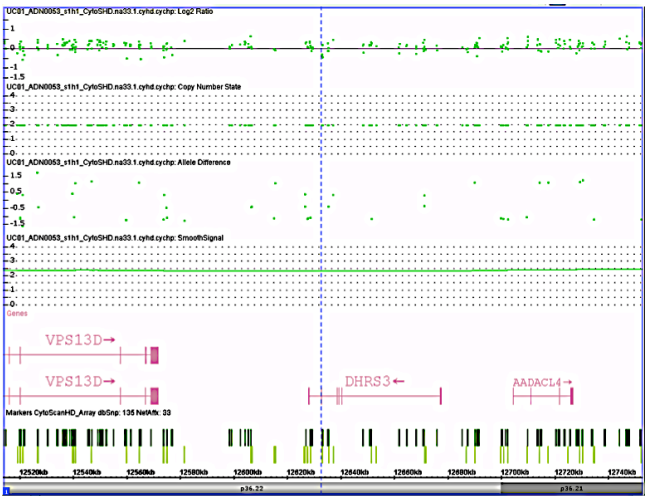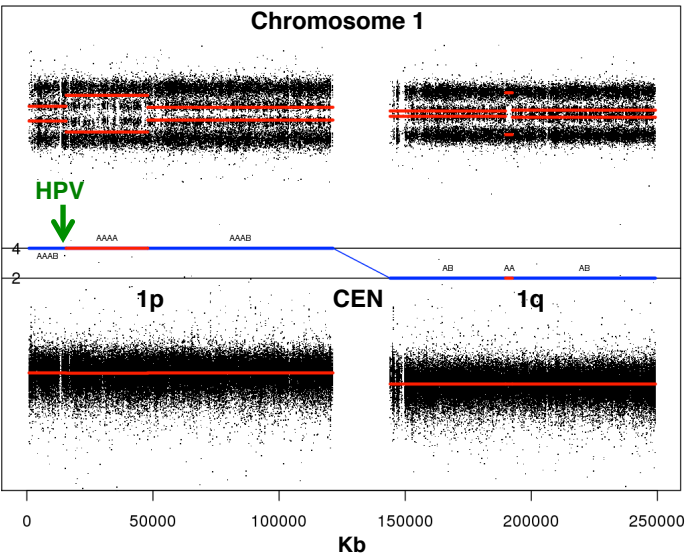

Case 208 (2J-COL)

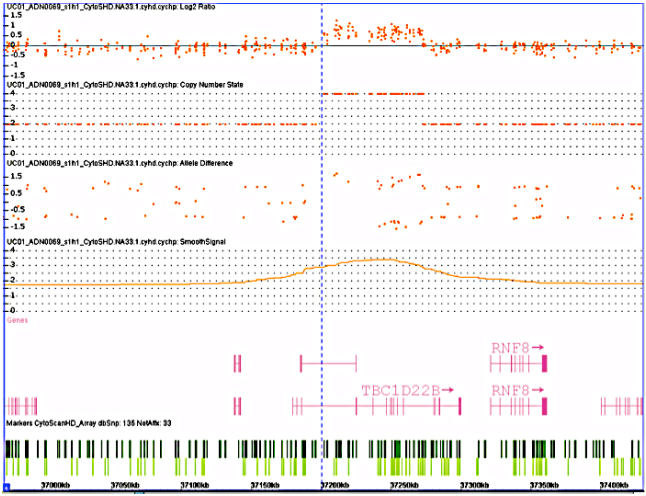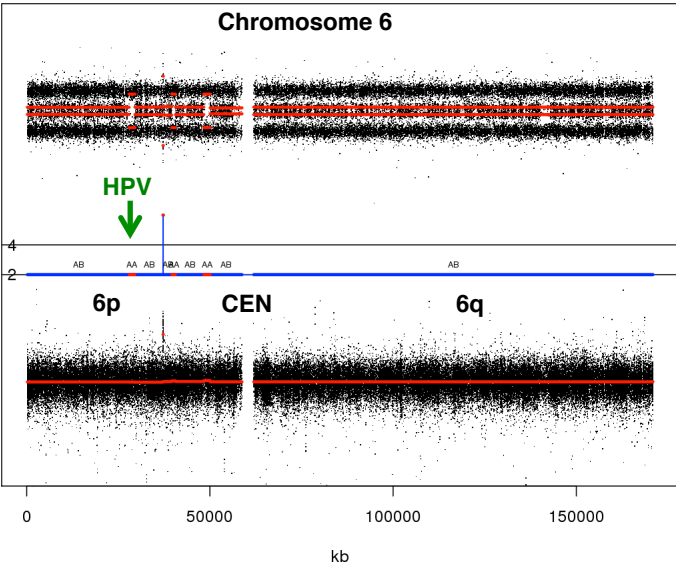

## Case 1 (2J-COL)

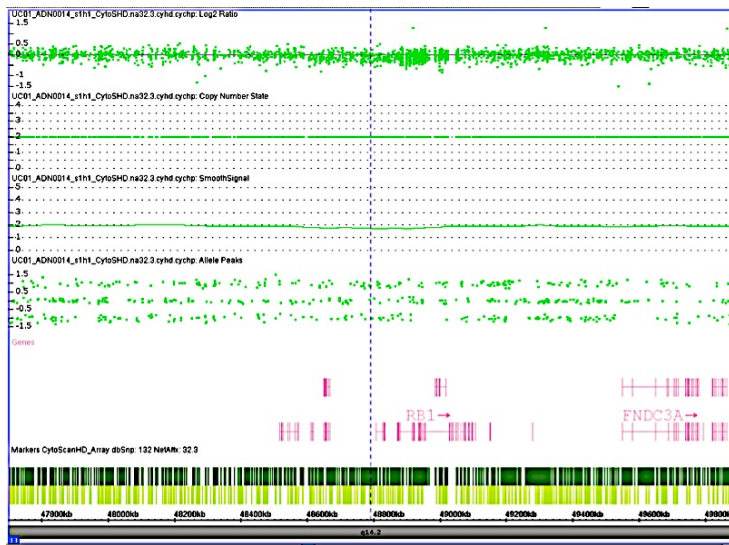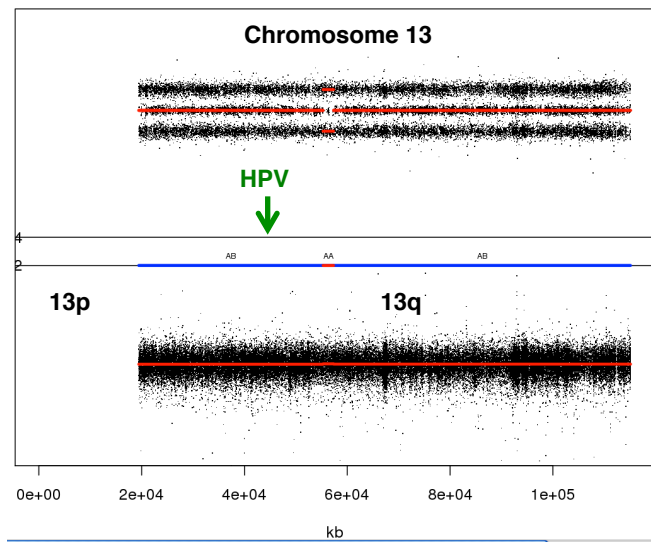

## Case 4 (2J-COL)

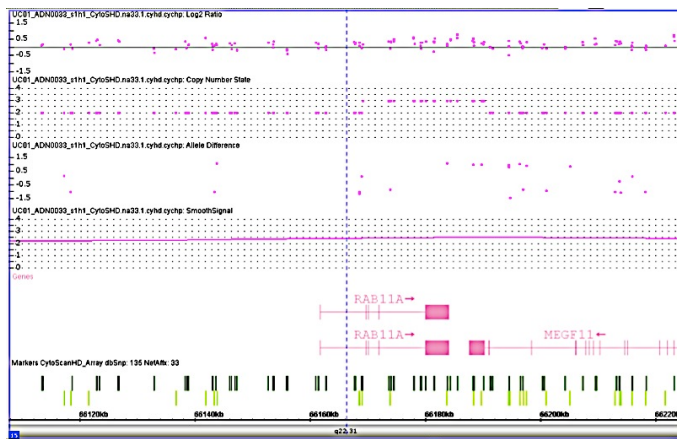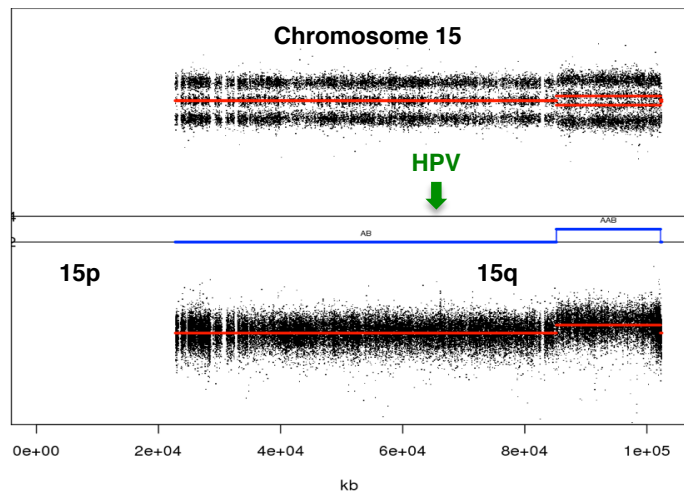

## Case 17 (2J-COL)

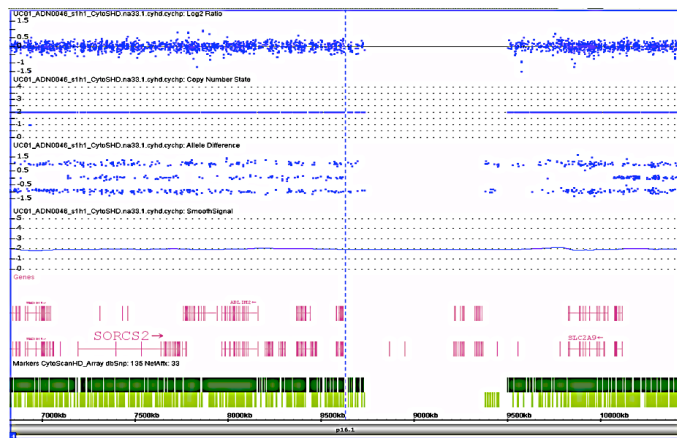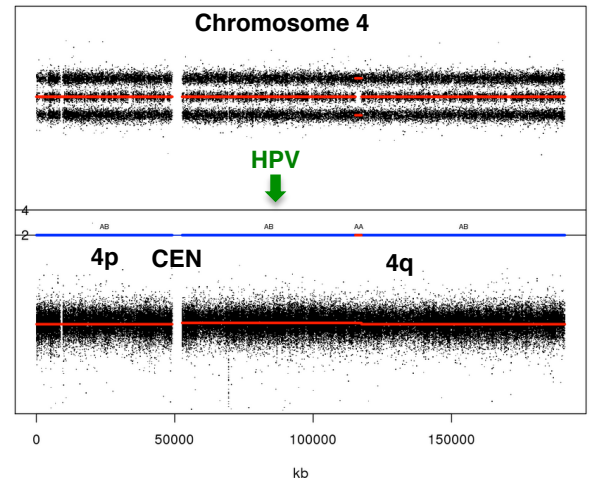

Supplement: Supplementary Figure S5 2J-COL CGH [file npjgenmed20164-s7.pdf]

Fig S7

2J-COL

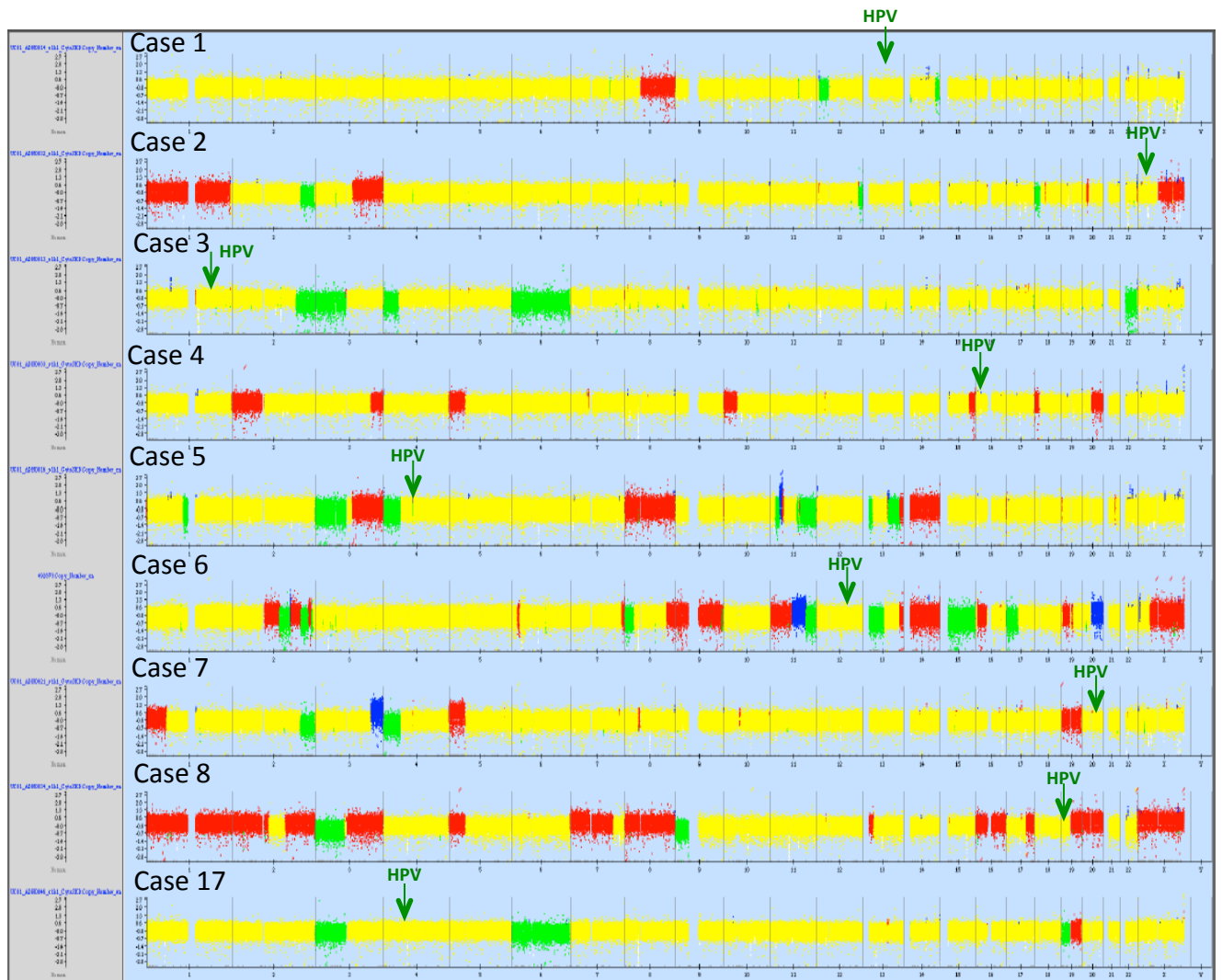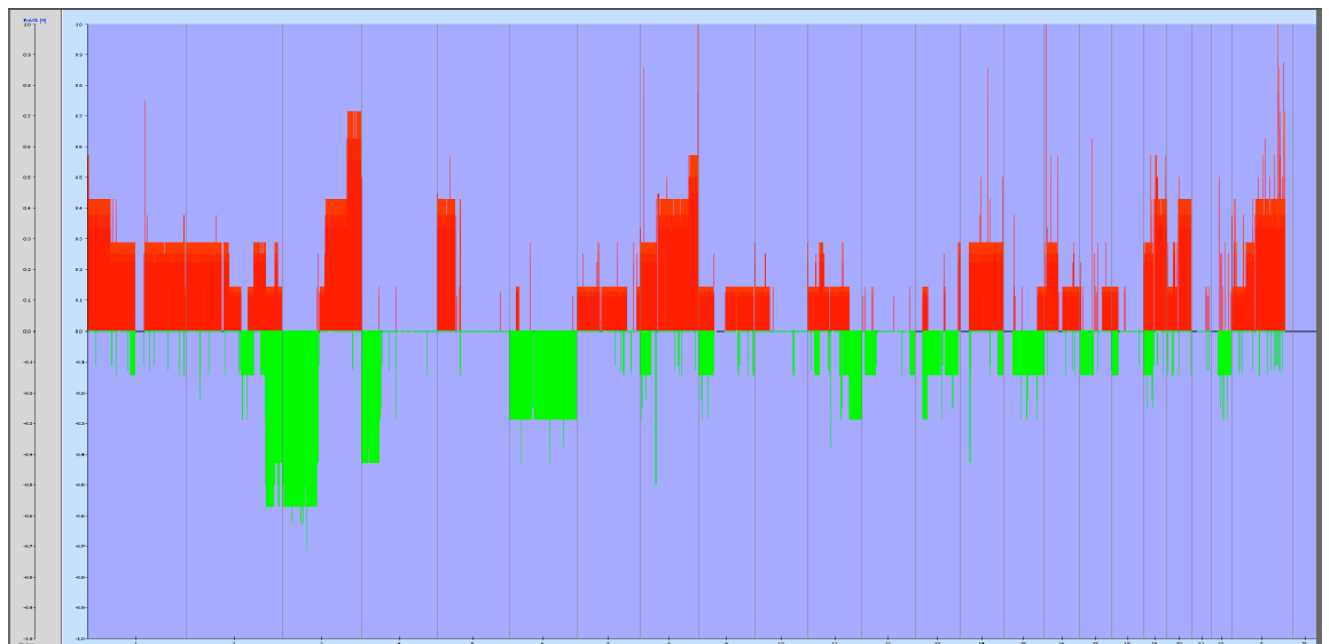

Fig S7

2J-NL

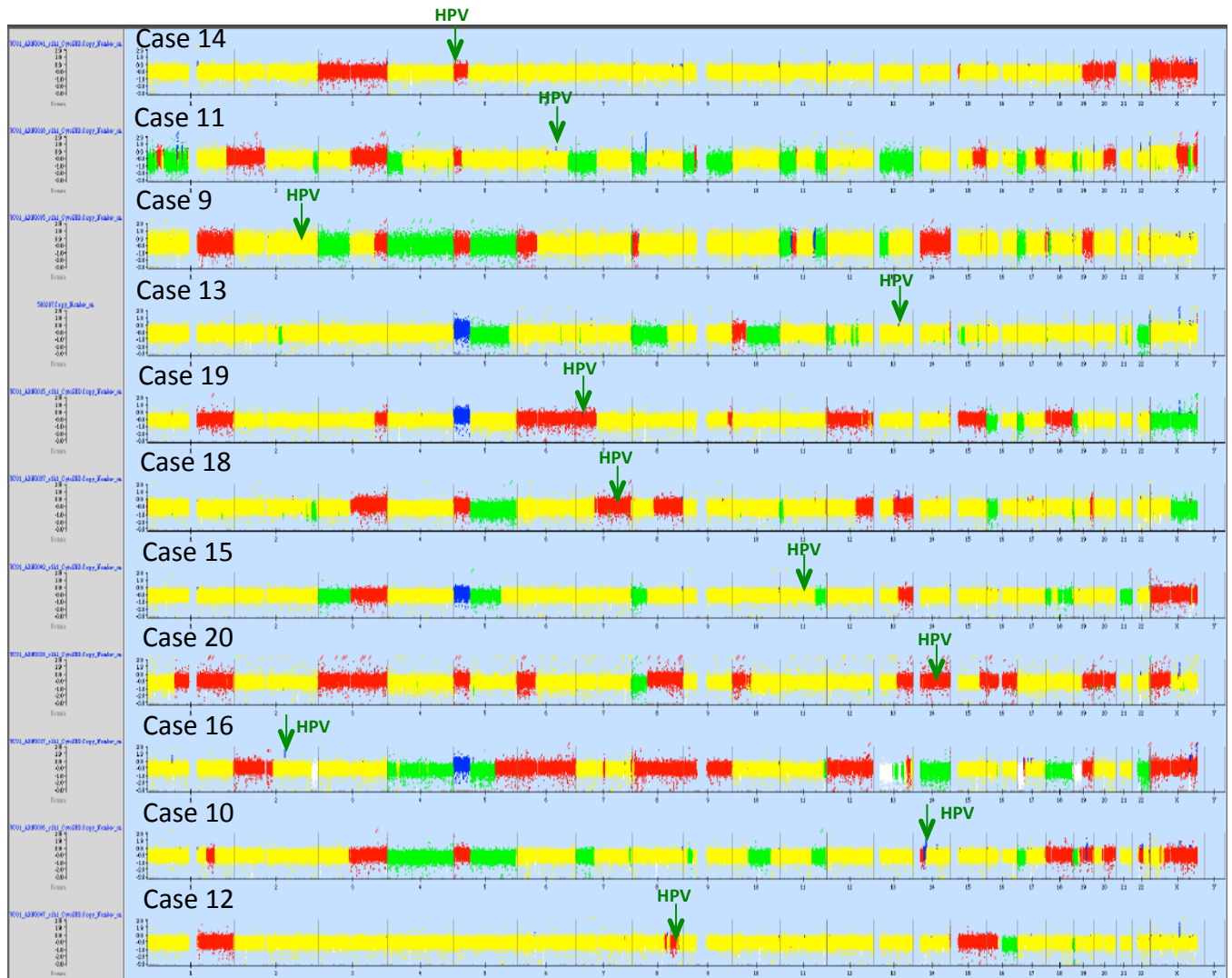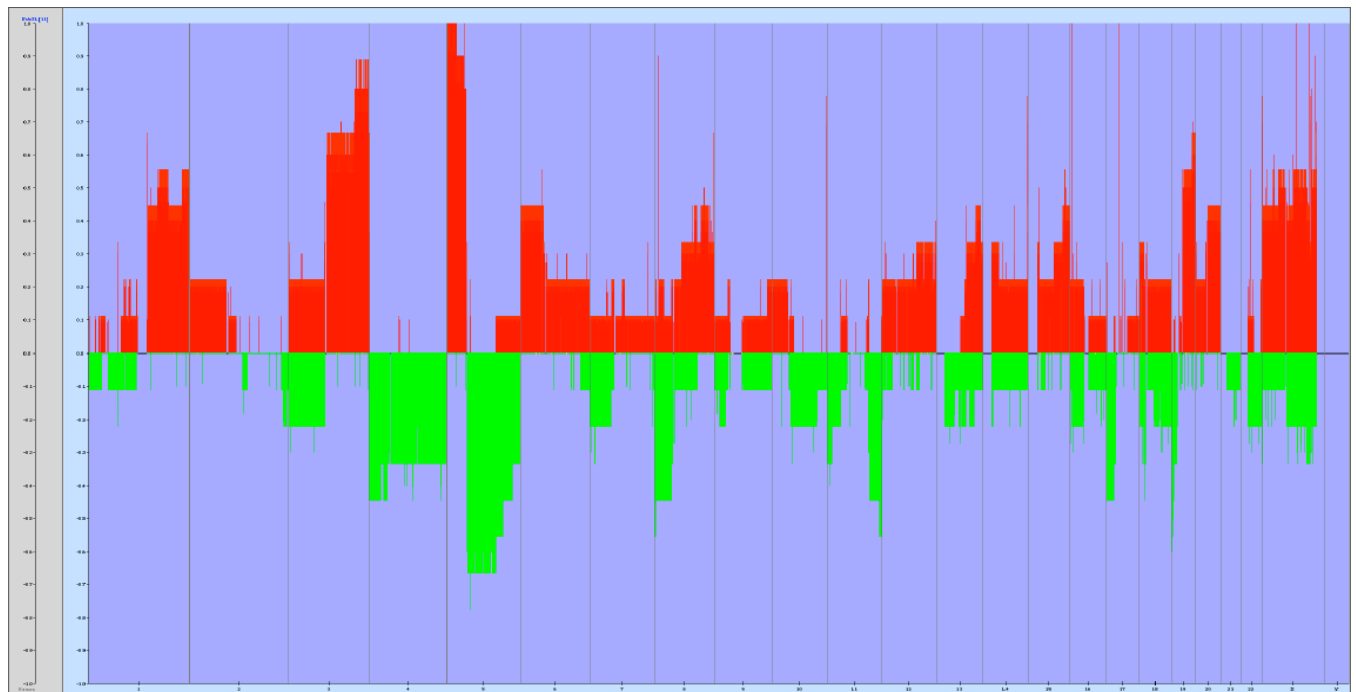

Fig S7

MJ-CL

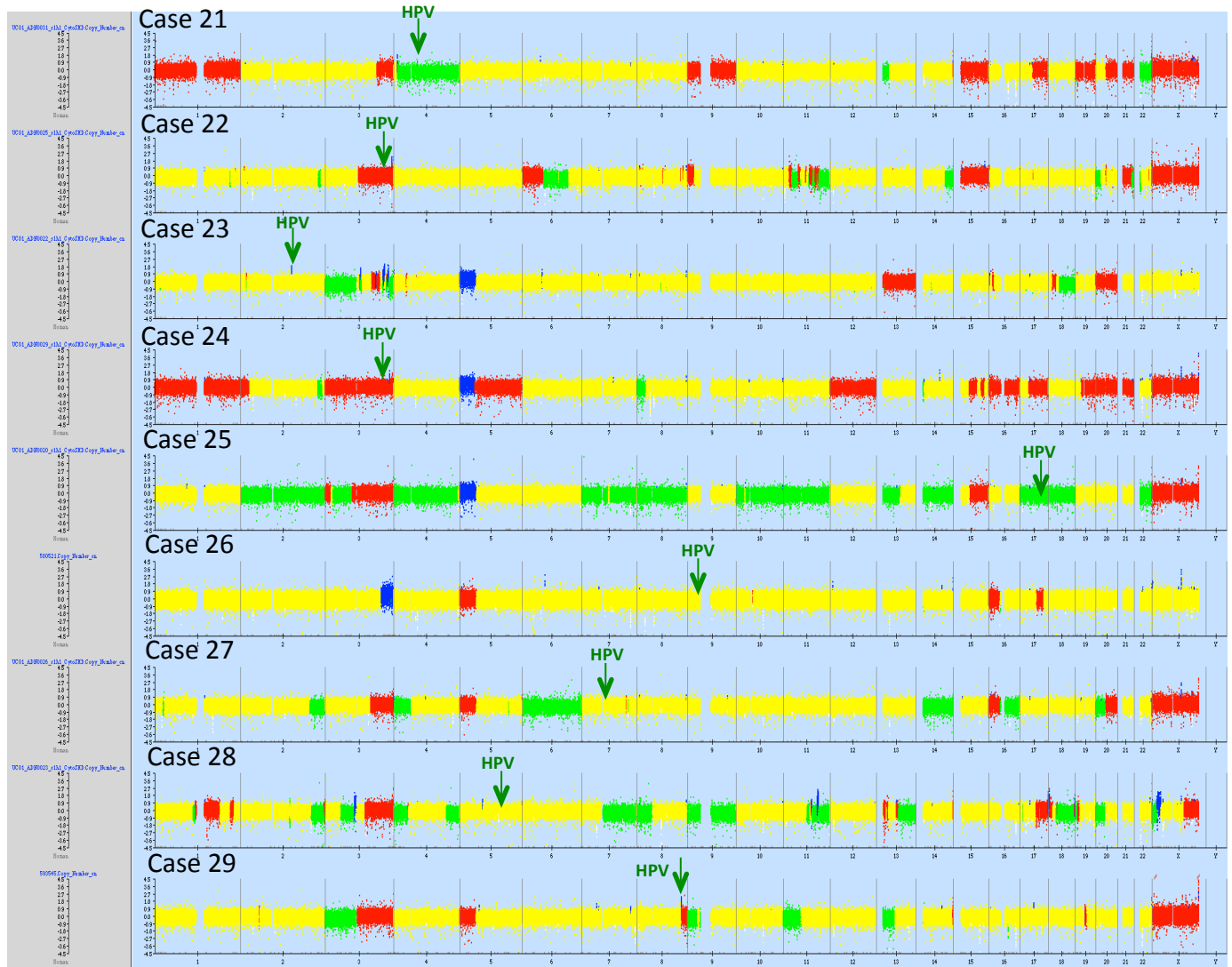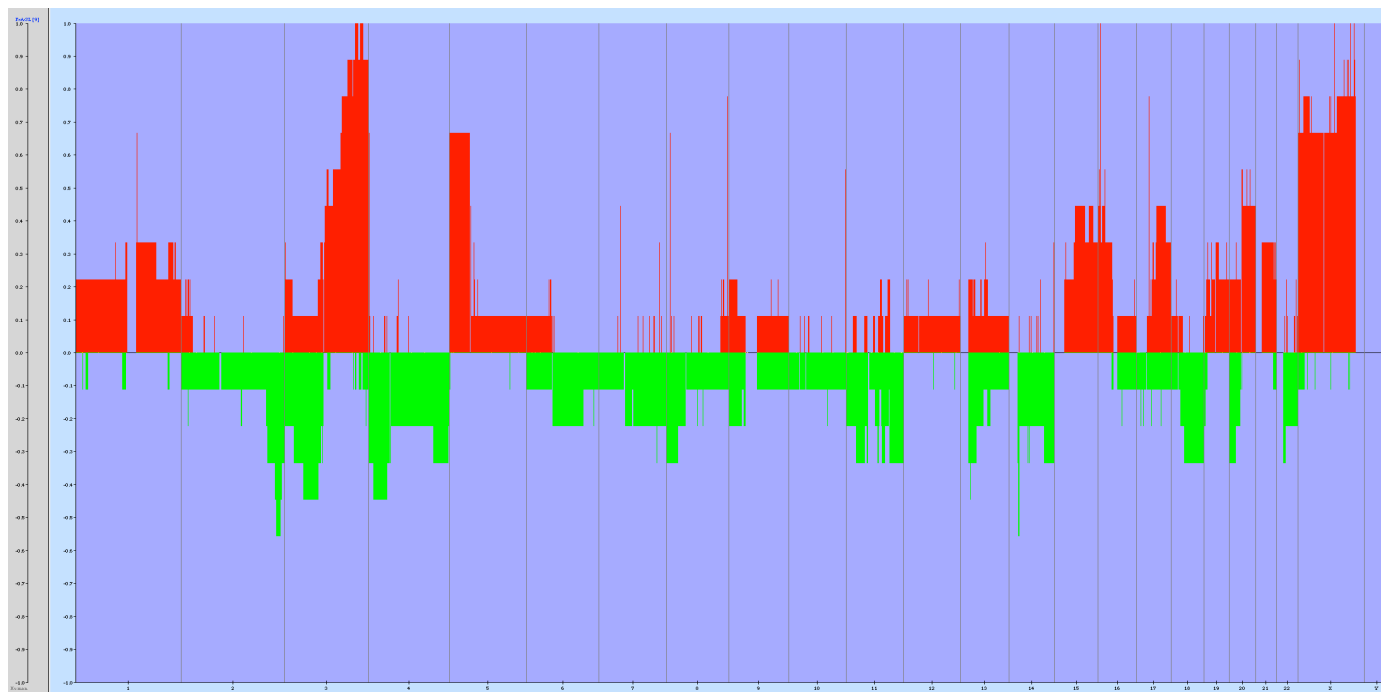

Fig S7

MJ-SC

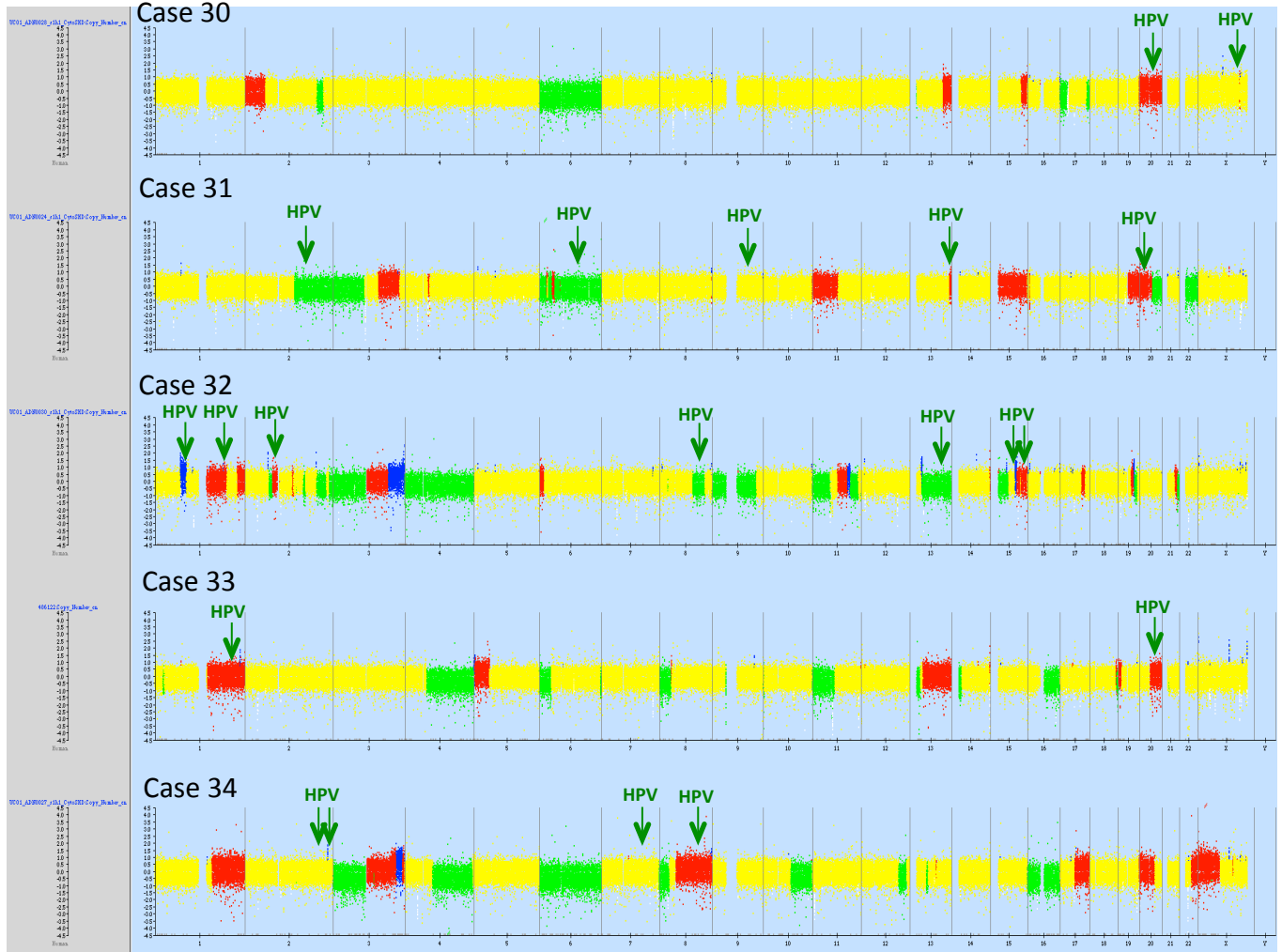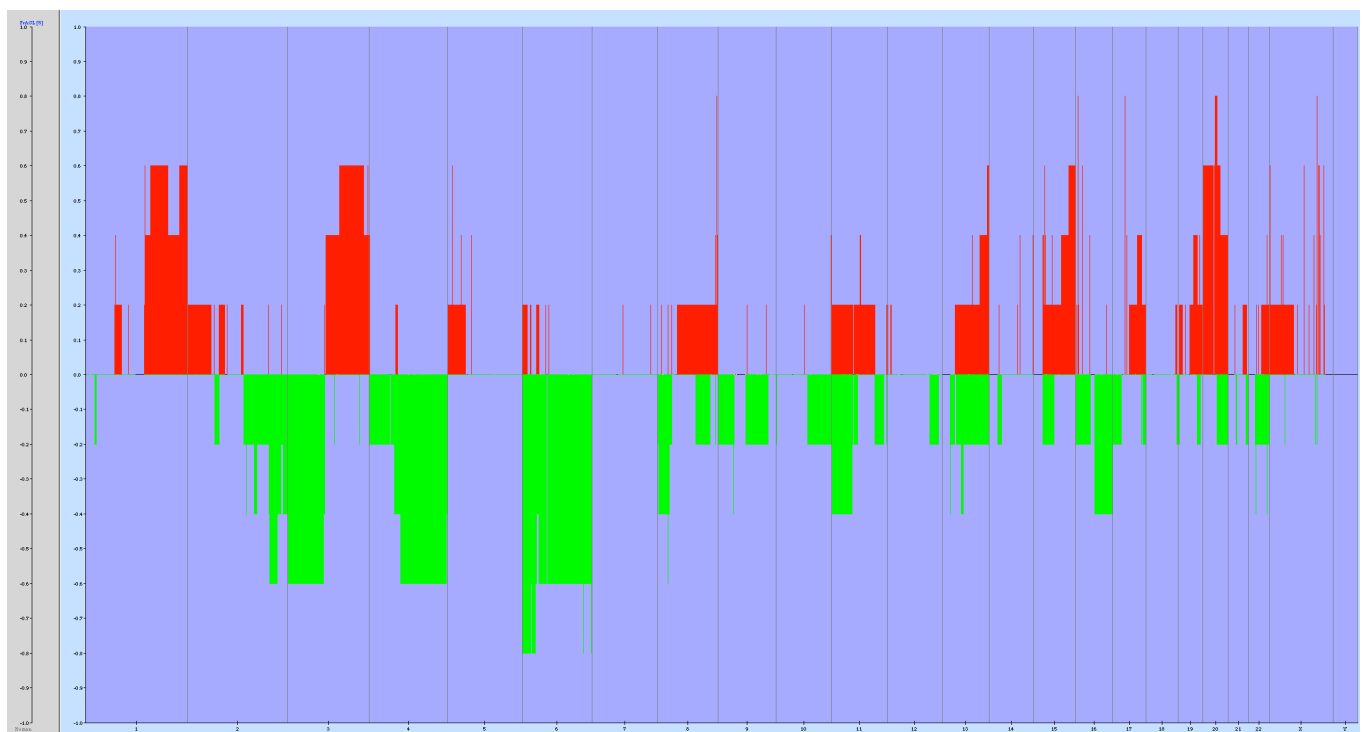

Fig S7

# EPI (no viral integration sites)

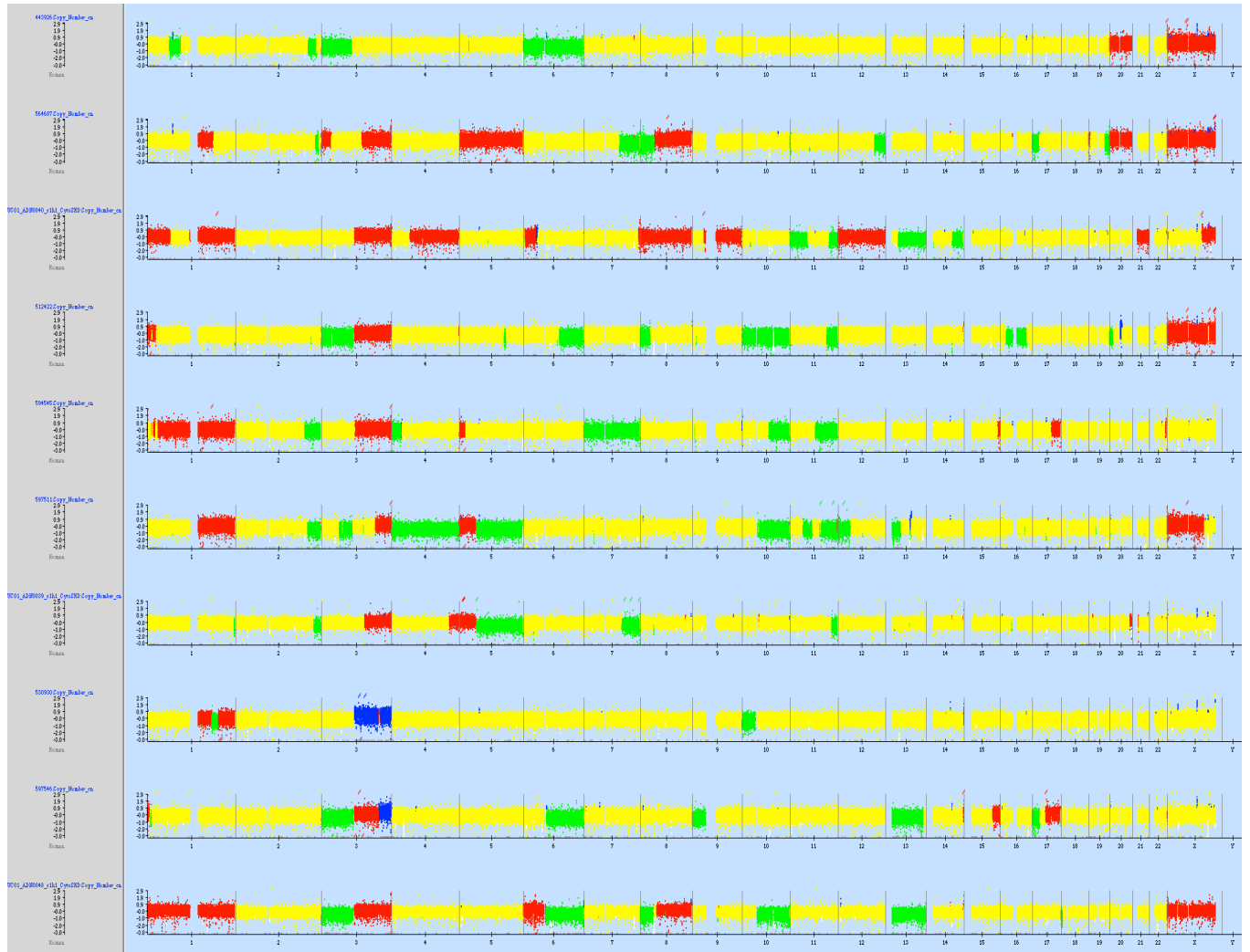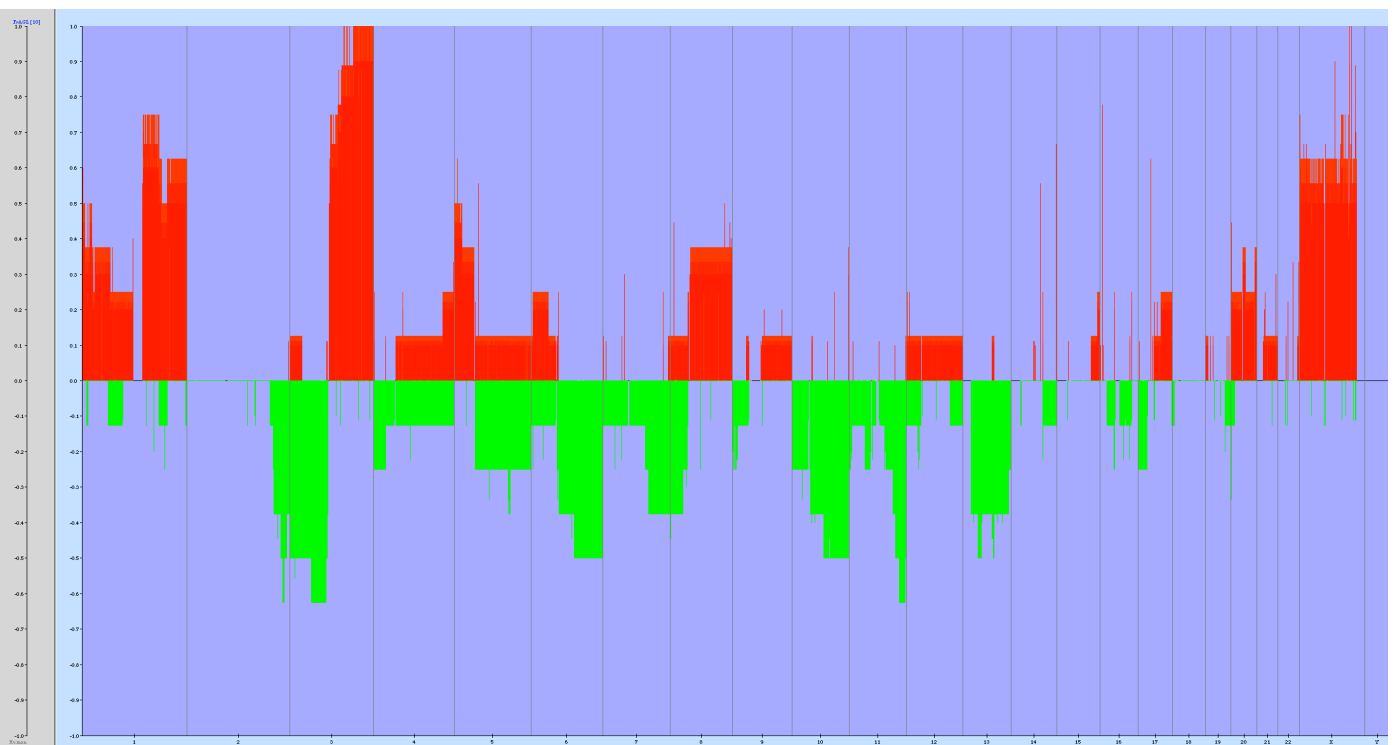

Supplement: Supplementary Figure S7 FrAGL [file npjgenmed20164-s9.pdf]

Fig S8

Case 21 (MJ-CL)

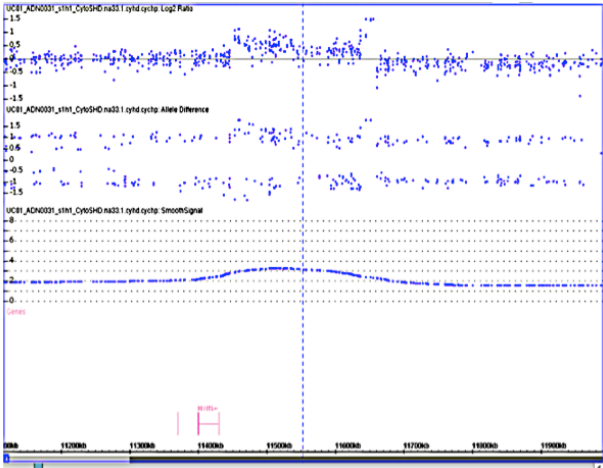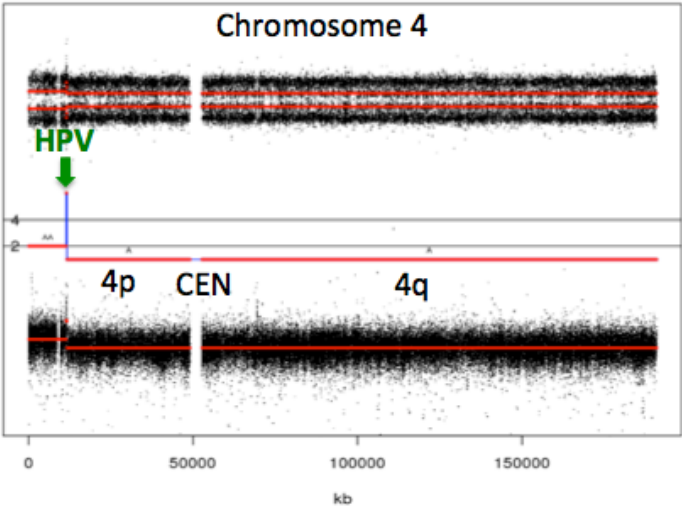

Case 22 (MJ-CL)

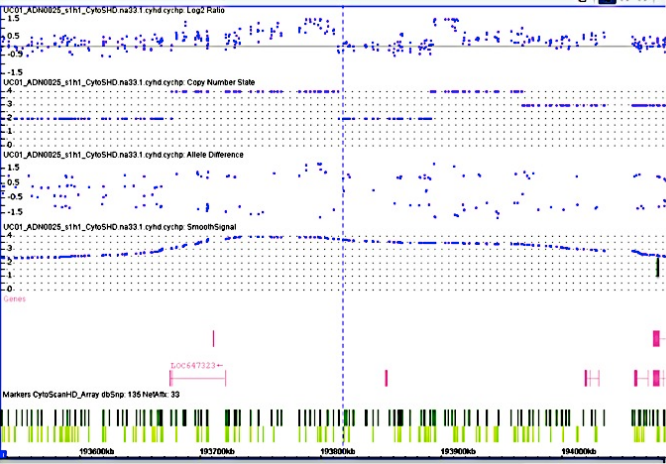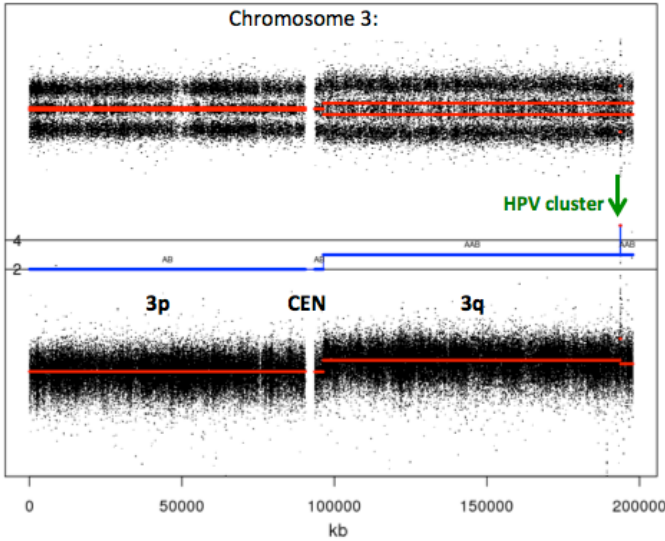

Case 29 (MJ-CL)

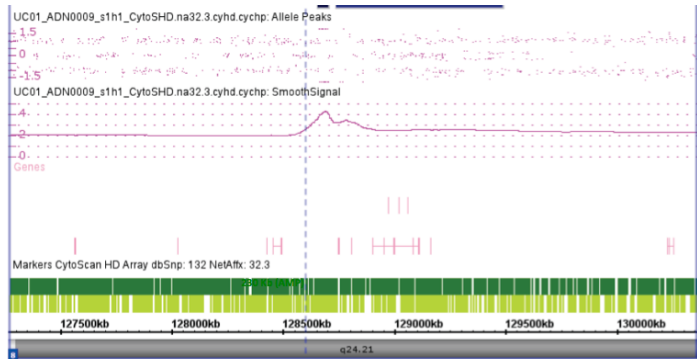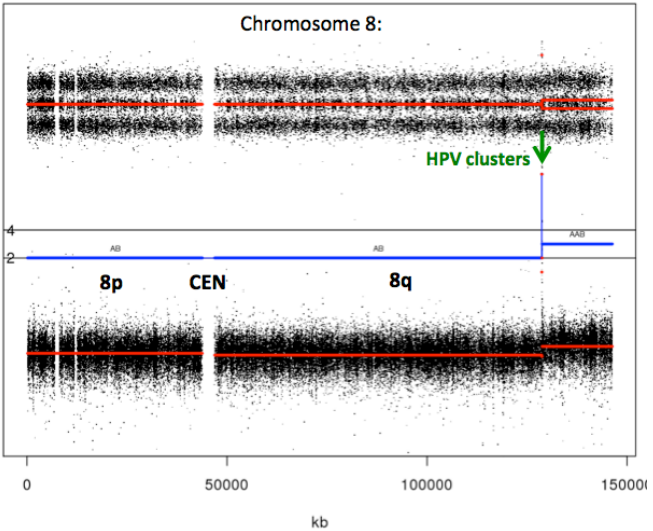

Supplement: Supplementary Figure S8 MJ-CL CGH [file npjgenmed20164-s10.pdf]

Fig S9

Case 31 (MJ-SC)

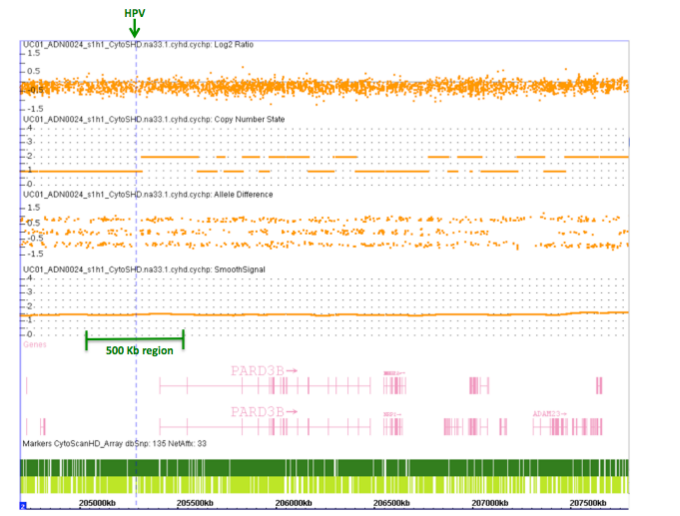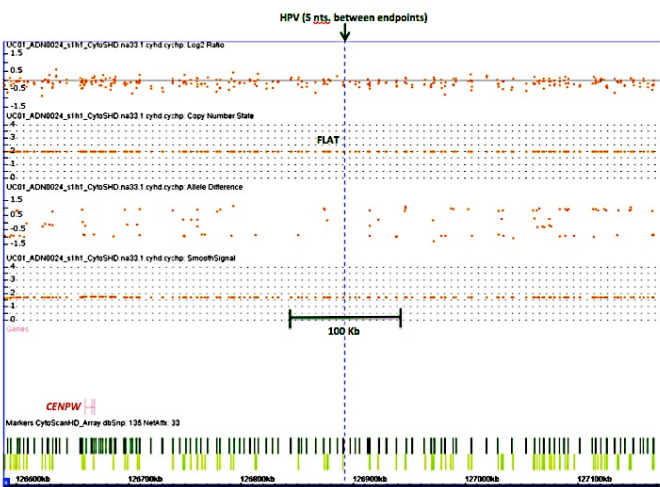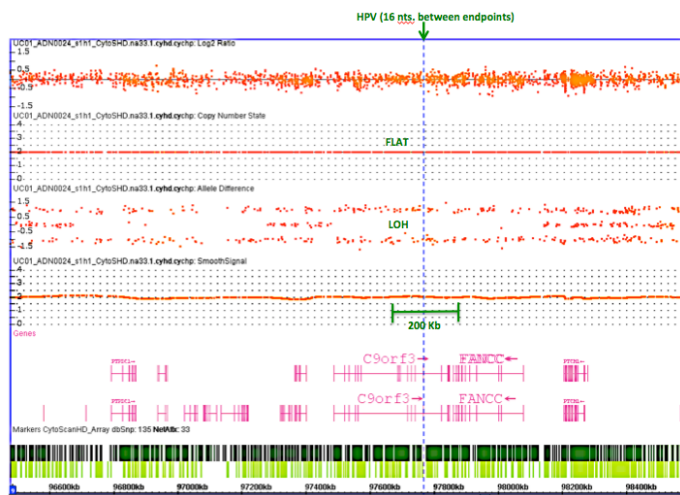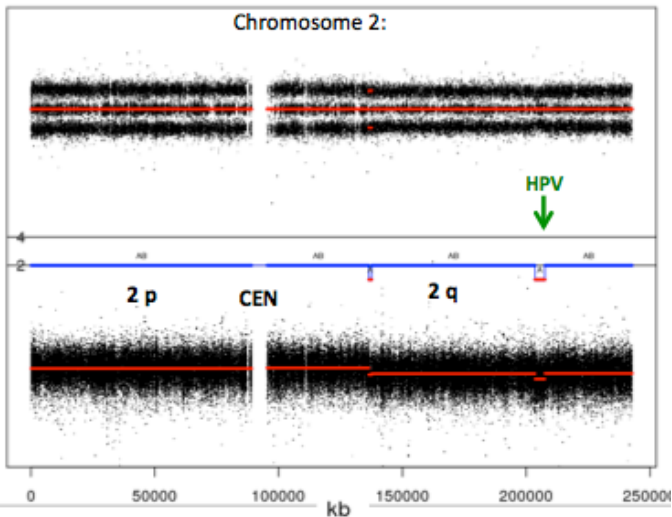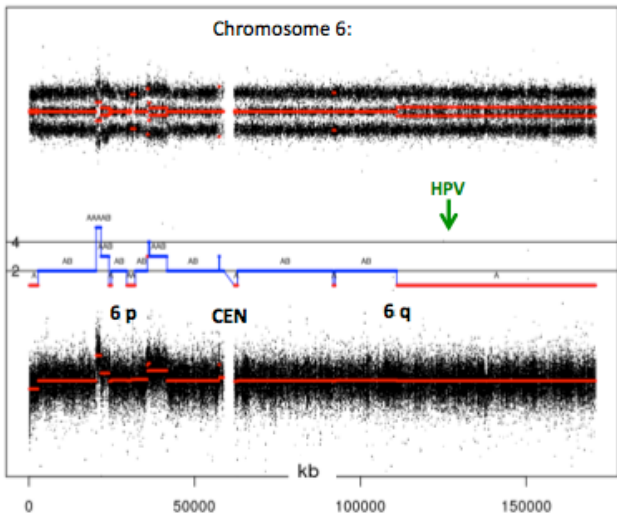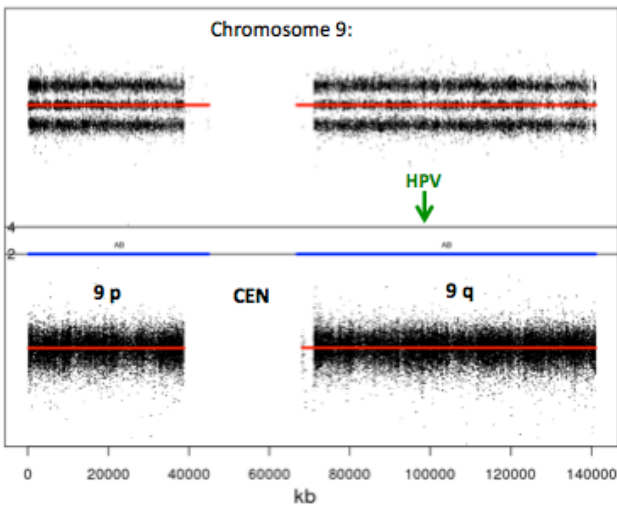

Fig S9

Case 31 (MJ-SC)

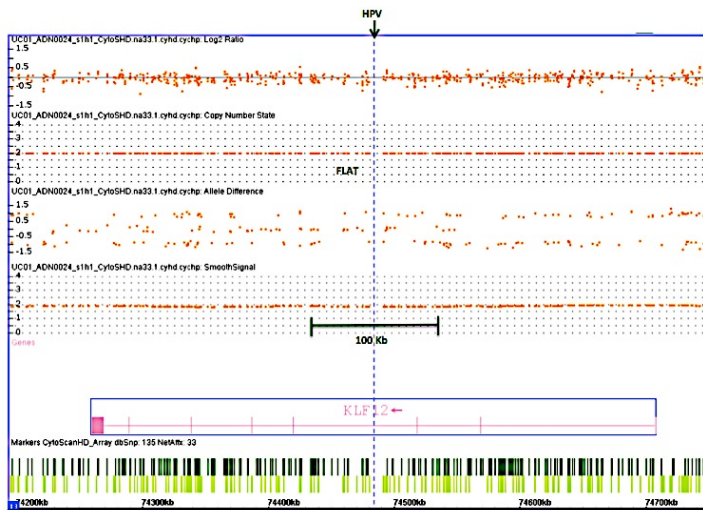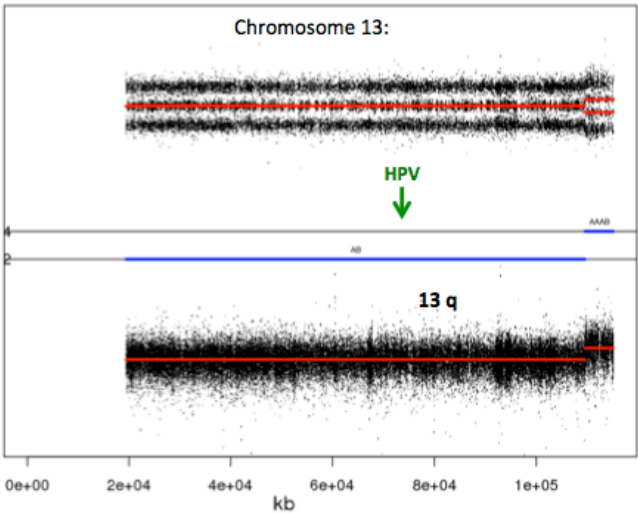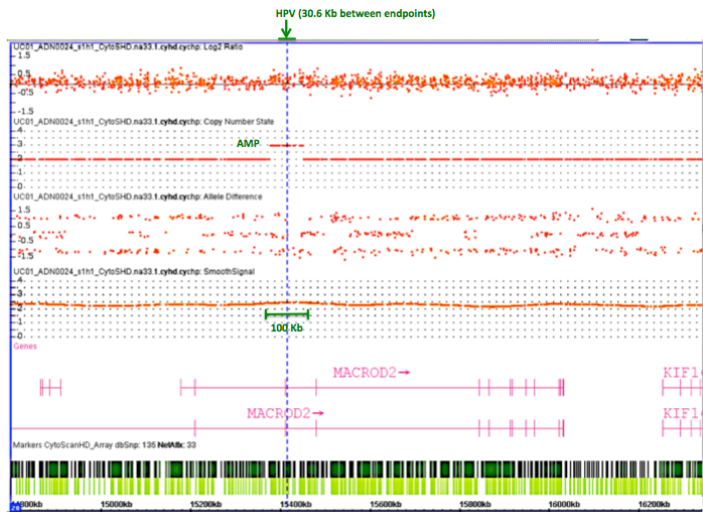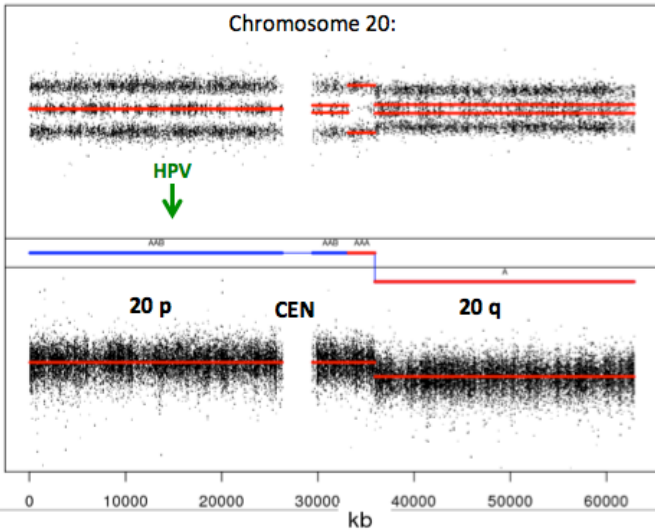

Supplement: Supplementary Figure S9 MJ-SC CGH [file npjgenmed20164-s11.pdf]

Fig S10

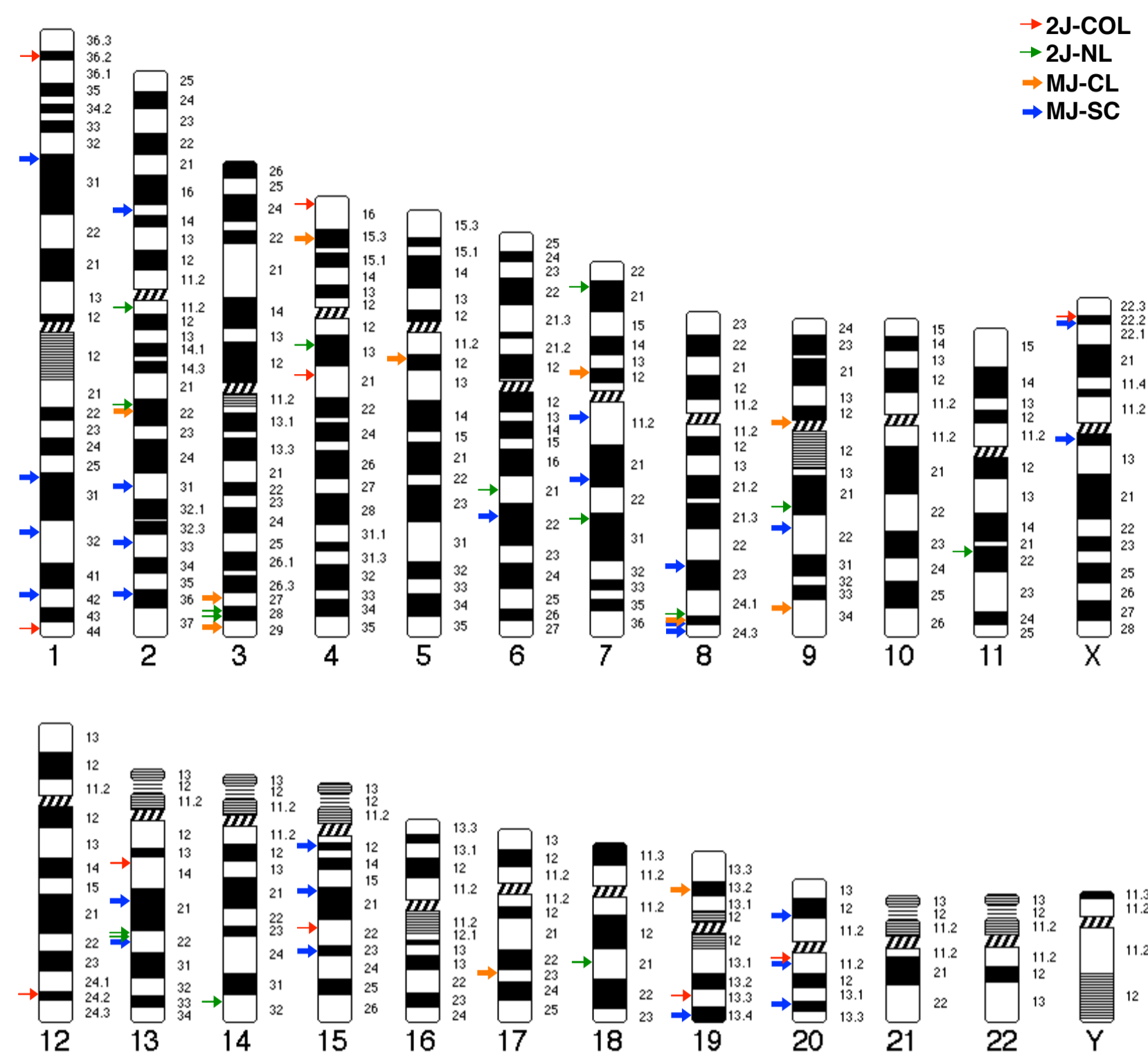

Supplement: Supplementary Figure S10 Distribution HPV insertion sites [file npjgenmed20164-s12.pdf]

Fig S12

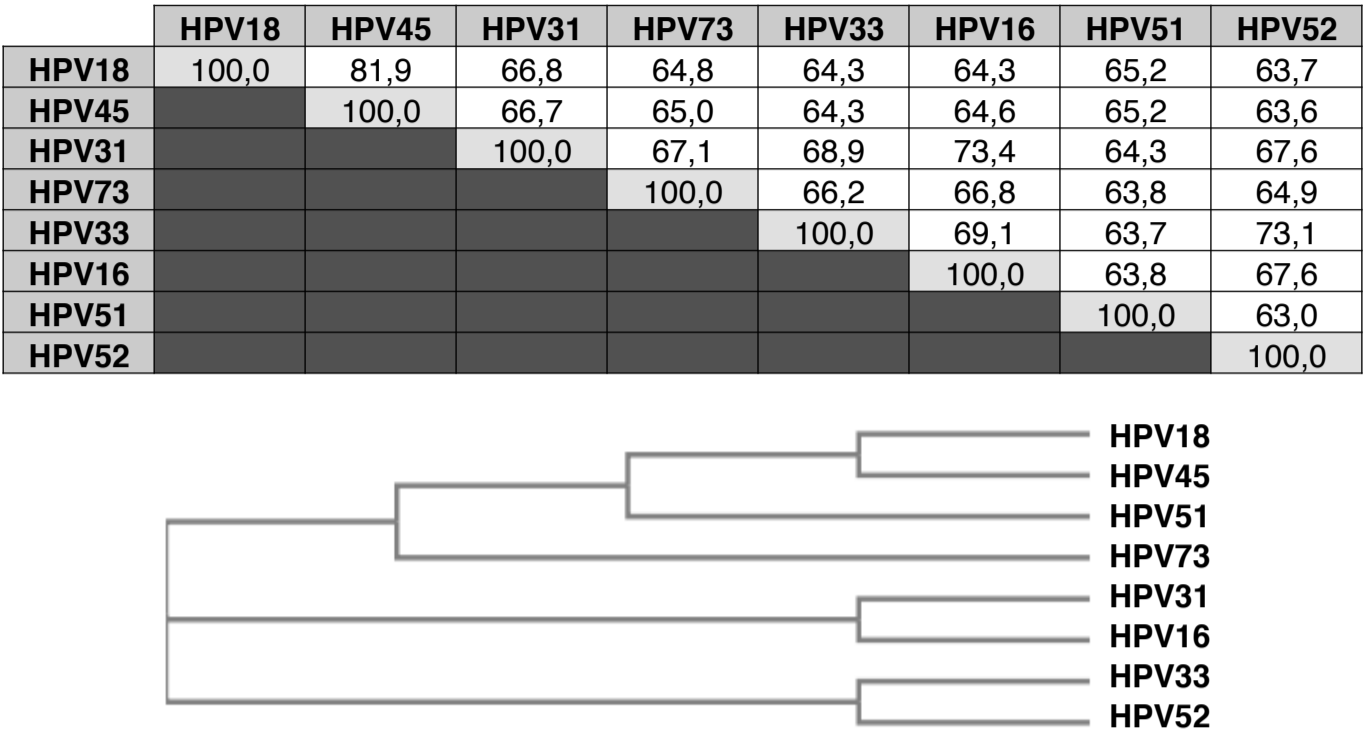

Supplement: Supplementary Figure S12 HPV divergence [file npjgenmed20164-s14.pdf]

A

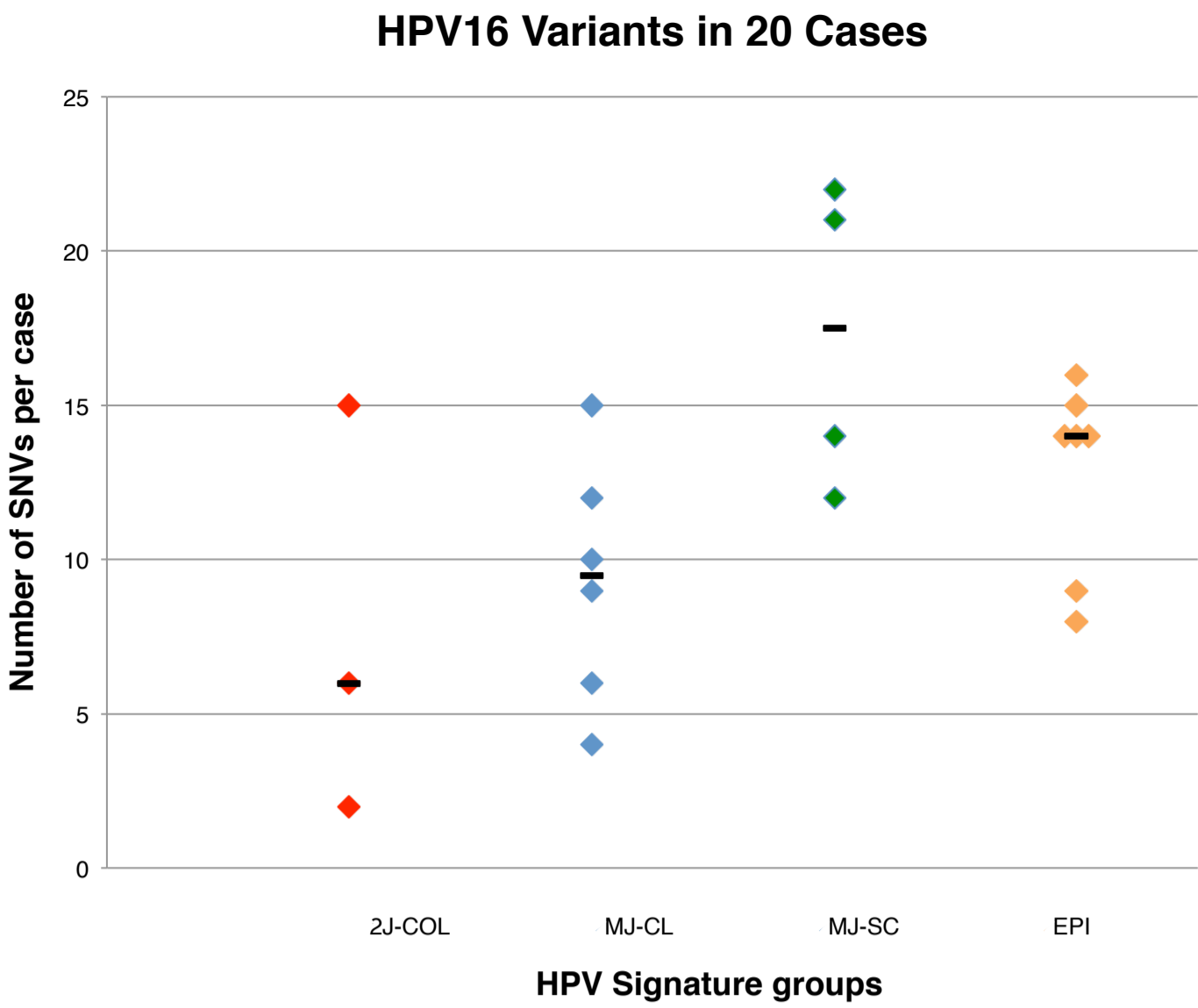

B

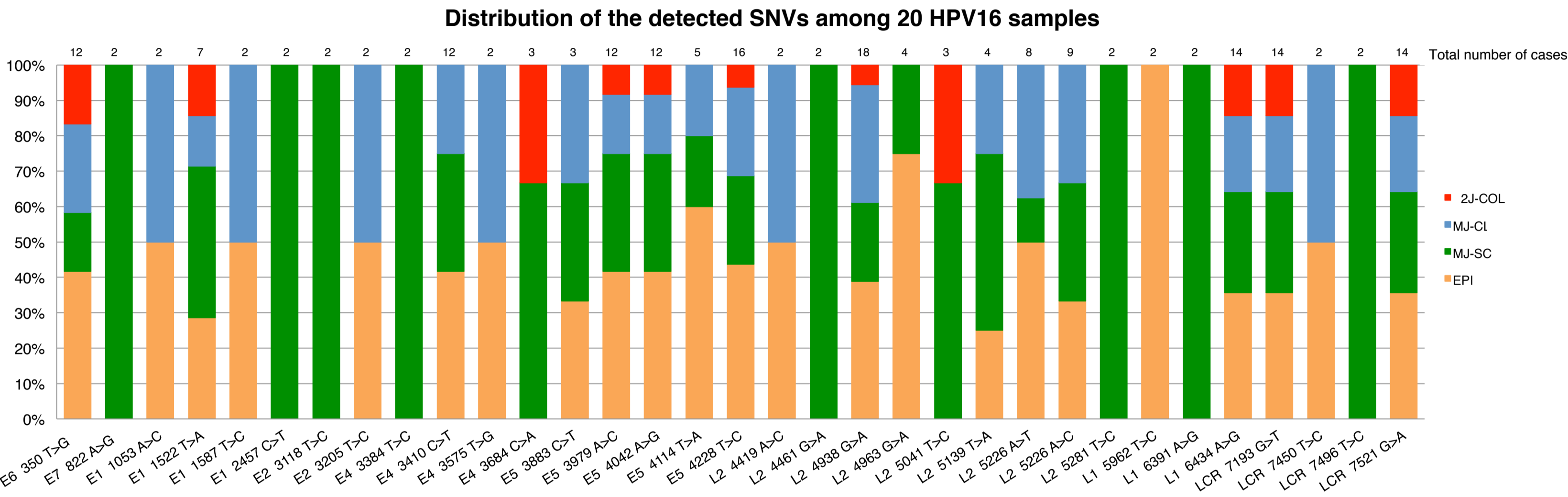

Supplement: Supplementary Figure S13 HPV16 variants [file npjgenmed20164-s15.pdf]

Figure S14 (revised)

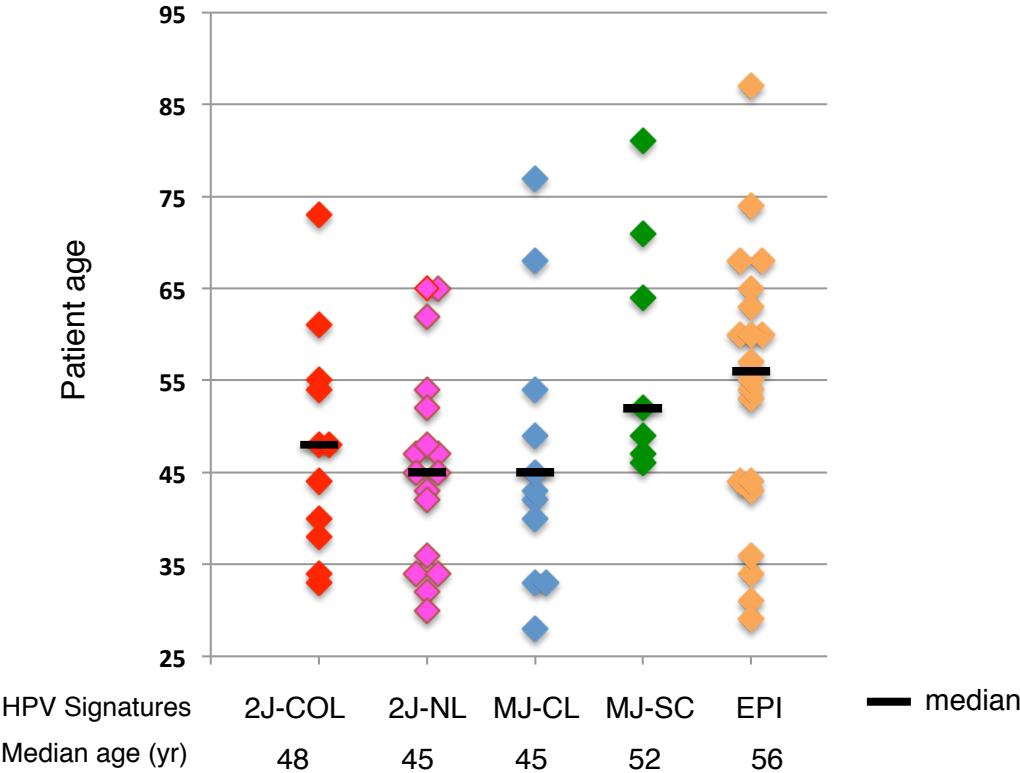

Supplement: Supplementary Figure S14 Age distributions [file npjgenmed20164-s16.pdf]
