# Supplementary material for: Mechanistic signatures of HPV insertions in cervical carcinomas
Source: NPJ Genom Med. 2016 Mar 16;1:16004–. doi: 10.1038/npjgenmed.2016.4 (PMC5685317; doi:10.1038/npjgenmed.2016.4)
Supplement: Supplementary Figure S2 Breakpoints [file npjgenmed20164-s4.pdf]

Fig S2

Case 1 (2J-COL):

Junction 1: Chr13q14.2 (48, 993, 666) / HPV16 E4 (3584)

Chr13q14.2: TCCTTCAGTTCTTCTCTGACCTTGGTTATTTCTTATCTTCTGCTAGC

HPV16 E4: TGCATTTAACAGCTCACACAAAGGACGGATTAACTGTAATAGTAACA

JxChr13/HPV16: TCCTTCAGTTCTTCTCTGACCTTGACGGATTAACTGTAATAGTAACA

Junction 2: HPV16 E2 (3224) / Chr13q14.2 (48, 591, 279)

HPV16 E2: AGGGTCAAGTTGACTATTATGGTTTATATTATGTTCATGAAGGAATA

Chr13q14.2: CTCCACATCCTCTCCAGCACCTGTTGTTTCCTGACTTTTTAATGATCG

Jx HPV16 / Chr13: AGGGTCAAGTTGACTATTATGGTTGTTTCCTGACTTTTTAATGATCG

Case 2 (2J-COL):

Junction 1: ChrXp22.2 (13, 772, 242) / HPV16 LCR (7244)

ChrXp22.2: CAGCCTCCCCAGTAGCTGGGACTACAGGCACCCACCAACACGCCCGGCTA

HPV16 LCR: TGTGCTTGTAAATATTAAGTTGTATGTGTGTTTGTATGTATGGTATAATAAAC

Jx ChrX/HPV16: CAGCCTCCCCAGTAGCTGGGACTACATGTTTGTATGTATGGTATAATAAAC

Junction 2: HPV16 E1 (1333) / ChrXp22.2 (13, 778, 432)

HPV16 E1: GCCATGAGACTGAAACACCATGTAGTCAGTATAGTGGTGGAAGTGGGGGTG

ChrXp22.2: ATCCAACTGCATGGGTGGAGGGTAGTTCCCCTGATTCTGACCTTGAGTTTGT

Jx HPV16/ ChrX: GCCATGAGACTGAAACACCATGTAGTTCCCCTGATTCTGACCTTGAGTTTGT

Case 3 (2J-COL):

Junction 1: Chr1q44 (244,237,555) / HPV18 E4 (3605)

Chr1q44: ACAAGTCAACGGGACTTGGGGACTGATGGGGCTGGGTGGCTGGCGGCAGGGT

HPV18 E4: AGCAGCATTGTGGACCTGTCAACCCACTTCTCGGTGCAGCTACACCTACAGGC

Jx Chr1/HPV18: ACAAGTCAACGGGACTTG*TCAACCCACCTCCAAATA*CAACCCACTTCTCGGTGC

Junction 2: Chr1q44 (244, 436, 006) / HPV18 L2 (4258)

HPV18 E5: TAATAAAAGTATGGTATCCCACCGTGCCGCACGACGCAAACGGGCTTCGGTAA

Chr1q44: TTACTCTCCAATGTGACAGTATTTGGAGGTGGGGCCTTTGGGGGATGATCAGG

Jx HPV18/Chr1: TAATAAAAGTATGGTATCCCACCGTGAGGTGGGGCCTTTGGGGGATGATCAGG

Case 5 (2J-COL):

Junction 1: Chr4q21.3 (87,581,814) / HPV16 L2 (5427)

Chr4q21.3: TGTTTCCAGGGAACAAATGCGTAATTTCACACAGTTCAAACACATTTAACTTAA

HPV16 L2: CATCTTTATCAGGTTATATTCCTGCAAATACAACAATTCCTTTTGGTGGTGCAT

Jx Chr4/HPV16: TGTTTCCAGGGAACAAATGCGTAATTTGTACAACAATTCCTTTTGGTGGTGCAT

Junction 2: Chr4q21.3 (87,230,060) / HPV16 E1 (2410)

HPV16 E1: CCATTTTTGGTTACAACCATTAGCAGATGCCAAAATAGGTATGTTAGATGATGCTA

Chr4q21.3: ATTCATCACCATATTGAGAATTTTTAGTATTATGAAATGAAATGAAGTTTACTGAAT

Jx HPV16/Chr4: CCATTTTTGGTTACAACCATTAGCAGATGTATGAAATGAAATGAAGTTTACTGAAT

Case 17 (2J-COL):

Junction 1: Chr4p16.1 (8, 638, 831) / HPV18 E2 (3013)

Chr4p16.1: CTTTTATTAAGTTCTTATATTTCTAAATAACTTGGCTTTTATAGAGTTACCTGCT

HPV18 E2: TTAAACCACCAGGTGGTGCCAGCCTATAACATTTCAAAAAGTAAAGCACATAA

Jx Chr4/HPV18: CTTTTATTAAGTTCTTATATTTCTAAATAACATTTCAAAAAGTAAAGCACATAA

Junction 2: HPV18 E1 (2289)/ Chr4p16.1 (8,626,438)

HPV18 E1: GCAATTCCTGCGATACCAACAAATAGAGTTTATAACATTTTTAGGAGCCTT

Chr4p16.1: TCTTCCCTTCATCCTTGCCAACCTGGACATGTCTTTCTTTCTCTGTGACCC

Jx HPV18 /Chr4: GCAATTCCTGCGATACCAACAAATAGACATGTCTTTCTTTCTCTGTGACCC

Case 11 (2J-NL):

Junction 1: Chr6q21 (113,335,611) / HPV18 E5 (3861)

Chr6q21: GGCTCCTTAGCACATTTCACCTGCTGTAATTTTTCGTCCTTTTTGTCCTTTTTTGT

HPV18 E5: AGTGAAACACAAAGAACAAAATTTTTAAATACTGTTGCAATTCCAGATAGTGTAC

Jx Chr6/HPV18: GGCTCCTTAGCACATTTCACCTGCTGTAATACTGTTGCAATTCCAGATAGTGTAC

Junction 2: Chr6q21 (113,390,769) / HPV18 E4 (3612)

HPV18 E4: AAGCAGCATTGTGGACCTGTCAACCCACTTCTCGGTGCAGCTACACCTACAGGCA

Chr6q21: TGTTTTTATTAGCTGCGTGAGAATGGACGAATACCCGTTTAGGTTTTTTTTTCTTTT

Jx HPV18/Chr6: AAGCAGCATTGTGGACCTGTCAACCCACAATACCCGTTTAGGTTTTTTTTTCTTTT

Case 13 (2J-NL):

Junction 1: Chr13q22.1 (73,830,702) / HPV18 E2 (3802)

Chr13q22.1: GATTTCCTCTCAGGAAATGAAAGGAAAGGAAACTAAAGGAGAGGAGACGGGA

HPV18 E2: CACCTGGCATTGGACAGGTGCAGGCAATGAAAAAACAGGAATACTGACTGTA

Jx Chr13/HPV18: GATTTCCTCTCAGGAAATGAAAGGAAAGGAAAAAACAGGAATACTGACTGTA

Junction 2: Chr13q22.1 (74,152,642) / HPV18 E1 (1842)

HPV18 E1: TTAAGTACATTGTTACACGTACCTGAAACTTGTATGTTAATTCAACCACCAAAA

Chr13q22.1: TTTCAAATCTATTTTCCTCACAGTGAGTTTTCTTAAAAAAAAAATCTATTTCGA

Jx HPV18/Chr13: TTAAGTACATTGTTACACGTACCTGAAACTTCTTAAAAAAAAAATCTATTTCGA

Case 19 (2J-NL):

Junction 1: Chr7p21.1 (17,531,668) / HPV18 L2 (4453)

Chr7p21.1: AACACAAACACATTACAAATGGACTTTAATTATGTTCCAAATGTGATTTCTTTGCT

HPV18 L2: TGGCAGTGGTACAGGGGGTCGTACAGGGTACATTCCATTGGGTGGGCGTTCCAA

Jx Chr7/HPV18: AACACAAACACATTACAAATGGACTTT*CCATTT*GTACATTCCATTGGGTGGGCGT

Junction 2: Chr7p21.1 (17,352,218) / HPV18 E1 (1180)

HPV18 E1: GTGTTGCATGTTTTAAAACGAAAGTTTGCAGGAGGCAGCACAGAAAACAGTC

Chr7p21.1: GCTTCAAGTAAAGGAGATCTCACAGTCTATCTTATAAAGTTTTATCTTATAAAG

Jx HPV18/Chr7: GTGTTGCATGTTTTAAAACGAAAGTTTTATCTTATAAAGTTTTATCTTATAAAG

Fig S2 (continued)

Case 23 (MJ-CL):

Junction 1: Chr2q22.3 (146,417,585) / HPV16 E1 (1081)

HPV16 E1: GAGACAGCACATGCGTTGTTTACTGCACAGGAAGCAAAACAACATAGAGATGC

Chr2q22.3: AGCATATACTGTAAAAAAAAAGAGTCTAAAATAGATCAAAATTGTGGTTAACAAA

Jx HPV16/ Chr2: GAGACAGCACATGCGTTGTTTACTG*TCATGTTA*AAAATAGATCAAAATTGTGGTT

Junction 2: Chr2q22.3 (146,422,052) / HPV16 E1 (1181)

Chr2q22.3: AATCAAATTTTTTTTAAAAGAAGATCAATAACTTTTTGAAGACGAAGTATTGA

HPV16 E1: GTGATATTAGTGGATGTGTAGACAATAATATTAGTCCTAGATTAAAAGCTATA

Jx Chr2/HPV16: AATCAAATTTTTTTTAAAAGAAGATCAATATTAGTCCTAGATTAAAAGCTATA

Case 24 (MJ-CL):

Junction 1: Chr3q27.3 (187,613,922) / HPV31 E2 (2805)

HPV31 E2: ATTGGAAACATATTCGACTTGAATGTGTATTAATGTATAAAGCAAGAGAAATG

Chr3q27.3: TAATGCTAGATGACGAGTTAGTGGGTGTAATGCACCAGCATGGCACATGTATA

Jx HPV31/ Chr3: ATTGGAAACATATTCGACTTGAATATGTAATGCACCAGCATGGCACATGTATA

* SNP in HPV31 position 2805 (G>A)

Junction 2: Chr3q27.3 (187,600,502) / HPV31 E2 (3664)

Chr3q27.3: ATCAAGATCTAGAACAGTTGCATTACCCAAAATATTGCTTCTTACCCCTCTGTG

HPV31 E2: ATTGTATGAACAAGTGTCATCTACATGGCATTGGACATGTACAGATGGAAAACAT

Jx Chr3/HPV31: ATCAAGATCTAGAACAGTTGCATTACGCATTGGACATGTACAGATGGAAAACAT

Case 28 (MJ-CL):

Junction 1: Chr5q12.3 (66,508,046) / HPV16 E6 (331)

HPV16 E6: AAAGTTTTATTCTAAAATTAGTGAGTATAGACATTATTGTTATAGTTGTATG

Chr5q12.3: CCAATGCTCCAGCTCATGCAGTCACGTAAGAGAAATTCCCTCTTACTTGGCCT

Jx HPV16/ Chr5: AAAGTTTTATTCTAAAATTAGTGAGTATAGAGAAATTCCCTCTTACTTGGCCT

Junction 2: Chr5q12.3 (66,547,382) / HPV16 E1 (1516)

HPV16 E6: AATTTAAAGAGTTATACGGGGTGAGTTTTTCAGAATTAGTAAGACCATTTAAAAG

Chr5q12.3: TTCTTCCTACCCATGAGCATGGAATGTTCTTCCATTTGTTTGTATTCTCTTTTATT

Jx HPV16/ Chr5: AATTTAAAGAGTTATACGGGGTGAGTTCTTCCATTTGTTTGTATTCTCTTTTATT

*SNP in HPV16 position 1520 (T>C)

Case 29 (MJ-CL):

Junction 1: Chr8q24.21 (128,676,026) / HPV16 E1 (1473)

Chr8q24.21: GAAAAAAAACCCATCAAATCCCTCTGCCTTGTGGGTCTCACAGGAGGCAAACT

HPV16 E1: TTTTAAATGTACTAAAAACTAGTAATGCAAAGGCAGCAATGTTAGCAAAATTTA

Jx Chr8/ HPV16: GAAAAAAAACCCATCAAATCCCTCTGCAAAGGCAGCAATGTTAGCAAAATTTA

Junction 2: Chr8q24.21 (128,760,118) / HPV16 E1 (955)

HPV16 E1: GTAGTGGAAAAAAAAACAGGGGATGCTATATCAGATGACGAGAACGAAAATGA

Chr8q24.21: GCAGGAGCCTGTAATCCCAGCTACTCGGGAGTCTGAGGCAGAGAATTACTTGA

Jx HPV16/ Chr8: GTAGTGGAAAAAAAACAGGGG*CAGAGAATTC*TCTGAGGCAGAGAATTACTTGA

Case 30 (MJ-SC):

Junction 1: ChrXq12 (67,757,299) / HPV31 E2 (2848)

HPV31 E2: AAGAGAAATGGGAATACACAGTATTAACCACCAGGTGGTGCCAGCGTTGTCAGT

ChrXq12: CTGTATACCATGCCAGGCACTTTATTAGTTGCTTTACAAACAGTATCTCTGATTG

Jx HPV31/ ChrX: AAGAGAAATGGGAATACACAGTATT*GTATTGT*GTTGCTTTACAAACAGTATCTCT

Junction 2: Chr20q13.32 (56,885,148) / HPV16 E4 (3355)

HPV31 E4: ATTGTTACAAAGCTACCAACAGCCAACAACACCACCACATCGAATTCCAAAA

Chr20q13.32: CGGATCCCCCCCTGTCCCCTCATCCCTTCCCCCCTCCCACGTCCAAGACGCTG

Jx HPV31/ Chr20: ATTGTTACAAAGCTACCAACAGCCCTTCCCCCCTCCCACGTCCAAGACGCTG

Case 33 (MJ-SC):

Junction 1: Chr1q42.3 (236,318,610) / HPV16 LCR (7164)

Chr1q42.3: AAGTAGAAGCAGCTTAATGAATATTTGTCCTAATGCTTTCACCTCGCAGATAGAC

HPV16 LCR: CGCAAAAAACGTAAGCTGTAAGTATTGTATGTATGTTGAATTAGTGTTGTTTGTT

Jx Chr1/HPV16: AAGTAGAAGCAGCTTAATGAATATTTGTATGTATGTTGAATTAGTGTTGTTTGTT

Junction 2: Chr20q11.21 (30,208,967) / HPV16 E4 (3411)

Chr20q11.21: GGTAGAATGTCTTTCAAGTTGGATTTGTCTGAGATTTTCTTGTGATCAAATT

HPV16 E4: TATTAGGCAGCACTTGGCCAACCACCCCGCCGCGACCCATACCAAAGCCGTC

Jx Chr20/HPV16: GGTAGAATGTCTTTCAAGTTGGATTTCCGCCGCGACCCATACCAAAGCCGTC

Case 34 (MJ-SC):

Junction 1: Chr8q23.1 (107,273,933) / HPV16 E1 (1166)

HPV16 E1: GTCCACTTAGTGATATTAGTGGATGTGTAGACAATAATATTAGTCCTAGATTAAA

Chr8q23.1: TTCTAAGCCTTGGTCACTGGCTGCCTGGAAATACACTTGGTGCTGTTGCGGCAG

Jx HPV16/ Chr8: GTCCACTTAGTGATATTAGTGGATGTGGAAATACACTTGGTGCTGTTGCGGCAG

Junction 2: Chr2q36.3 (228,020,331) / HPV16 L1 (6286)

Chr2q36.3: CGCTGGAGTTCACGCCCAAGGCAATTCCAGGAGGTCTGAAAGGTCCAGAGAATT

HPV16 L1: TGGACTTTACTACATTACAGGCTAACAAAAGTGAAGTTCCACTGGATATTTGTAC

Jx Chr2/HPV16: CGCTGGAGTTCACGCCCAAGGCAACAAAAGTGAAGTTCCACTGGATATTTGTAC
