# Supplementary material for: Mechanistic signatures of HPV insertions in cervical carcinomas
Source: NPJ Genom Med. 2016 Mar 16;1:16004–. doi: 10.1038/npjgenmed.2016.4 (PMC5685317; doi:10.1038/npjgenmed.2016.4)

Fig S6

Case 9 (2J-NL)

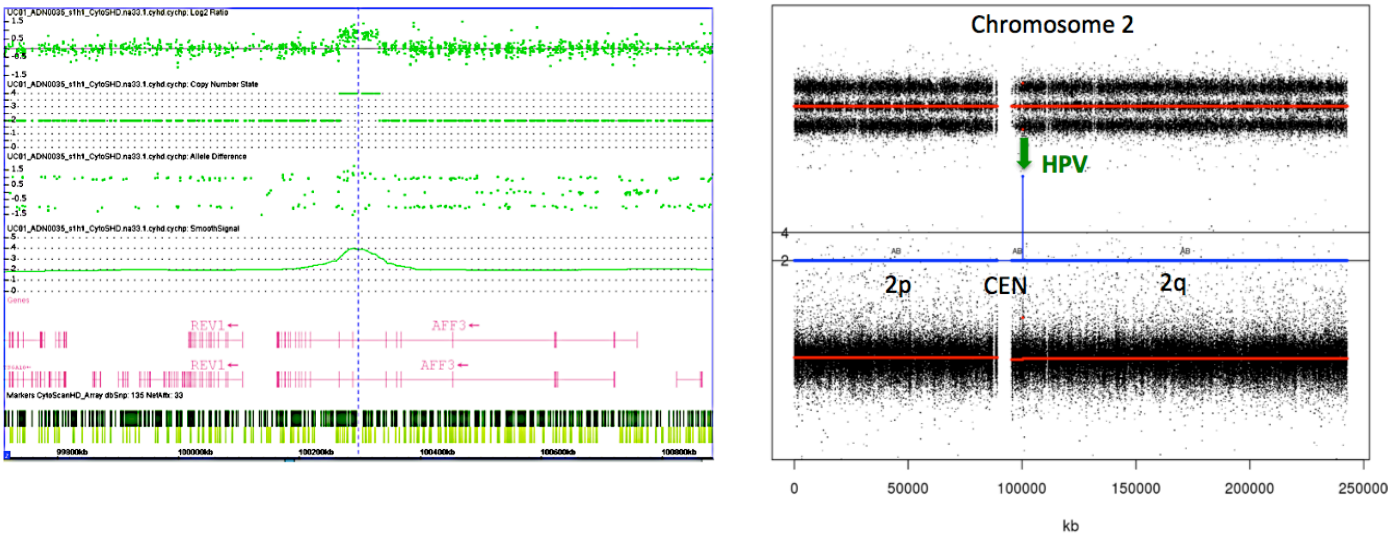

Case 14 (2J-NL)

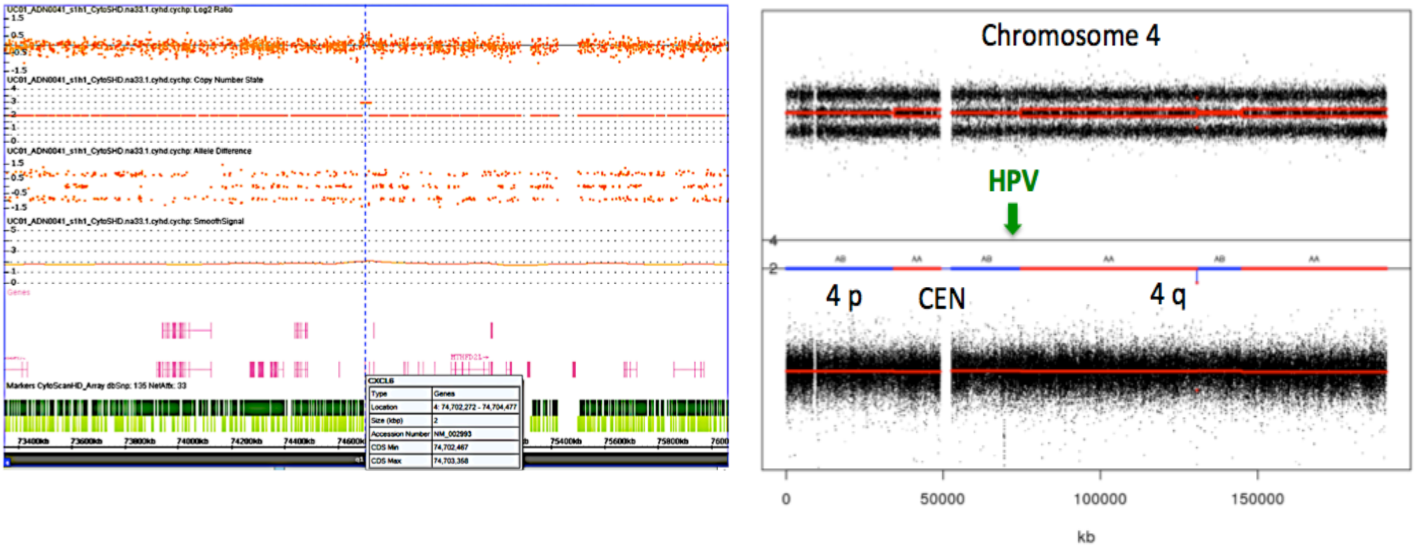

Case 18 (2J-NL)

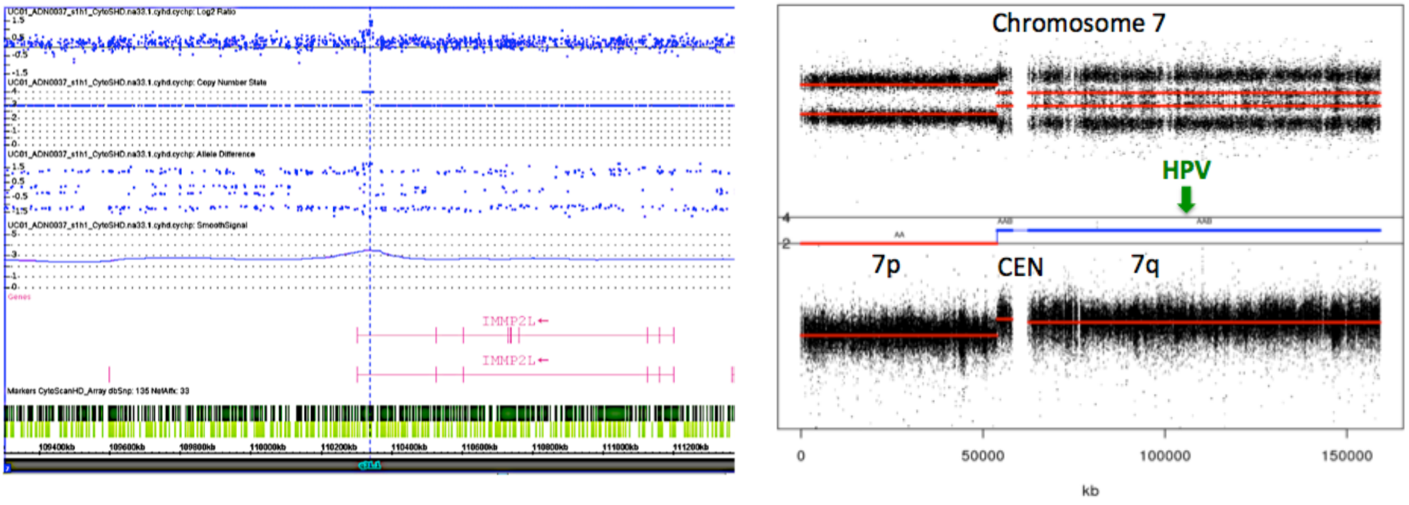

Fig S6

Case 10 (2J-NL)

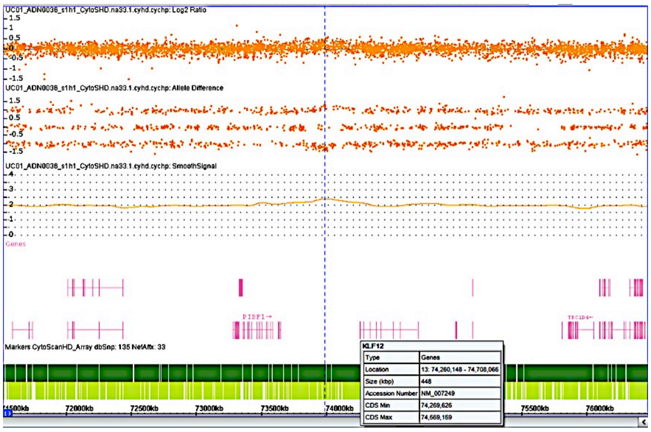

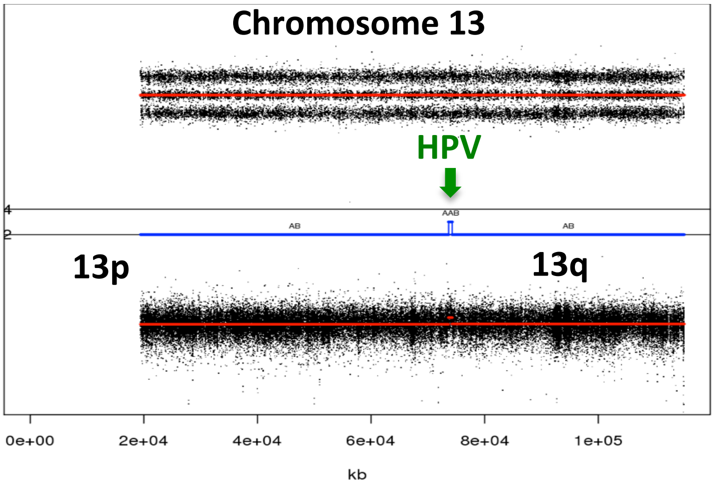

Case 12 (2J-NL)

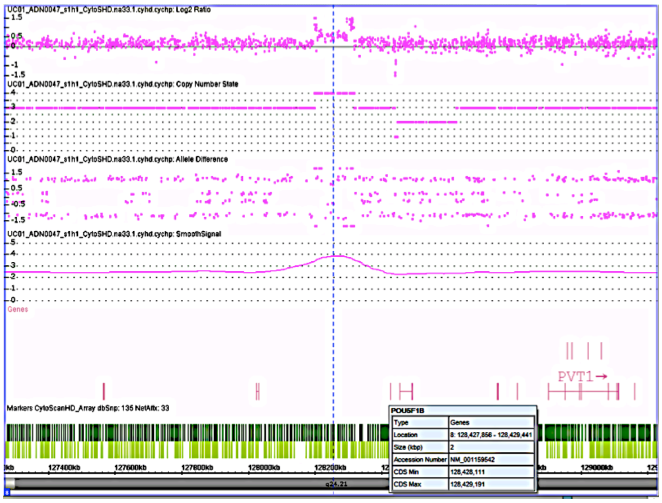

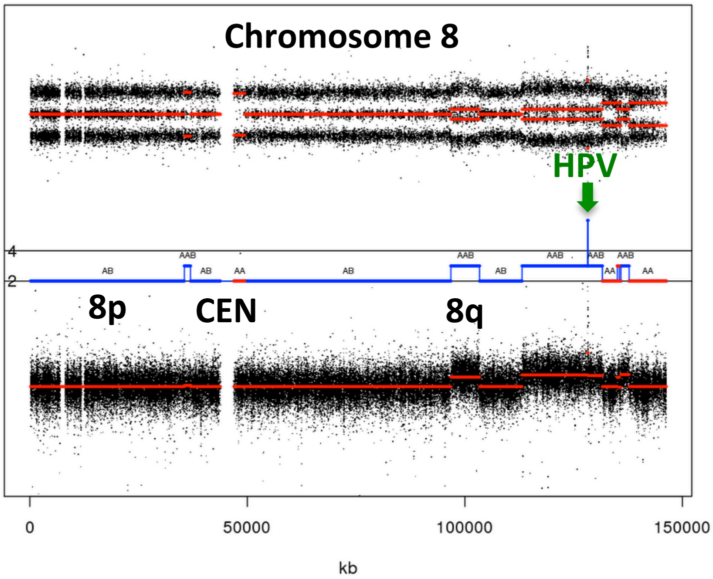

Case 15 (2J-NL)

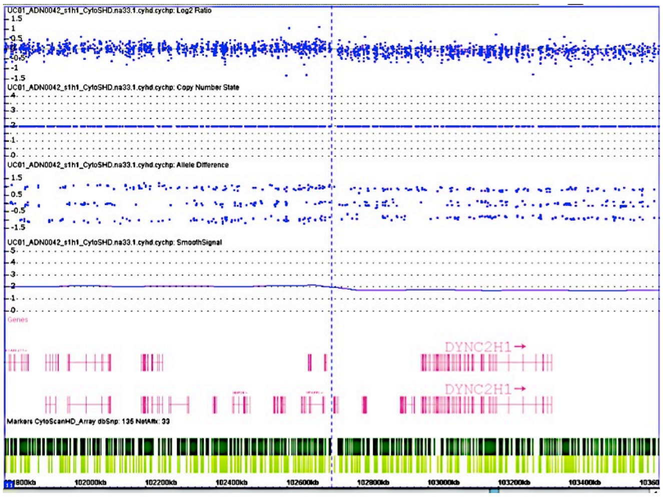

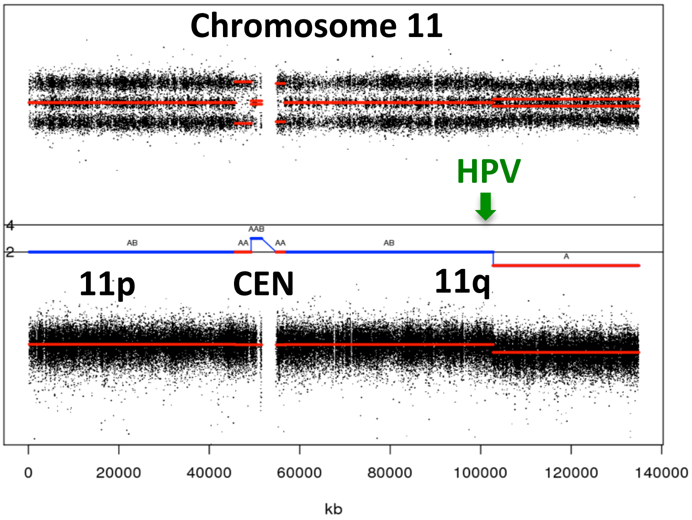

Fig S6

Case 16 (2J-NL)

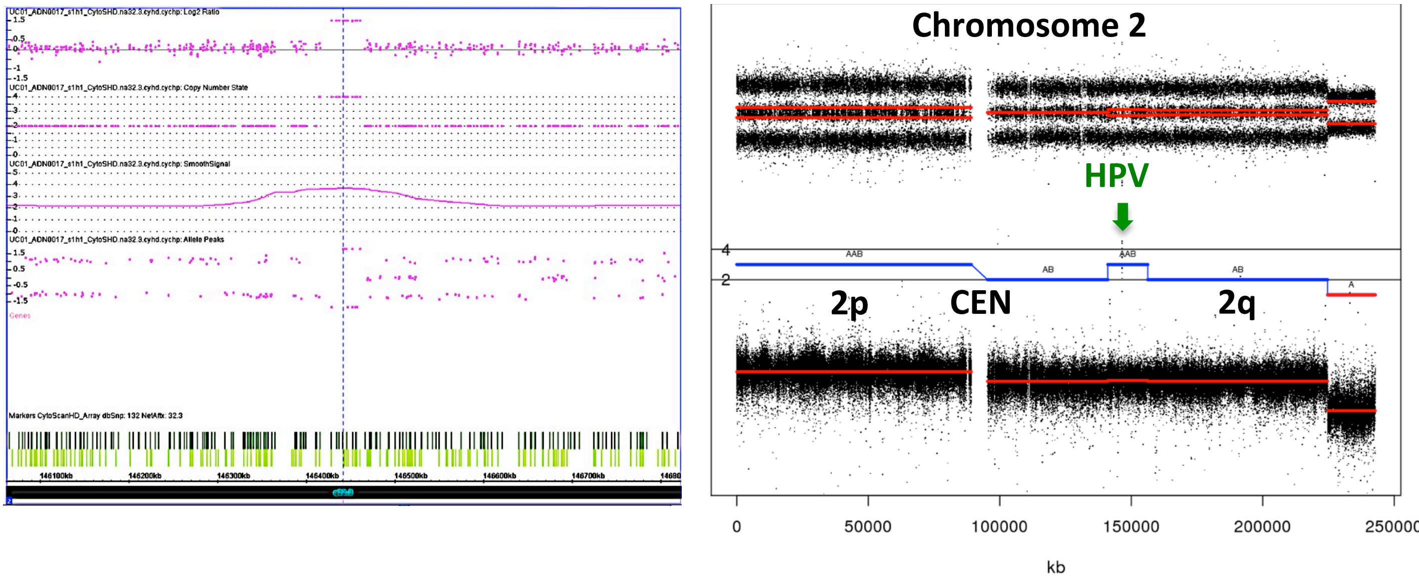

Case 20 (2J-NL)

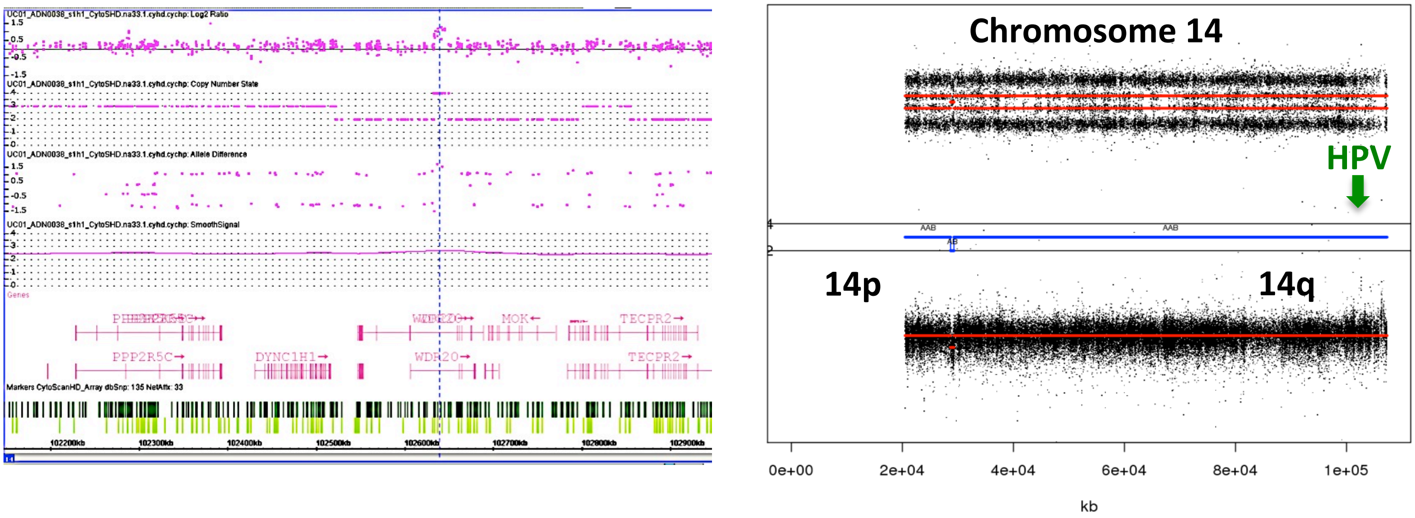

Case 82 (2J-NL)

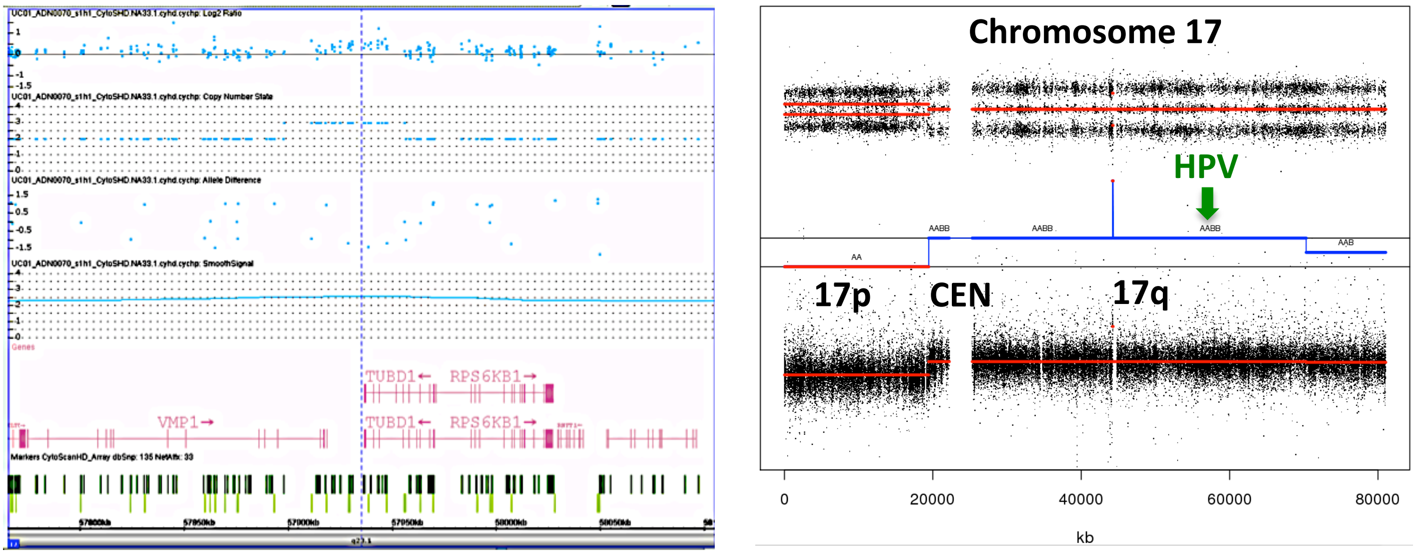

Fig S6

Case 87 (2J-NL)

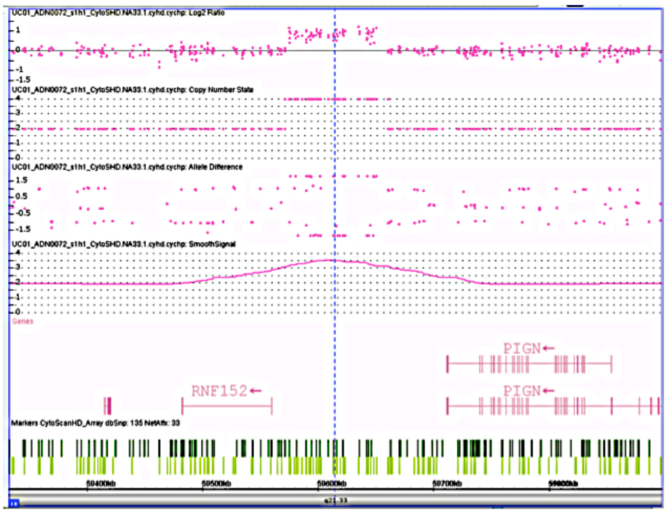

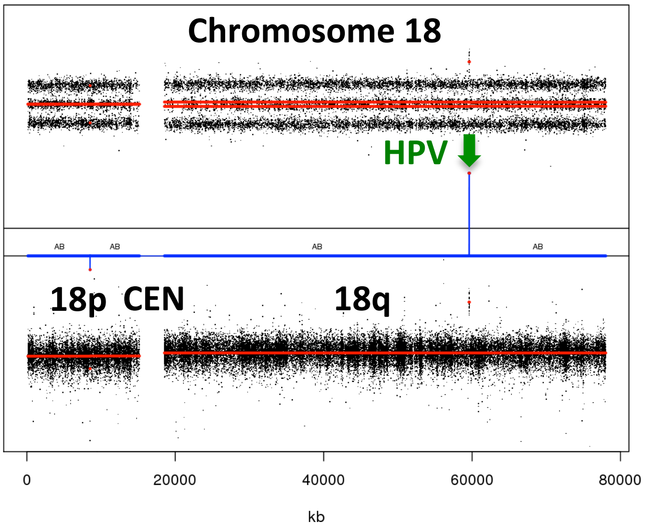

Case 205 (2J-NL)

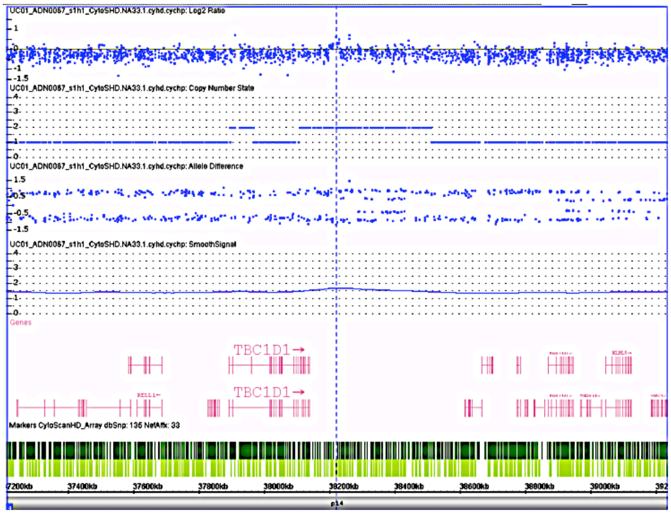

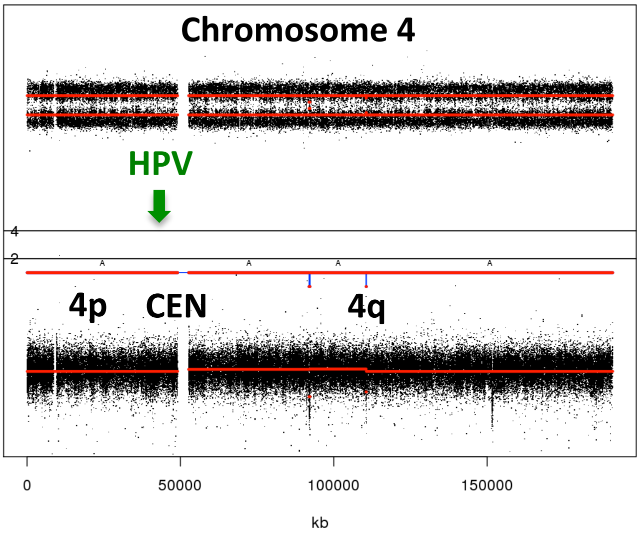

Case 143 (2J-NL)

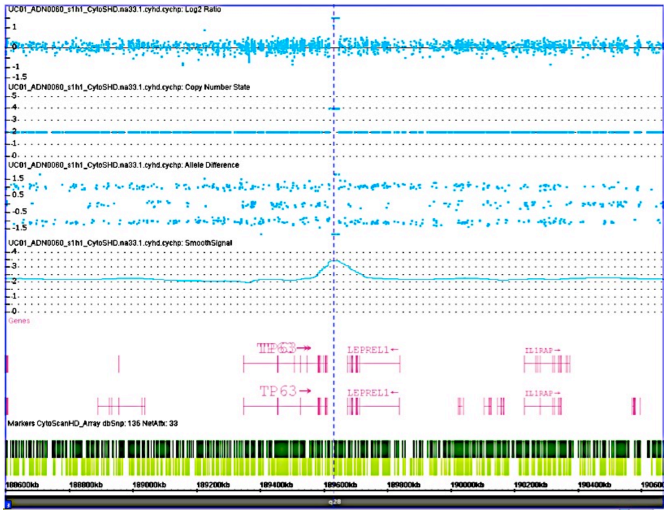

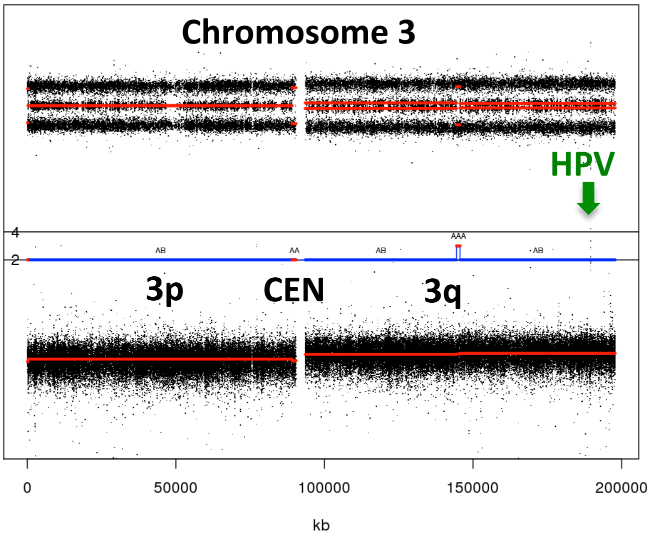

Fig S6

Case 139 (2J-NL)

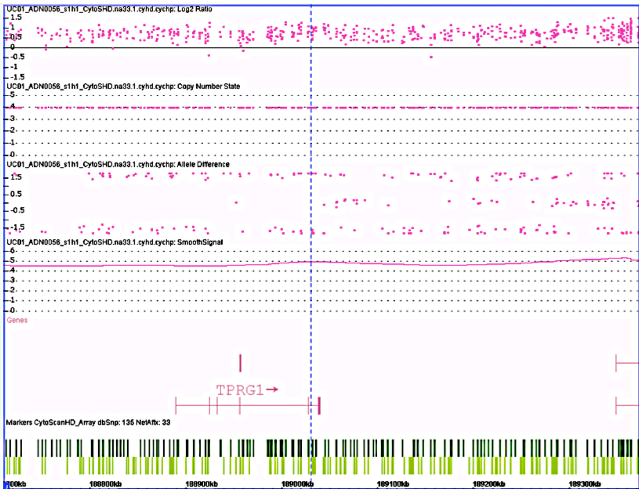

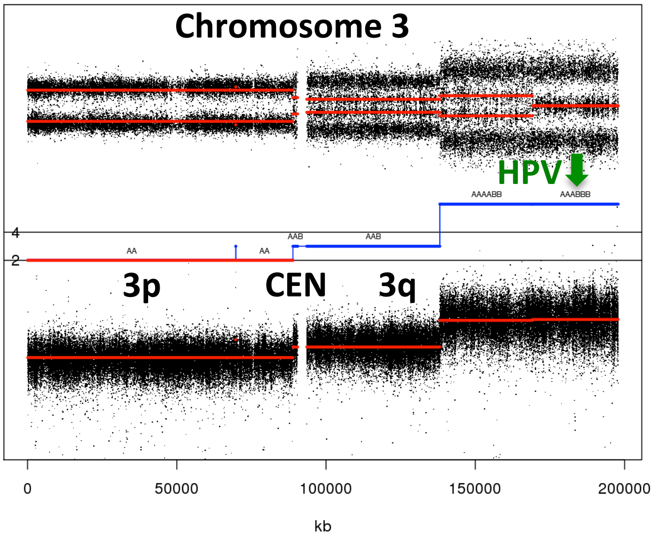

Case 125 (2J-NL)

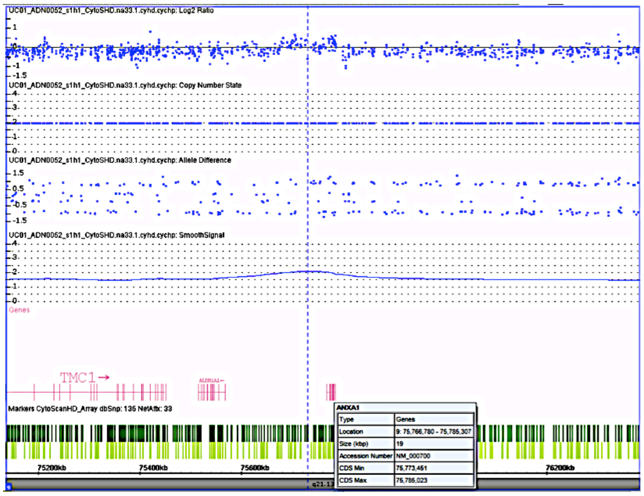

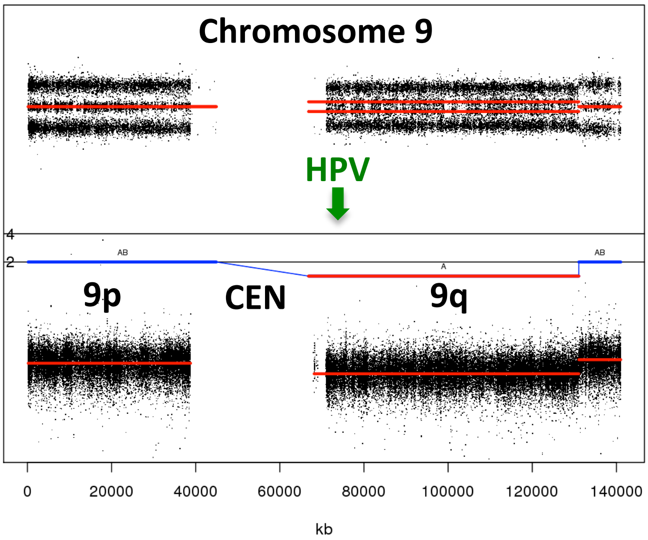

Supplement: Supplementary Figure S6 2J-NL CGH [file npjgenmed20164-s8.pdf]
