# Supplementary material for: Mechanistic signatures of HPV insertions in cervical carcinomas
Source: NPJ Genom Med. 2016 Mar 16;1:16004–. doi: 10.1038/npjgenmed.2016.4 (PMC5685317; doi:10.1038/npjgenmed.2016.4)
Supplement: Supplementary Figure S11 Target genes [file npjgenmed20164-s13.pdf]

Fig S11

Novel target genes of HPV integration and their functional implication.

Transcription:	***MED4***; ***ZNF341***; *ZRANB2; ZFPM2; ZBTP18;* ***AFF3***; *BCL6; BCL2L1; HES1; CCAT1; CCAT2*
Proliferation :	***NUDT15***; *AKT3; SST; ID1;* ***RB1***; ***LPAR6***; ***MAPK10***
Oncogenesis:	***RAB11A*** ; ***RAB22A***
Microtubules:	***MAST4*** ; ***MAP2***
Kinetochore:	*CENPW*
Cell adhesion:	*LPP; NEGR1*
Angiogenesis:	***PF4V1***
Extra-cellular matrix:	***MMP12***; ***COL4A4*** ; *NID1*
Cell metabolism:	*VPS54*

.
